# Cardiovascular ageing: hallmarks, signaling pathways, diseases and therapeutic targets

**DOI:** 10.1038/s41392-026-02630-7

**Published:** 2026-04-21

**Authors:** Pingjing Zheng, Wendi Yan, Yangnan Ding, Yang Zhang, Zhangwei Chen, Juying Qian, Junbo Ge

**Affiliations:** 1 Department of Cardiology, Zhongshan Hospital, Fudan University, Shanghai Institute of Cardiovascular Diseases, Shanghai, China; 2 National Clinical Research Center for Interventional Medicine, Shanghai, China; 3 State Key Laboratory of Cardiovascular Diseases, Zhongshan Hospital, Fudan University, Shanghai, China; 4 NHC Key Laboratory of Ischemic Heart Diseases, Shanghai, China; 5 Key Laboratory of Viral Heart Diseases, Chinese Academy of Medical Sciences, Shanghai, China; 6 Department of Laboratory Medicine, Zhengzhou Key Laboratory for In Vitro Diagnosis of Hypertensive Disorders of Pregnancy, The Third Affiliated Hospital of Zhengzhou University, Zhengzhou, China

**Keywords:** Cardiology, Molecular medicine, Therapeutics, Cardiovascular diseases

## Abstract

Cardiovascular disease (CVD) predominantly affects elderly individuals and is the leading cause of morbidity, disability, and mortality worldwide. Systemic ageing, especially cardiovascular ageing, contributes to the development of CVD phenotypes and outcomes. Therefore, in this review, we innovatively summarize the five major etiologies and risk factors for cardiovascular ageing, including lifestyle and behavioral factors, metabolic disorders and physiological dysregulation, environmental exposures and physicochemical determinants, genetics and epigenetics, and host biology and sociodemographic determinants. Furthermore, we enumerate the structural and functional changes that occur in the cardiovascular ageing process. The twelve hallmarks of cardiovascular ageing, including genomic instability and epigenetic alterations, loss of proteostasis, mitochondrial dysfunction, oxidative stress, and inflammation; cellular dysfunction; cellular senescence; stem cell exhaustion; metabolic changes; and the renin‒angiotensin‒aldosterone system, β-adrenergic signaling, growth signaling, and mechanosignaling, stratify across three dimensions: molecular, cellular, and systemic levels. Given the elucidated role of cardiovascular ageing in diverse pathologies, we propose specific rejuvenation strategies to mitigate residual cardiovascular risk in older adults: targeting senescent cells, adjusting energy sensor pathways, addressing central inflammatory pathways, modulating neurocardiological dynamics, adopting healthy lifestyles, and assessing and preventing the degree of ageing. We also list FDA-approved drugs and clinical trials targeting cardiovascular ageing, thus serving as a cutting-edge reference for developing intervention strategies.

## Introduction

Cardiovascular disease (CVD) is the leading cause of mortality and morbidity worldwide. Between 1990 and 2019, the global number of CVD patients nearly doubled, increasing from 271 million to 523 million, whereas related deaths increased from 12.1 million to 18.6 million.^1^ It is not only the primary contributor to premature death but also imposes a continuously growing economic burden on healthcare systems. Even though the abundance of novel therapies has proven markedly efficient in reducing the cardiovascular mortality, the residual cardiovascular risk remains significantly high. Thus, there is an urgent need to increase CVD prevention and treatment efforts.^2^

In general, CVD risk factors can be broadly categorized into modifiable and nonmodifiable factors. CVD is largely driven by a set of modifiable risk factors, including smoking, unhealthy diet, physical inactivity, excessive alcohol consumption, hypertension, dyslipidaemia, diabetes mellitus, obesity, and psychosocial stress.^1^ In addition, nonmodifiable risk factors include age, sex, and genetics.^3^ Indeed, advancing age is strongly associated with structural and functional decline of the heart and vasculature. Cardiac ageing involves structural changes such as fibrosis and reduced functional reserve.^4–6^ Vascular ageing is characterized by endothelial dysfunction, arterial stiffness and chronic inflammation.^7–9^ Together, they impair cardiovascular adaptability and heighten disease risk. Consequently, older adults bear the greatest burden of CVD, including heart failure, atrial fibrillation, and atherosclerotic complications.^10^ Although the increase in age is irreversible, recent studies challenge the conventional view of ageing as an inevitable consequence of time accumulation, demonstrating that the pace of ageing differs across species, individuals, and even specific organs.^11,12^ These variations are driven by a complex interplay of factors, including lifestyle behaviors, metabolic disorders, environmental exposures, and genetic–epigenetic determinants, which collectively accelerate cardiovascular functional decline. Interventions such as lifestyle modifications, pharmacological treatments, and targeted therapies aimed at ageing-related pathways have been shown to effectively modulate the cardiac ageing process.^13–15^ This emerging perspective views ageing as a modifiable condition driven by multiple pathological factors, suggesting that delaying or even reversing age-related CVD through targeted intervention may be possible.^16^

**Fig. 1:**
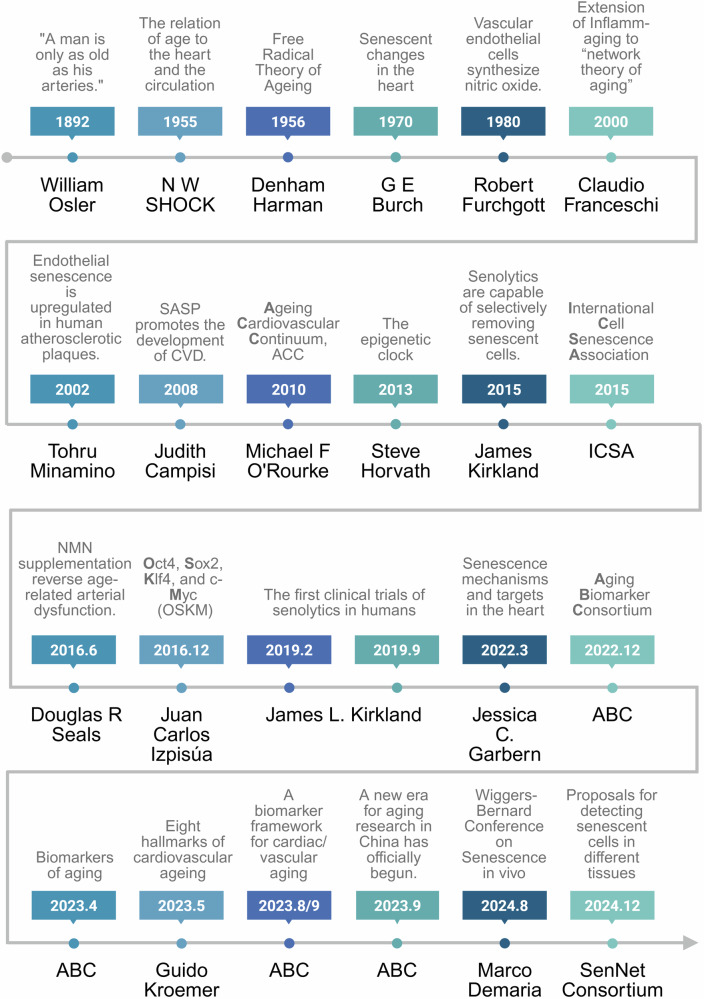
Timelines of key discoveries in cardiovascular ageing research. Since 1892, when William Osler proposed that “a man is only as old as his arteries,” research on cardiovascular ageing formally commenced. Prior to the 21st century, investigations were largely confined to tissue-level changes. The landmark discovery of the senescence-associated secretory phenotype (SASP) in 2008 marked a paradigm shift, demonstrating that ageing research extends beyond senescent cells themselves. Following the 2015 conceptualization of senolytics, targeted therapeutics advanced progressively, culminating in the first published human clinical trials in 2019. China’s 2022 establishment of the ageing Biomarker Consortium (ABC) yielded its foundational consensus document, Biomarkers of ageing. Subsequent to the 2023 proposal of the eight hallmarks of cardiovascular ageing, ABC released two additional frameworks elucidating cardiovascular senescence. The 2024 Wiggers-Bernard Conference on In Vivo Senescence produced widely endorsed guidelines coauthored by field leaders, whereas the NIH-funded SenNet Consortium concurrently proposed standardized protocols for detecting senescent cells across tissues. Created in BioRender (2025) https://BioRender.com/y4u6ysw

Cardiovascular ageing has been subjected to rigorous scientific investigation for over a century, yielding substantial pathophysiological insights, as summarized in Fig. 1. Early observations by William Osler that “a man is as old as his arteries” underscored the centrality of vascular health in organismal ageing. The mid-20th century established key theories linking ageing to cardiac function and oxidative stress. While the late 20th century identified pivotal mechanisms such as endothelial nitric oxide synthesis. The early 21st century marked a turning point with the discoveries of cellular senescence in plaques, the senescence-associated secretory phenotype, and the development of epigenetic clocks. Since around 2015, the field has accelerated toward translation, exemplified by the advent of senolytics, the launch of human clinical trials, and the systematic definition of cardiovascular ageing hallmarks and biomarkers. Recent breakthroughs in molecular gerontology and targeted anti-ageing therapies have stimulated transformative research into age-related cardiovascular disorders. Therefore, a systematic discussion of the related advances and challenges is urgently needed.

This review synthesizes current knowledge on cardiovascular ageing, emphasizing that its progression is accelerated not merely by time but by a complex network of etiologies and risk factors, which is a comprehensive integration not previously consolidated. A key contribution is the novel stratification of established hallmarks into three interconnected tiers: molecular, cellular, and systemic. This framework clarifies their roles while highlighting their essential crosstalk. Furthermore, the mechanistic links between these ageing hallmarks and diverse age-related diseases are elucidated, providing a foundation for targeted clinical translation. While promising therapeutic strategies are emerging, their potential side effects warrant careful consideration. For instance, senolytic agents that clear senescent cells may carry risks such as impaired tissue repair.^17^ Elucidating the processes of cellular senescence, along with their associated hallmarks and signaling pathways, offers new insights into ageing biology and opens promising avenues for addressing CVD in an integrated, system-level framework. Ultimately, advancing cardiovascular health in ageing populations will require strategies that effectively target core ageing mechanisms while diligently managing treatment-related risks.

## The etiology and risk factors for cardiovascular ageing

**Fig. 2:**
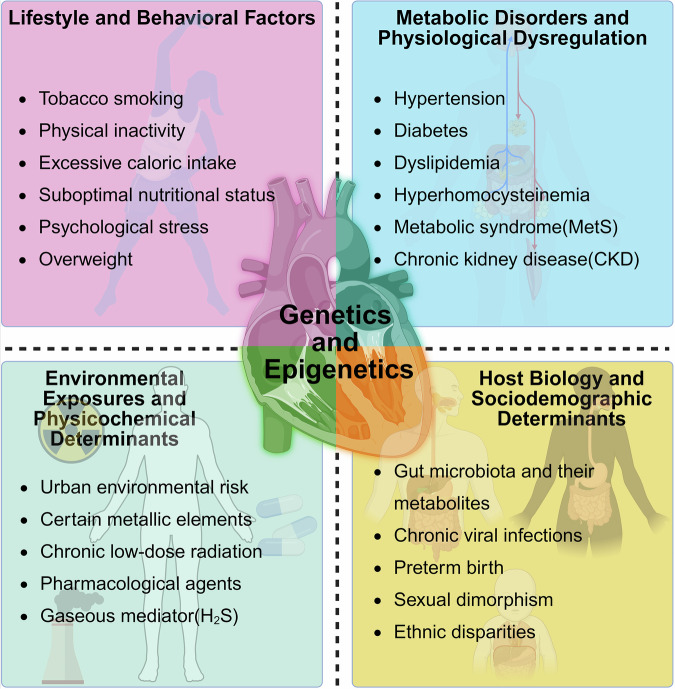
The etiology and risk factors for cardiovascular ageing. The etiological framework of cardiovascular ageing encompasses five principal categories: lifestyle and behavioral factors, metabolic disorders and physiological dysregulation, environmental exposures and physicochemical determinants, genetics and epigenetics, and host biology and sociodemographic determinants. Each category includes multiple specific risk factors. All etiology and risk factors form a highly intricate and interconnected complex network. They may work synergistically or antagonistically, exerting cumulative and sometimes even multiplicative effects. Created in BioRender (2025) https://BioRender.com/1w0a7po

Cardiovascular ageing is a biological process resulting from the progressive accumulation of cellular, tissue, and organ damage over time, leading to functional and structural decline. With increasing age, the accumulation of molecular damage progressively increases. For example, arteries and the myocardium exhibit progressive stiffening and enhanced fibrotic remodeling,^18,19^, whereas the maximal heart rate decreases linearly with age.^4^ Moreover, in addition to chronological ageing itself, specific factors may contribute to or accelerate cardiovascular ageing, as shown in Fig. 2.

### Lifestyle and behavioral factors

Lifestyle and behavioral factors constitute significant contributors to the pathogenesis of cardiovascular ageing. As shown in Fig. 2, chronic exposure to unhealthy lifestyle patterns (including excessive caloric intake, suboptimal nutritional status, physical inactivity, psychological stress, and tobacco use) accelerates the deterioration of cardiovascular function.

Tobacco smoking is a well-established risk factor for accelerated vascular ageing, demonstrating associations with both clinical manifestations of premature cardiovascular senescence and protein-based biomarkers.^20^ Comparative analyses indicate that smokers present significantly greater proportions of senescent and dysfunctional endothelial progenitor cells (EPCs) than nonsmokers do.^21^ Additionally, their endothelial cells show heightened susceptibility to stress-induced premature senescence.^22^

With respect to exercise interventions, long-term physical training has demonstrated substantial efficacy in attenuating age-related cardiovascular functional decline.^23^ Conversely, sedentary behavior in otherwise healthy adults correlates with accelerated cardiovascular ageing trajectories, manifested through increased arterial stiffness, impaired endothelial function, and elevated mortality risk.^24^

Being overweight itself significantly accelerates cardiovascular ageing.^25^ At the dietary level, higher dietary quality indices and adherence to Mediterranean dietary patterns are associated with favorable vascular ageing risk profiles.^26^ Both Mediterranean diets and omega-3-enriched nutritional regimens exert protective effects against vascular ageing.^27,28^ Caloric restriction strategies have the potential for optimizing vascular architecture and function.^29^ Furthermore, lifestyle modifications, including increased physical activity, reduced sedentary behavior, and body weight management, effectively ameliorate vascular ageing phenotypes.^30^

Psychological determinants warrant equal consideration. For example, incarceration-related stress in familial/social networks correlates with validated vascular ageing biomarkers.^31^ Clinical depression elevates coronary artery disease risk,^32^ whereas anxiety disorders are associated with increased cardiac mortality.^33^ Nevertheless, the relationships between psychological factors (including depression, anxiety disorders, and chronic stress) and cardiovascular ageing require further investigation.

### Metabolic disorders and physiological dysregulation

In one cross-sectional study, investigators employed machine learning algorithms to quantify cardiovascular age in 39,559 participants from the UK Biobank, revealing that cardiometabolic risk factors (hypertension, diabetes, and dyslipidemia) collectively accelerate cardiovascular ageing trajectories.^34^ Further observational studies have established causal relationships between vascular ageing and hypertension. Advanced-age populations exhibit hallmark pathological features, including oxidative stress, chronic low-grade inflammation, vascular dysfunction, vasoconstrictive phenotypes, and increased endothelial permeability.^35^

The diabetic microenvironment potentiates vasculature susceptibility to ageing processes,^36^ primarily through dysregulation of the growth hormone/insulin-like growth factor-I (GH/IGF-I) axis and the SIRT1/dimethylarginine dimethylaminohydrolase/asymmetric dimethylarginine (SIRT1/DDAH/ADMA) pathway. These mechanisms systematically impair endothelial cells, vascular smooth muscle cells (VSMCs), and EPCs, thereby driving coordinated vascular senescence.^37,38^ Hyperglycemia exacerbates endothelial ageing through apoptosis signal-regulating kinase 1 (ASK1) signaling pathway activation and the upregulation of plasminogen activator inhibitor-1 (PAI-1) expression. Consequently, ASK1 represents a novel therapeutic target for preventing diabetic vascular ageing.^36^

Murine models of diet-induced hypercholesterolemia exhibit accelerated cellular senescence and vascular dysfunction signatures, characterized by telomere shortening, elevated p16 and p21 mRNA expression, and diminished proliferative and reparative capacities in endothelial cells, EPCs, and hematopoietic stem cells.^39,40^ Hyperhomocysteinemia emerges as another critical determinant of vascular ageing. In rats, a methionine-rich diet-induced hyperhomocysteinemia elevates VSMC senescence markers (SA-β-galactosidase, p53/p21, p16), augments pulse pressure, and promotes collagen deposition.^41^ This condition additionally inactivates telomerase activity in endothelial cells and EPCs, thereby accelerating their senescence.^42^

Metabolic syndrome (MetS) exacerbates the risk of type 2 diabetes and cardiovascular events. Inflammation-dependent accelerated cardiovascular ageing represents a core pathophysiological feature that increases the cardiovascular risk burden in MetS patients.^43^ The uremic milieu in chronic kidney disease is correlated with premature ageing phenotypes,^44^ which clinically manifest as low-grade inflammation, sarcopenia, osteoporosis, frailty, and disproportionately elevated cardiovascular mortality.^45,46^ Specific uremic toxins (e.g., IL-6, IL-1β, CXCL8, and leptin) are associated with profound VSMC alterations and other pathological changes that drive vascular calcification and early vascular ageing (EVA).^47^ Furthermore, cumulative uremic toxin exposure induces allostatic overload (cumulative physiological wear from chronic stress), thereby accelerating senescence and promoting EVA development.^48^

### Environmental exposures and physicochemical determinants

CVD is the leading cause of morbidity and mortality worldwide, with urban environmental risk factors substantially contributing to the CVD burden. Urbanization exacerbates vascular ageing through increased exposure to air pollutants.^49^ Furthermore, analysis of 3772 participants from the National Health and Nutrition Examination Survey (NHANES, 2005–2016) demonstrated that chronic exposure to metallic elements—including cadmium (Cd), cesium (Cs), cobalt (Co), and lead (Pb)—exhibited significant dose-dependent associations with accelerated vascular ageing biomarkers.^50^ Chronic low-dose radiation exposure induces persistent cardiac proteomic remodeling, which disrupts myocardial energy metabolism, extracellular matrix homeostasis, oxidative stress regulation, and senescence-associated signaling pathways. These molecular perturbations recapitulate hallmarks of age-related cardiac pathology, thereby increasing susceptibility to cardiovascular injury. Notably, bioengineered human cardiac tissues subjected to one-month spaceflight conditions displayed reduced contractile force, arrhythmic beating patterns, and molecular/genetic signatures consistent with accelerated ageing phenotypes.^51^

Pharmacological agents further modulate cardiovascular ageing trajectories.^34^ A 2008 Japanese study revealed that doxorubicin-treated cardiomyocytes acquire morphological and functional characteristics analogous to those of senescent cardiomyocytes in aged rodents, implicating premature cellular senescence as a mechanism underlying anthracycline-induced cardiotoxicity.^52^ Systematic reviews confirm that anthracycline-driven cardiac ageing underlies persistent chemotherapy-associated cardiovascular complications, particularly progressive myocardial dysfunction.^53^ Cross-sectional analyses identify opioid dependence as an independent accelerator of arterial stiffness and vascular senescence, with stronger dose‒response correlations in female cohorts than in male cohorts.^54^ Longitudinal evidence further links chronic opioid use to elevated vascular stiffness and senescence biomarker profiles, indicative of accelerated systemic biological ageing.^55^ Human immunodeficiency virus (HIV) management studies have demonstrated comparable vascular ageing indices (e.g., Framingham risk scores) between early antiretroviral therapy initiation and chronic infection groups at the six-year follow-up. Paradoxically, higher CD4^+^ T-cell counts are correlated with poorer vascular health metrics.^56^

Emerging research highlights hydrogen sulfide (H_2_S) as a gaseous mediator that influences senescence-associated molecular mechanisms, positioning H2S as a promising therapeutic target for mitigating age-related cardiovascular pathologies.^57^

### Genetics and epigenetics

Human ageing is strongly associated with an increased risk of CVD. Cardiac ageing studies in Drosophila have identified critical gene networks governing cardiac senescence, involving nutrient-sensing pathways, ion channel regulation, and sarcomere-related genes. These evolutionarily conserved mechanisms operate similarly in mammalian cardiac ageing.^58^ UK Biobank analyses demonstrated that cardiovascular ageing is significantly associated with common/rare variants in genes regulating sarcomeric homeostasis, myocardial immune modulation, and tissue responses to biophysical stress.^34^ Artificial intelligence-driven electrocardiogram analysis for cardiovascular age prediction reveals that delta age (the difference between predicted and chronological age) is associated with all-cause mortality and comorbidities. Genome-wide association studies have further revealed that its genetic architecture predominantly involves cardiovascular system-related genes. Senescence- and longevity-associated genes modulate cardiovascular function through downstream pathways involving inflammatory cascades and oxidative stress, representing therapeutic targets for preventing age-related cardiovascular dysfunction.^59^ Neuregulin-1 (NRG-1), an epidermal growth factor with cardioprotective and antiatherogenic properties, significantly suppresses stress-induced premature senescence in diabetic murine aortae in in vitro and in vivo models. Conversely, ErbB4 receptor deficiency induces cellular senescence across experimental systems.^60^ Klotho may exert cardioprotective effects against ageing through autophagy activation and apoptosis suppression.^61^

The cardiovascular system critically affects physiological health. Age-related epigenetic modifications influence gene expression patterns, cellular differentiation, and disease pathogenesis. Biological age, which is distinct from chronological age, reflects systemic health status. Accelerated vascular ageing lowers disease thresholds, whereas modifiable lifestyle factors (nutrition, exercise) interact with epigenetic clocks to synergistically increase cardiovascular health, suggesting that epigenetic interventions are viable strategies for achieving healthy ageing.^62^ Smooth muscle cell-mineralocorticoid receptor (SMC-MR) signaling promotes vascular stiffness and senescence through EZH2-mediated H3K27me3 epigenetic regulation.^63^ Noncoding RNAs (ncRNAs) serve as master regulators of ageing-associated biological processes,^64^ with established roles in cardiovascular pathophysiology.^65^ Among these, microRNAs (miRNAs) represent the most extensively characterized class, modulating cardiovascular function through cell differentiation, proliferation, apoptosis, angiogenesis, and contractility regulation.^1^ For example, miR-34a overexpression in aged hearts promotes cardiomyocyte apoptosis and fibrosis by targeting PNUTS, contributing to functional decline. Pharmacological inhibition of miR-34a attenuates age-related cardiomyocyte death and postinfarction fibrosis, enhancing functional recovery.^66^ Long noncoding RNAs (lncRNAs), although less evolutionarily conserved than miRNAs, play emerging roles in cardiac ageing through senescence pathway modulation.^67^ Circular RNAs, first identified in the early 1990s, are increasingly implicated in cardiovascular cellular senescence and ageing-related pathologies. However, their mechanistic roles remain incompletely understood.^68^

### Host biology and sociodemographic determinants

The role of the gut microbiota and its metabolites in cardiovascular pathologies is gaining increasing recognition. A Spanish cross-sectional study revealed significant associations between EVA and an elevated abundance of *Bilophila* in the gut microbiome.^69^ The gut microbial composition may mitigate age-related diseases through systemic immune modulation and increased infection resistance.^70^ Compared with noncentenarians, centenarians exhibit distinct gut microbiota profiles,^71^ with emerging evidence suggesting that altered gut microbiome composition or function contributes to age-dependent vascular dysfunction and cardiovascular pathogenesis.^72^ The gut microbiota critically regulates host metabolism, immune responses, and inflammatory pathways—all of which are mechanistically linked to vascular ageing processes.^73–76^

Virus-associated progeroid phenotypes have been observed in patients with chronic viral infections, including HIV, hepatitis B/C/D viruses (HBV/HCV/HDV), Epstein–Barr virus, human herpesviruses, human papillomavirus, and SARS-CoV-2.^77–79^ HIV-infected individuals exhibit premature senescence in stem cells, endothelial cells, and leukocytes. SARS-CoV-2 infection accelerates endothelial senescence and induces the release of the senescence-associated secretory phenotype (SASP).^78^ Centenarians typically exhibit immune profiles optimized for controlling low-grade inflammation while maintaining antimicrobial defense capacity. Antimicrobial peptides (AMPs) may confer protection by directly neutralizing pathogens and preventing infections that could otherwise accelerate senescence or trigger systemic inflammation. This resilience is partially attributed to their unique immune signatures^80^ and preserved adaptive immunity.^81^

Preterm birth is correlated with myocardial remodeling and accelerated cardiovascular ageing later in life. Echocardiographic analyses revealed abnormal left ventricular function in very preterm infants at six months post-natally.^82^

Sex-specific cardiovascular ageing trajectories emerge early in life. Age-dependent sexual dimorphism in vascular structure/function contributes to differential coronary artery disease manifestations. Hormonal and nonhormonal factors jointly mediate sex disparities in cardiovascular ageing and age-related disease progression.^83^ Females generally exhibit lower CVD susceptibility and more robust immune responses,^84^ whereas males exhibit earlier vascular ageing phenotypes. However, these sex differences attenuate in elderly populations,^85^ potentially reflecting menopausal transitions in middle-aged females.^86^ A study revealed that elevated prolactin levels in healthy postmenopausal women are associated with endothelial dysfunction and blood pressure alterations, particularly in the prediction of accelerated arterial stiffening in younger cohorts.^87^

A cross-sectional study of Gujarati Asian Indians revealed significantly greater vascular versus chronological age (6.54 ± 9.5 years), indicating premature vascular ageing in this population. The key risk factors included hypertension, dyslipidemia, and tobacco use.^88^ An ongoing clinical trial further demonstrated that sub-Saharan Black individuals exhibit worse cardiovascular risk profiles and vascular ageing indices despite being younger,^89^ highlighting ethnic disparities in cardiovascular ageing trajectories.

## Pathophysiological changes associated with cardiovascular ageing

### Heart ageing

**Fig. 3:**
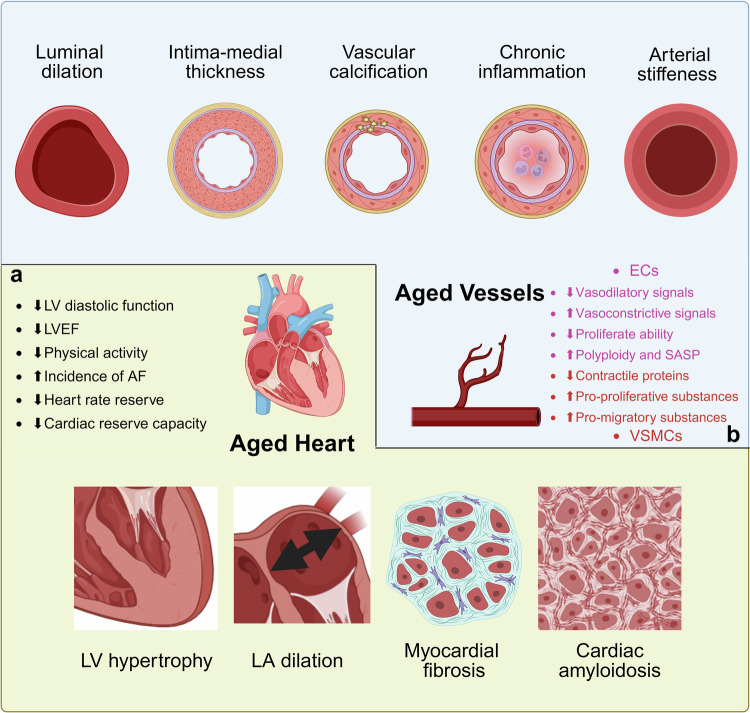
Changes in cardiovascular ageing. **a** Aged hearts undergo myocardial fibrosis, cardiac amyloidosis, LA dilation and LV hypertrophy. These structural changes lead to functional impairments, such as decreases in LV diastolic function and LVEF. These impairments reduce the heart’s capacity to respond to disease or injury, eventually contributing to an increased incidence of CVD, such as AF. **b** Aged vessels undergo vascular dyshomeostasis and remodeling, such as luminal dilation, intima‒medial thickness, chronic inflammation, vascular calcification, arterial stiffening and decreased elasticity. A range of changes in senescent ECs and VSMCs exacerbate the onset and outcome of vascular dysfunction, which is intrinsically linked to vascular ageing and related diseases. *Abbreviations****:*** *LV, left ventricle; LA, left atrium; LVEF, left ventricular ejection fraction; AF, atrial fibrillation; ECs, endothelial cells; VSMCs, vascular smooth muscle cells; SASP, senescence-associated secretory phenotype. Created in BioRender (2025)* https://BioRender.com/2x6apnt

As depicted in Fig. 3a, as the heart ages, it undergoes structural changes, such as myocardial fibrosis^5^ and cardiac amyloidosis,^18,19^ which increase its functional demands. Concurrently, left atrial (LA) dilation and left ventricular (LV) hypertrophy occur.^6^ The compensatory mechanisms developed by the heart at rest to accommodate these changes often lead to functional impairments. Progressive deterioration of LV diastolic function^90^ is accompanied by reduced left ventricular ejection fraction (LVEF),^6^ collectively indicating diminished cardiac pumping capacity. Concurrently, systemic physical activity levels decrease,^6^ potentially reflecting compromised cardiac functional status. Both heart rate reserve^4^ and cardiac reserve capacity exhibit age-dependent reductions, impairing the compensatory responses of the heart to external stimuli and increasing hemodynamic stress. Furthermore, intracardiac electrophysiological alterations emerge during ageing.^91^ These alterations collectively contribute to an ageing cardiac phenotype characterized by progressive functional deterioration, impaired rhythm regulation, and diminished adaptive capacity. ageing is also associated with progressive panmyocardial impairment of coronary vasodilatory capacity due to an increase in minimal microvascular resistance.^92^ These impairments reduce the heart’s capacity to respond to disease or injury, eventually contributing to an increased incidence of CVD, such as atrial fibrillation.^10^

In addition, the coronary microvasculature undergoes significant structural remodeling, characterized by increased vascular stiffness, progressive thickening of the intimal layer,^6^ and reduced capillary density.^93^ Structural microcirculatory remodeling is reflected in backward expansion wave intensity and diastolic microvascular conductance measurements.^94^ Functionally, these structural alterations contribute to endothelial dysfunction,^10^ which is characterized by diminished nitric oxide (NO) bioavailability and heightened oxidative stress, disrupting the balance of vasoactive signaling.^95^ Simultaneously, dysregulation of vascular tone emerges, alongside a lowered threshold for cellular Ca^2+^ overload that exacerbates ischemic injury.^10^ These changes collectively lead to tissue perfusion deficiency, compromising oxygen and nutrient delivery to cardiomyocytes. Furthermore, age-related increases in vascular permeability and lymphatic dysfunction^96^ and reductions in baseline coronary blood flow and coronary reserve capacity, such as coronary flow reserve (CFR), further impair myocardial perfusion.^97^

Heart ageing is associated with widespread deterioration of both the structure and function of the microvasculature, which accelerates cardiac remodeling and ultimately disrupts the coupling between the microcirculation and the heart (Fig. 3a), fostering the development and progression of CVD.^6^ This deterioration extends beyond the heart, affecting other critical organs, such as the brain, kidneys, skeletal muscle, and eyes, thereby accelerating the ageing process. This systemic decline disrupts tissue oxygenation, waste elimination, and the efficient transport and distribution of nutrients, hormones, growth factors, and metabolites throughout the body, significantly increasing the risk of chronic diseases.^98^

### Vascular ageing

As a famous 19th-century doctor, William Osler proposed, “A man is as old as his arteries”. Vascular ageing is considered the most important risk factor for high CVD mortality. It is a progressive process that involves structural and functional changes in the vasculature during the ageing process and is characterized mainly by vascular cell dysfunction and senescence, vascular dyshomeostasis and remodeling (Fig. 3b), such as luminal dilation, intima‒medial thickness, chronic inflammation, vascular calcification, arterial stiffening and decreased elasticity.^8,9^

The arterial wall is composed of three anatomical layers: a single layer of endothelial cells (the tunica intima), multiple layers of vascular smooth muscle cells and elastic fiber layers between VSMC layers (the tunica media), and the tunica adventitia, which contains adipocytes, fibrous connective tissue, and ECM. ECs and VSMCs, which form the intima and media layers of the vessel wall, respectively, are closely associated with normal vascular function. The accumulation of senescent ECs and VSMCs in the culprit lesions of the cardiovascular system exacerbates the onset and outcome of vascular dysfunction, which is intrinsically linked to vascular ageing and related diseases.

For example, senescent ECs lose their balance in the production of vasodilatory and vasoconstrictive agents, characterized by decreased vasodilatory signals and increased vasoconstrictive signals. This reduced endothelium-dependent vasodilation is consistently observed in the ageing vascular wall of human and animal models.^7^ Furthermore, senescent ECs undergo phenotypic changes (Fig. 3b) that alter the pattern of expressed proteins, lose their ability to proliferate, become flattened and enlarged in shape and size, exhibit increased polyploidy and SASP, and are accompanied by increased oxidative stress and a decrease in proteostasis.^99^ In addition, endothelial senescence reduces vascular density, increases intima‒media layer thickness and collagen deposition, reduces elastin deposition, and induces vascular lumen dilation, which occurs in multiple organ systems. More importantly, endothelial barrier function is disrupted, facilitating the circulation of harmful substances that affect VSMCs in the tunica media. Emerging evidence has shown that the quantity of VSMCs in the tunica media decreases in ageing blood vessels, and a number of VSMCs show features of phenotype switching that involve a decrease in the expression of contractile proteins and an increase in the expression of pro-proliferative and migratory substances, contributing to increased aortic stiffness and calcification.^100^ Furthermore, the metabolism of VSMCs is also correlated with phenotype switching and the progression of vascular ageing. Recent research has further demonstrated that Sox9 promotes compositional and structural ECM changes that regulate VSMC senescence and mimic features of vascular ageing. Healthy VSMCs exposed to Sox9-modified ECM exhibit accelerated senescence, whereas senescent cells are partially rejuvenated when exposed to Sox9-depleted ECM.^101^ Cell senescence has received potential attention as a promising target for preventing vascular ageing and related diseases.

## The hallmarks of cardiovascular ageing

### Molecular level

#### Genomic instability and epigenetic alterations

**Fig. 4:**
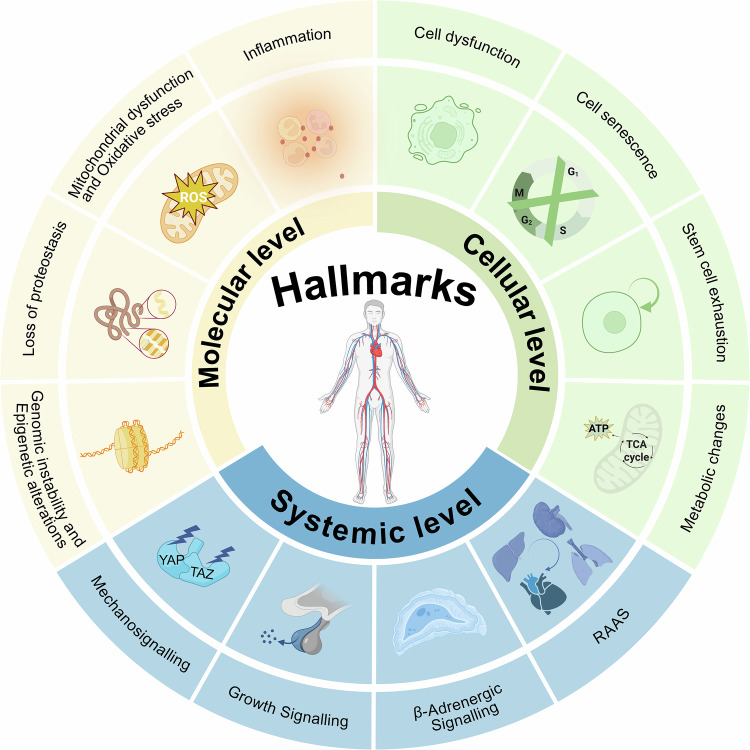
The hallmarks of cardiovascular ageing. This figure categorizes the hallmarks of cardiovascular ageing into three interconnected tiers: the molecular level, encompassing genomic instability and epigenetic alterations, loss of proteostasis, mitochondrial dysfunction and oxidative stress, and inflammation; the cellular level, comprising cellular dysfunction, cellular senescence, stem cell exhaustion, and metabolic changes; and the systemic level, involving signaling pathways, including the renin‒angiotensin‒aldosterone system (RAAS), β-adrenergic signaling, growth signaling, and mechanosignaling. *Created in BioRender (2025)* https://BioRender.com/zasg1s1

Genomic instability is a critical pathogenic factor in vascular ageing (Fig. 4). Studies in mice have demonstrated that local endothelial genomic instability can recapitulate key features of vascular ageing, such as increased vascular stiffness, vascular hypertrophic remodeling, loss of endothelium-dependent vasodilation, increased vascular leakage, and differential vulnerability of various arteries.^102^ These characteristics are also observed in humans.^103,104^ The accumulation of damaged DNA is recognized as one of the primary drivers of ageing and involves multiple processes.^105,106^ Cells with unrepaired DNA damage may either undergo apoptosis or enter senescence. Apoptosis leads to cellular or tissue atrophy and a decline in organ function, whereas senescent cells adopt a SASP, which impacts neighboring cells and induces age-related phenotypic changes.^107^ Additionally, the continuous accumulation of DNA damage can activate a “survival response”, shifting the physiology of organisms from growth promotion to maintaining cellular homeostasis and function.^108^ In endothelial cells, selective defects in DNA repair can result in age-related endothelial dysfunction, primarily due to the reduced bioavailability of endothelial-derived NO. Increased production of superoxide radicals can further impair the perfusion of vital organs.^102^ In humans, genetic and environmental variability contributes to differences in ageing rates among individuals and even among different organs within the same individual. This variability has also been observed in mouse models of premature ageing phenotypes due to defects in DNA repair.^108^ A growing body of evidence suggests that the DNA damage response plays a central role in vascular ageing,^109^ where ageing can be driven by genomic instability and persistent DNA damage in a cell-autonomous manner across various organs and tissues.^110–113^ In addition, DNA damage at the ends of chromosomes (telomeres) contributes to ageing and age-related diseases. DNA damage at telomeres is a recognized contributor to ageing and age-related diseases.^114^ Furthermore, radiation studies in humans and mice have demonstrated that such damage accelerates ageing within the carotid and cerebral vasculature, implicating telomere attrition and cellular senescence as key underlying mechanisms.^103,104,115^

In addition to DNA mutations, gene expression levels in the cardiovascular system are modulated by a variety of epigenetic factors, including DNA methylation, histone modifications, and ncRNAs.^116^ In failing human hearts, alterations in DNA methylation patterns have been identified,^117^ and these patterns are increasingly being utilized as markers to estimate the rate of cardiovascular ageing.^118,119^ However, further research is needed to elucidate the significance and mechanisms of reduced DNA methylation in vascular ageing. Additionally, histone loss and tissue-specific changes in posttranslational histone modifications, such as those involving the SIRT family, are closely associated with ageing. Sirtuins (SIRT1--7), a family of NAD-dependent histone deacetylases, are of particular interest. Since NAD levels decrease with age, NAD supplementation is considered a promising strategy for reversing age-related phenotypes,^120^ despite its uneven distribution in cells as well as subcells.^121^ We have established that SIRTs are activated by NAD^122^ and play important roles in ageing and capillarization.^123^ For example, endothelial cell (EC)-specific Sirt1 knockout mice exhibit reduced cardiac capillarization and diastolic dysfunction,^124^ whereas EC-specific SIRT3 depletion plays the same role.^125^ Notably, the SIRT1 activator resveratrol has broad biological activities that ameliorate the aforementioned ageing-related deficits, establishing this compound as a promising antiageing therapeutic.^126^ Angiotensin II-induced fibrosis reduces vascular density in the heart, and this effect is exacerbated by SIRT3 depletion. Our research also revealed that SIRT2 deficiency exacerbates age-related arterial stiffness and systolic-diastolic dysfunction, accompanied by aortic remodeling. SIRT2 can repress p66Shc to reduce mROS generation. Either p66Shc silencing or MnTBAP treatment could block the inhibitory effects of SIRT2 deficiency on the expression of MMP2 and MMP9, which contribute to vascular remodeling and dysfunction.^127^ The role of other SIRTs in cardiac capillarization remains to be fully explored. Among ncRNAs, circular RNAs, miRNAs, and lncRNAs have emerged as significant epigenetic regulators with roles in cardiovascular ageing.^65^ For example, the lncRNA Sarrah is downregulated in aged mouse hearts and in rodent models of ischemic cardiomyopathy and heart failure with preserved ejection fraction (HFpEF). In contrast, the overexpression of Sarrah enhances cardiomyocyte survival, promotes endothelial cell proliferation, and improves functional recovery from myocardial ischemia while reducing cardiac apoptosis in aged mice.^67^ However, most studies to date have focused on miRNAs, and the role of other ncRNAs remains controversial, with ongoing debates about the extent to which they may arise from transcriptional noise, necessitating further research.^128^

#### Loss of proteostasis

Proteostasis, defined as the balance between protein synthesis, folding, and degradation, is critical for preserving cellular health and enabling cells to respond effectively to stress.^129,130^ Disruptions in proteostasis prompt compensatory cellular adaptations. Cells have developed several mechanisms to minimize protein misfolding and eliminate misfolded proteins,^129^ one of which involves molecular chaperones. These chaperones bind to incomplete polypeptide chains, preventing premature folding and facilitating correct protein folding. Chaperones also mitigate protein denaturation during cellular stress, such as heat shock, which is why they are commonly referred to as heat shock proteins (HSPs).^131–133^ When chaperones and other molecular helpers cannot refold dysfunctional proteins, these proteins are marked for degradation via the ubiquitin‒proteasome system or through chaperone-mediated autophagy (CMA).^134^ As organisms age, the proteostasis network becomes less efficient, resulting in the accumulation of misfolded and damaged proteins, which can aggregate into intracellular inclusions or extracellular amyloid plaques.^129,135^

While the accumulation of toxic protein aggregates is most prominent in neurodegenerative diseases, it is also a characteristic of various age-related CVDs, including atherosclerosis, AF, heart failure, and hypertrophic, ischemic, and dilated cardiomyopathies^136–139^ (Fig. 4). In the vascular system, CMA activity significantly decreases with age.^140^ In lysosome-associated membrane protein type 2A (LAMP-2A)-null mice, the inhibition of CMA accelerates atherosclerotic plaque formation by promoting dyslipidemia, vascular smooth muscle cell dedifferentiation, and proinflammatory macrophage activation. In contrast, systemic activation of CMA in mice overexpressing human LAMP-2A attenuates atherosclerosis severity and slows its progression when induced by a proatherosclerotic diet.^141^ Data from the single-cell human transcriptome atlas (Tabula sapiens) indicate that the expression of CMA-related genes decreases with age, especially in aortic macrophages and smooth muscle cells.^142^ In addition, caloric restriction exerts beneficial effects on cardiovascular ageing by reinforcing proteostasis.^143^ Experimental studies have demonstrated this mechanism through two distinct manifestations: the upregulation of HSP70 in the rodent myocardium^144^ and the activation of protein folding-related transcripts in human skeletal muscle.^145^

Experimental evidence strongly supports the causal role of proteostasis dysregulation in cardiovascular ageing. Studies have shown that inducing (or preventing) the breakdown of proteostasis within the cardiovascular system accelerates (or delays) cardiovascular ageing.^141,146–149^ Thus, the loss of proteostasis is closely linked to age-associated declines in cardiovascular function, suggesting that genetic, dietary or pharmacological strategies to maintain proteostasis may hold promise for delaying cardiovascular ageing.

#### Mitochondrial dysfunction and oxidative stress

Oxidative stress is recognized as a biological process of ageing,^95^ and its effects on age-related cardiovascular dysfunction may be more pronounced than those of other established mechanisms^150^ (Fig. 4). There is growing evidence that mitochondria serve as the central organelles linking vascular ageing and oROSxidative stress, driving interest in the pathways that regulate mitochondrial reactive oxygen species (ROS), which may influence the progression of ageing.^151^ Among these regulators, the p66Shc protein and its transcriptional regulator, deacetylase SIRT1, have received substantial attention in the last decade.^152–155^ Mitochondria are essential for energy metabolism in eukaryotic cells, and through the coordinated action of the respiratory chain, they produce the necessary energy substrates. As a result, age-related deterioration in energy metabolism is closely tied to specific changes in mitochondrial function and dynamics. Impaired electron transport within the mitochondrial respiratory chain leads to increased ROS production and reduced ATP synthesis, which is considered a key driver of the ageing process.^156,157^ Vascular endothelial cells largely rely on glycolysis, yet pharmacological inhibition of mitochondrial respiratory chain complexes disrupts ex vivo vascular tone control.^158,159^ Notably, altered mitochondrial redox status has been mechanistically linked to impaired coronary arteriolar flow regulation, suggesting that targeted restoration of mitochondrial bioenergetics may improve clinical indices of coronary function, including the CFR and FFR.^160^ Furthermore, mitochondrial dysfunction is also linked to disrupted calcium signaling due to alterations in the type 2 ryanodine receptor (RyR2) and the sarcoplasmic reticulum Ca^2+^ ATPase pump.^161^

Cardiovascular ageing promotes oxidative stress alongside chronic inflammation. NADPH oxidase, a major source of ROS, is upregulated during vascular ageing, intensifying oxidative stress and activating proinflammatory pathways, particularly the NF-κB pathway.^162^ Excess ROS not only act as common damaging agents in various inflammatory pathways but also contribute to chronic, low-grade inflammation, which is characteristic of ageing. The most significant impact of ROS on endothelial dysfunction stems from the degradation of endothelial-derived NO. Reduced endothelial NO synthase (eNOS) activity leads to lower NO bioavailability,^163^ a hallmark of age-related endothelial dysfunction, which results in vasoconstriction and impaired tissue perfusion.^109^ This, in turn, contributes to cardiovascular remodeling and triggers adaptive metabolic changes in peripheral tissues. Reduced NO levels may also impair cardiomyocyte contractility, leading to decreased LV diastolic compliance via a PKG-dependent pathway.^164^

In conclusion, the cardiovascular system is highly reliant on mitochondrial metabolism and signaling, and mitochondrial dysfunction due to ageing has a particularly profound impact on cardiovascular function,^165^ making it a central pathogenic factor in many CVDs.^166,167^

#### Inflammation

A chronic proinflammatory state is a hallmark of ageing (Fig. 4). This persistent, low-grade inflammation, which occurs without an obvious infection, is termed “inflammageing” ^168^ and is a major risk factor for morbidity and mortality in the elderly.^169^ Potential mechanisms of “inflammageing” include genetic predisposition, central obesity, increased gut permeability, changes in the microbiome, cellular senescence, activation of the NLRP3 inflammasome, oxidative stress due to mitochondrial dysfunction, chronic infections, and immune cell dysregulation.^170^ Circulating inflammatory biomarkers can predict the progression of atherosclerosis and its cardiovascular complications, whereas inflammatory processes further drive secondary events that indicate disease progression. Inflammation also plays a crucial role in disrupting cardiovascular homeostasis.^171^ Dysregulation of immune‒inflammatory pathways during ageing results in elevated levels of circulating cytokines, including tumor necrosis factor-α (TNF-α), interleukin-1 (IL-1), and interleukin-6 (IL-6), which are located primarily in the “central pathway“.^172,173^ Studies have demonstrated that interventions aimed at the pathogenic pathway effectively attenuate chronic inflammatory states, consequently slowing the progression of cardiovascular ageing.^174,175^ Moreover, our research has shown that the chemokine CCL17 can regulate the reprogramming of Th cells in the immune microenvironment, acting as a coordinator of vascular ageing. Targeting CCL17 represents a promising new anti-inflammatory approach to prevent cardiovascular ageing. Cook et al.^176^ reported that IL-11 plays a central role in fibrosis across the cardiovascular system and multiple organs and that inhibiting IL-11 may extend lifespan. Additionally, the persistent low-grade inflammatory environment produces advanced glycation end products (AGEs) and matrix metalloproteinases and, combined with increased vascular permeability, promotes the local accumulation of inflammatory cells.^109^ These inflammatory cells and cytokines not only directly damage the vascular wall but also increase ROS production, further compromising the vascular structure, inducing arteriolar remodeling, and triggering compensatory responses such as angiogenesis.^177^ These compensatory mechanisms aim to restore or maintain cardiovascular balance but place sustained stress on endothelial cells, leading to their premature senescence. In turn, senescent immune-inflammatory cells acquire a proinflammatory phenotype when dysfunctional, perpetuating the inflammatory response and creating a vicious cycle.^171^

### Cellular level

#### Cell dysfunction

This review examines cell dysfunction through two distinct dimensions: the specific functional impairments affecting cells and the particular cell types manifesting these dysfunctions. Specifically, it focuses on macroautophagy impairment and immune cell dysfunction, both of which contribute significantly to cardiovascular ageing (Fig. 4).

Macroautophagy serves as a critical cellular quality control mechanism responsible for degrading and recycling dysfunctional cytoplasmic components. This process begins with the encapsulation of toxic protein aggregates and aged organelles within double-membraned vesicles called autophagosomes. These vesicles subsequently merge with lysosomes, where the enzymatic breakdown of their contents generates metabolites essential for energy production and biosynthesis.^178^ In cardiomyocytes, macroautophagy extends to a specialized process termed heterophagy, wherein damaged mitochondria are packaged into extracellular vesicles (exospheres) and expelled into the extracellular space. Here, cardiac macrophages degrade cellular debris, further underscoring the role of autophagy in maintaining cellular homeostasis.^179,180^ Owing to its vital contributions to cellular longevity, macroautophagy is a key determinant of healthspan and lifespan across eukaryotic cells, particularly within the cardiovascular system.^170,181,182^ However, autophagy efficiency decreases with organismal ageing,^183–185^ a phenomenon linked to the onset of age-related chronic diseases. In the cardiovascular context, studies in aged mice and humans have revealed diminished autophagic activity in vascular tissues.^186^ Advanced methodologies to assess autophagic flux in model organisms (e.g., flies and rodents) have conclusively demonstrated age-dependent impairment in autophagy within both the heart and vasculature.^187–189^ Such deficits—observed in cardiomyocytes, VSMCs and ECs—result in structural and functional abnormalities characteristic of cardiovascular ageing.^190^ The age-related decline in autophagy arises through multiple interconnected mechanisms. Central to this process is the hyperacetylation of autophagy-related proteins, a consequence of reduced SIRT deacetylase activity and decreased NAD^+^ levels in the ageing cardiovascular system.^191,192^ Nutrient excess—particularly prevalent in visceral obesity and metabolic syndrome—further suppresses autophagy by increasing acetyl-CoA availability (promoting protein acetylation) and hyperactivating the insulin-IGF1-mTOR (mammalian target of rapamycin) signaling axis. Calcium signaling, which is altered during cardiovascular ageing,^193^ also impairs autophagic flux, especially in ECs and cardiomyocytes.^194,195^ Additionally, diminished levels of the polyamine spermidine^196,197^ disrupt the hypusination-dependent synthesis of autophagy machinery components.^198^ Human studies corroborate this link, demonstrating a progressive age-dependent decline in cardiac spermidine. Conversely, spermidine supplementation in aged mice restores autophagy in both cardiac and vascular tissues,^199,200^ highlighting its therapeutic potential.

Immune cells play a pivotal role in cardiovascular ageing, with their functional dysregulation driving and amplifying age-related pathologies.^201–203^ Emerging evidence suggests that macrophages are critical modulators of cardiac senescence.^204^ In addition, CD28^−^ T cells play a pivotal role in age-related cardiovascular diseases.^205^ CD57^+^ T cells exhibit reduced proliferative capacity and increased secretion of proinflammatory cytokines during ageing. In humans, both CD28^-^ and CD57^+^ T cells are considered replicatively senescent T-cell subsets, and their frequencies in peripheral blood increase with increasing age.^206^ However, key knowledge gaps persist. The functional trajectories of immune cells (e.g., shifts in proinflammatory cytokine secretion, phagocytic capacity, and antigen presentation) during cardiovascular ageing remain poorly characterized. Delineating how age-altered immune crosstalk exacerbates endothelial dysfunction, myocardial fibrosis, and vascular stiffness will be essential for developing immunomodulatory therapies to decelerate cardiovascular decline.

#### Cell senescence

Cellular senescence is typically defined as a state of permanent cell cycle arrest characterized by the irreversible loss of replicative capacity. It results in the loss of specific cellular functions and the acquisition of a proinflammatory secretory and metabolic phenotype in both cardiomyocytes and nonmyocyte cardiac cells.^207,208^ In postmitotic cardiomyocytes, chronic stressors such as oxidative damage and inflammation drive telomere attrition, DNA damage accumulation, functional decline, and polyploidization—hallmark features of cellular senescence.^209–211^ These senescent cardiomyocytes exhibit impaired contractility, cellular hypertrophy, mitochondrial dysfunction, and shortened telomeres, all of which collectively undermine myocardial performance.^212,213^ The age-dependent accumulation of these dysfunctional cells disrupts intercellular communication, impairing tissue homeostasis and propagating chronic inflammation through the SASP, ultimately culminating in cardiomyocyte loss.^212,213^ Thus, targeting cardiomyocyte senescence has emerged as a central therapeutic strategy to mitigate age-related structural and functional cardiac deterioration.^214–216^ Concurrently, vascular and interstitial cardiac cells (e.g., fibroblasts and progenitor cells) undergo senescence characterized by cell cycle arrest, heightened inflammatory signaling, and the upregulation of senescence markers (e.g., p16INK4A).^217^ Senescent endothelial cells produce an increased number of functional small extracellular vesicles (EVs), which may play a role in vascular physiology and disease.^218^ VSMCs are the principal cell type in the vascular wall and maintain vascular tone. EVA-related phenotypic switching of VSMCs contributes to cardiovascular ageing.^219^ Senescent cardiac fibroblasts exacerbate maladaptive remodeling in aged hearts by secreting matrix-degrading enzymes and profibrotic factors.^217^ Age-related telomere shortening and dysregulated cell division also impair the self-renewal capacity of cardiac progenitor cells (CPCs), further accelerating global cardiac ageing.^220–222^ Notably, hematopoietic stem cells with telomerase deficiency exhibit a reduced replicative lifespan during serial transplantation,^223^ whereas bone marrow-derived monocytes from ischemic heart disease patients display telomere erosion, impaired differentiation, and elevated p21/p16INK4A expression.^224^ These observations underscore a critical barrier in regenerative medicine: autologous stem/progenitor cell therapies often rely on aged, functionally compromised cells from CVD patients. Reprogramming aged somatic cells into induced pluripotent stem cells (iPSCs) offers molecular insights into rejuvenation mechanisms, although translational challenges persist for cardiac applications. Overall, restoring functional competence in aged stem cells represents a pivotal bottleneck for developing effective personalized regenerative therapies.

Historically, the most prominent feature of cellular senescence has been stable proliferative arrest, which is mediated by the activation of the tumor suppressors TP53 and CDKN2A/p16, along with their downstream effectors CDKN1A/p21 and retinoblastoma-1 family proteins. Together, these proteins inhibit cell cycle progression by suppressing cyclin-dependent kinases (CDKs) and E2F family transcription factors.^225^

In mouse models of pressure overload, increased p53 expression, which induces cellular senescence, exacerbates cardiac dysfunction. p53 promotes cardiac hypoxia and remodeling by inhibiting hypoxia-inducible factor 1α (HIF-1α) and VEGF, thereby suppressing cardiac angiogenesis.^226^ Increased p53 signaling in endothelial cells leads to capillary rarefaction in cardiac tissue, whereas depletion of endothelial p53 improves capillary density and cardiac function and suppresses cardiac fibrosis.^227^ Furthermore, p53 activation in cardiac endothelial cells and bone marrow cells promotes cardiac inflammation by increasing the expression of adhesion molecules to attract inflammatory cells. Deletion of the p53 gene downregulates these molecules, suppresses inflammation, and improves cardiac function. Conversely, the overexpression of p53 in bone marrow cells worsens cardiac inflammation and dysfunction.^228^

The protein p21, encoded by the cyclin-dependent kinase inhibitor 1a (Cdkn1a) gene, is a cell cycle regulator downstream of p53.^229^ The activation of p21 triggers cellular senescence and apoptosis.^230^ Compared with 4-month-old mice, 24-month-old C57BL/6 mice presented higher levels of p21 in cardiac tissue.^231^ Additionally, systemic depletion of p21 reduces capillary density and impairs cardiac contractile function.^232^ The role of p21 in capillary formation may be context dependent, varying across organs, cell types, and diseases. p16INK4a, a cell cycle regulator encoded by Cdkn2a, is widely used as a marker of senescent cells and has been reported to increase with age in cardiomyocytes,^207^ endothelial cells,^233^ and cardiac progenitor cells.^234^ The levels of p16 increase in the hearts of aged mice.^231^ In humans, protein levels of p53, p21, and p16 increase in the endothelial cells of older individuals (~60 years old), whereas these levels are reduced in physically active older adults (~57 years old).^233^ The functional diversity of p16^Ink4a+^ senescent cells has led researchers to develop a genetic toolset comprising three p16Ink4a-related systems for tracking, ablating, and manipulating p16^Ink4a+^ cells in vivo. This toolset lays the foundation for developing future cell type-specific senolytic therapies.^235^ However, further studies are needed to elucidate the role of p16 in cardiac capillarization and explore more specific senescence markers.

#### Stem cell exhaustion

Cardiomyocytes undergo renewal with age, with the highest renewal rate occurring during the first two decades of life. At the age of 20, the cardiomyocyte renewal rate is ~1% per year, but this rate decreases to less than 0.5% per year with increasing age.^236^ In contrast, endothelial cells have a high renewal rate throughout life (greater than 15% annually), whereas mesenchymal stem cells have a more limited renewal rate (less than 4% per year in adulthood).^237^ CD133^+^ endothelial progenitor cells not only restore neovascularization but also improve longevity in progeroid and naturally aged murine models.^238^ Arterial Sca1 vascular stem cells generate functional smooth muscle cells for vascular repair and regeneration.^239^ Stem cell-derived extracellular vesicles mitigate age-related arterial stiffening and hypertension.^240^ Mounting evidence confirms the multifaceted roles of stem cells in vascular repair: restoring endothelial integrity, promoting angiogenesis, and modulating inflammatory pathways to attenuate atherogenesis.^241,242^

Pericytes, which are mesenchymal stem cells associated with capillaries, play a critical role in maintaining vascular homeostasis. They are key regulators of angiogenesis, vascular permeability, barrier function, and extracellular matrix (ECM) formation.^243^ As mechanical support for blood vessels,^244^ pericytes possess regenerative potential.^245^ The evidence also suggests that the pericyte secretome plays an important role in controlling and altering the local cardiac environment. Regulator of G-protein signaling 5 (RGS5) belongs to a family of proteins that act as GTPase-activating proteins, regulating G-protein-coupled receptor (GPCR) signaling pathways by inhibiting the Gq protein alpha subunit (Gαq) and Gi protein alpha subunit (Gαi). In smooth muscle cells, RGS5 has been shown to regulate both proliferation^246^ and contraction.^247^ A decrease in RGS5 expression in pericytes is a marker of cardiac ageing. RGS5 is crucial for maintaining pericyte function and preventing the activation of profibrotic gene expression in the heart. The loss of RGS5 in pericytes drives these mural cells into an entropic state characterized by morphological changes, excessive ECM deposition, and the secretion of harmful profibrotic growth factors in the heart.^248^

Ageing is associated with reduced tissue renewal under homeostatic conditions and impaired tissue repair following injury, with each organ employing its own strategies for renewal and repair.^249^ In fact, tissue repair is thought to rely heavily on injury-induced dedifferentiation and cellular plasticity. Injury-induced plasticity (and its progressive loss with ageing) may be more relevant to ageing than the plasticity of resident stem cells under normal homeostatic conditions. Both stem cells and progenitor cells experience the same ageing hallmarks as cells without stem cell potential.^12^ During embryonic development, very small embryonic-like stem cells, which act as mobile reservoirs of circulating pluripotent stem cells (PSCs), are deposited in adult tissues. However, the number of these cells decreases with age.^250^ Thus, age-dependent exhaustion of the PSC pool in adult tissues may contribute to the ageing process to some extent.

Cellular reprogramming is a commonly used strategy to combat the decline in stem cell function with ageing. Reprogramming toward pluripotency involves the transduction of four external transcription factors—OCT4, SOX2, KLF4, and MYC (collectively known as OSKM)—which convert somatic cells into embryonic-like pluripotent stem cells, referred to as iPSCs.^251^ Short-term cyclic expression of OSKM ameliorated the cellular and physiological hallmarks of ageing and extended lifespan in a premature ageing mouse model. Additionally, in vivo OSKM expression enhances recovery from metabolic disease and muscle injury in aged wild-type mice.^252^ In addition, transient reprogramming restores the regenerative capacity of aged tissues, enabling them to repair injuries as effectively as young individuals do. This enhanced repair capacity has been demonstrated in models of cardiac tissue injury.^253^

#### Metabolic changes

Metabolic alterations within tissues and intercellular metabolite trafficking represent emerging frontiers in cardiovascular ageing research (Fig. 4). Accumulating evidence underscores that metabolic reprogramming is essential in senescent cells to redirect substantial energy toward senescence-specific functions, notably the SASP and the modulation of immune responses within the tissue microenvironment. Despite their diminished proliferative capacity, senescent cells exhibit heightened metabolic activity. A hallmark of this state is a pronounced shift toward glycolysis, persisting under normoxic conditions—a Warburg-like effect analogous to that observed in cancer cells. This bioenergetic reprogramming involves shunting pyruvate, the end-product of glycolysis, away from oxidative phosphorylation. The resulting altered metabolic state may constitute an adaptive response to elevated oxidative stress driven by dysfunctional mitochondrial accumulation.^254^ Recent findings highlight the significance of EC senescence: impaired glucose transport is associated with cardiac EC senescence^255^; a glycolytic shift promotes vascular senescence, an effect reversible by senolytics^256^; and evidence suggests reduced lactate production in senescent ECs. Conversely, studies have reported increased fatty acid oxidation activity and increased intracellular ATP content in senescent human umbilical vein endothelial cells.^257^ Collectively, these findings reveal a complex and sometimes seemingly contradictory metabolic landscape within senescent ECs. Despite unresolved discrepancies, interest in senescent cell metabolism is rapidly increasing, warranting dedicated attention as a key area for elucidating the mechanisms of cardiovascular ageing and identifying therapeutic targets.

### Systemic level

Systemic and local signaling pathway dysregulation, including the renin‒angiotensin‒aldosterone system (RAAS), β-adrenergic signaling, growth signaling, and mechanosignaling, results in chronic activation during ageing, leading to cardiovascular dysfunction (Fig. 4).

#### RAAS

Chronic activation of the RAAS is closely associated with age-related CVD, including hypertension, atherosclerosis, coronary artery disease, AF, and heart failure.^258^ Although the RAAS has adaptive functions in the early stage of CVD, its long-term activation triggers adverse cardiovascular effects through various mechanisms, including disruption of sodium homeostasis; dysregulation of vascular tone and blood volume; and abnormal cell proliferation and growth, oxidative stress, inflammation, and fibrotic remodeling in the cardiovascular system.^259,260^ Studies have shown that circulating levels of renin and angiotensin II increase with age in healthy elderly mice. Additionally, in the hearts and vascular systems of older mice, there is a significant increase in the expression of angiotensin II mRNA and protein.^261^ This process is accompanied by elevated vascular expression of ACE, collagen IV, fibronectin, and transforming growth factor-β (TGF-β), along with increased susceptibility to developing hypertension in response to low doses of angiotensin II.^262^ These findings suggest that overactivation of the RAAS system is a key mechanism driving cardiovascular ageing, independent of common CVD. Additionally, the expression of mineralocorticoid receptors (MRs) in vascular smooth muscle cells gradually increases with age.^263,264^ This enhanced expression leads to vasoconstriction, increased vascular tension, and exacerbated oxidative stress, which in turn results in elevated blood pressure and vascular stiffness.^265^ As transcriptional regulators, smooth muscle cell-MRs promote vascular fibrosis by activating genes related to fibrosis, thereby reducing vascular elasticity and further worsening vascular stiffness.^266,267^ Moreover, MR enhances the expression of target genes, including the L-type calcium channel (LTCC) subunit Cav1.2 and the angiotensin II type 1 receptor (Agtr1), by inhibiting the expression of miRNA-155. This contributes to age-related vasoconstriction and vascular oxidative stress.^264^ Collectively, these changes accelerate the process of vascular ageing. These age-related vascular changes are further amplified through systemic neurohumoral networks. The heart‒brain axis (HBA), which functions via autonomic-RAAS-hypothalamic integrations, not only mediates cross-organ communication but also establishes feedback loops that exacerbate vascular stiffness.^268^ In summary, the RAAS, particularly the MR, drives cardiovascular ageing by regulating vascular smooth muscle cell function, thereby increasing vascular stiffness and the risk of CVD.

#### β-Adrenergic signaling

As we age, circulating catecholamine levels gradually increase, leading to sustained stimulation and eventual desensitization of cardiac β-adrenergic receptors.^269,270^ This phenomenon, observed in both elderly humans and rodents, results in impaired cardiac autonomic regulation, known as β-adrenergic desensitization,^271^ which in turn leads to reduced cardiac reserve and decreased exercise tolerance.^272,273^ While early activation of β-adrenergic signaling may transiently improve cardiac function, prolonged overactivation can trigger myocardial hypertrophy and fibrosis.^274^ Continuous stimulation leads to β-receptor desensitization, gradually weakening the regulatory capacity of the heart. Intervention with β-blockers has been shown to slow progression, improve cardiac reserve, and prolong patient survival.^275,276^ Thus, inhibiting β-adrenergic signaling is considered a protective mechanism, preventing excessive cardiac responses to long-term catecholamine stimulation. However, inhibition itself can also have negative effects, particularly by reducing cAMP production, which impairs vascular smooth muscle cell function,^277^ thereby increasing the risk of atherosclerosis and other vascular diseases.^278–281^ Additionally, β2-adrenergic receptors in EPCs promote angiogenesis through signaling with eNOS.^282^ This ability diminishes with age. Notably, strategies aimed at restoring β2-adrenergic receptor signaling have been shown to effectively improve impaired angiogenesis.^283^

#### Growth signaling

The cardiovascular system is a key target of growth hormone (GH) and insulin-like growth factor-1 (IGF-1). Evidence suggests that cardiomyocytes, vascular endothelial cells, and smooth muscle cells abundantly express IGF1-R, which is more sensitive to IGF-1 than to insulin.^284–286^ On the basis of the temporal decline in circulating IGF-1 levels and the development of cardiovascular dysfunction with ageing, a causal relationship has been proposed between reduced IGF-1 levels and the onset of cardiac and vascular ageing phenotypes.^287^ Both circulating and locally produced IGF-1 help maintain the integrity of cardiovascular function and structure by increasing NO bioavailability, reducing ROS production, and exerting anti-inflammatory, antiapoptotic, and proangiogenic effects.^288^ Recent studies have shown that GH supplementation, which significantly elevates circulating IGF-1 levels, substantially increases cortical vascular density in aged rats^289^ while also improving cognitive function.^287,290–294^ IGF-1 infusion has also been shown to increase brain microvascular density in adult mice by ~40%.^295^ Research has indicated that ageing is associated with microvascular rarefaction in the rat heart, and GH treatment has been shown to increase myocardial blood flow and capillary density in aged rats.^296^

During embryonic development, vascular endothelial growth factor (VEGF) is the primary regulator of neovascularization, stimulating the sprouting of new blood vessels from the existing vasculature. Hypoxic regions in tissues create VEGF gradients, prompting endothelial cells to differentiate into tip cells that guide capillary formation.^297^ Together, angiogenesis and vasculogenesis establish a mature, well-perfused microvascular network. However, as ageing progresses, the ability to repair blood vessels and form new ones decreases, partly due to reduced VEGF production.^298^ Impaired angiogenesis hinders the formation of new blood vessels, contributing to vascular ageing.^299^ Reduced expression of proangiogenic factors such as VEGF limits endothelial cell proliferation and blood vessel formation.^300^ In patients with chronic heart failure, insufficient angiogenesis hampers the ability of the heart to adapt to changing demands, resulting in inadequate blood supply.^301^ Transgenic overexpression of VEGF prevents the natural age-related decline in VEGF signaling; reduces endothelial cell senescence, inflammation and mitochondrial dysfunction; promotes blood perfusion of various tissues; and extends healthspan and lifespan.^302^

#### Mechanosignaling

Dysregulation of mechanotransduction coactivators, such as Yes-associated protein (YAP) and transcriptional coactivator with PDZ-binding motif (TAZ), occurs in response to mechanical stress. Inhibition of YAP/TAZ blocks JNK signaling and downregulates proinflammatory gene expression, thereby reducing monocyte adhesion and infiltration. Endothelial-specific overexpression of YAP in vivo exacerbates, whereas CRISPR/Cas9-mediated knockdown of YAP in endothelial cells delays, plaque formation in ApoE-/- mice.^303^ Moreover, maintaining YAP function can rejuvenate senescent cells and prevent the onset of ageing features by regulating cGAS-STING signaling. In contrast, genetic inactivation of YAP/TAZ in stromal cells accelerates ageing.^304^ YAP/TAZ play crucial roles in arterial stiffening during cardiovascular ageing.

As summarized in Table 1, ageing disrupts critical signaling pathways, each contributing uniquely to cardiovascular dysfunction. Targeted modulation of these pathways—such as MR antagonism, β-blockers, or low-dose GH therapy—may mitigate age-related vascular and cardiac decline (Table 2).

**Table 1:** Dysregulation of signaling pathways

Signaling Pathway	Key Mechanisms	Age-Related Changes	Cardiovascular Impact
RAAS	Disruption of sodium homeostasis, Dysregulation of vascular tone and blood volume, Abnormal cell proliferation and growth, Oxidative stress, Inflammation, Fibrotic remodeling.	**↑**Renin, Angiotensin II, ACE, Collagen IV, Fibronectin, TGF-β, MRs, *LTCC*, *Agtr1* **↓**miRNA-155	Hypertension, Atherosclerosis, Coronary artery disease, AF, Heart failure.
β-Adrenergic signaling	β-adrenergic desensitization, Impaired angiogenesis	**↑**Circulating catecholamines, **↓**β-receptor sensitivity, **↓**β_2_-adrenergic receptor signaling	Reduced cardiac reserve, Decreased exercise tolerance, Myocardial hypertrophy and fibrosis.
Growth signaling	Disruption of the integrity of microvascular function and structure	↓GH/IGF-1 levels	Microvascular rarefaction, Impaired tissue perfusion.
	Impaired angiogenesis and vasculogenesis.	↓VEGF production	
Mechanosignaling	Dysregulation of mechanotransduction coactivators.	↑YAP/TAZ activation, ↑Proinflammatory gene expression, ↓Monocyte adhesion and infiltration.	Plaque formation, Arterial stiffening.

*RAAS renin‒angiotensin‒aldosterone system; ACE angiotensin converting enzyme; TGF-β transforming growth factor-β; MRs mineralocorticoid receptors; LTCC L-type calcium channel; Agtr1 angiotensin II type 1 receptor; AF atrial fibrillation; GH/IGF-1 growth hormone/insulin-like growth factor-1; VEGF vascular endothelial growth factor; YAP/TAZ Yes-associated protein/transcriptional coactivator with PDZ-binding motif*

**Table 2:** FDA-approved drugs targeting cardiovascular ageing

Drugs	Mechanism
Metformin	Activate AMPK pathway and regulate metabolism.
Rapamycin	Inhibit mTOR.
SGLT2 inhibitors	Reduce oxidative stress, inhibit inflammation and improve vascular function.
GLP-1 receptor agonizts	Regulate insulin and glucagon secretion, delay gastric emptying and increase satiety.
ACEI	Increase NO production, improve cardiac function, metabolism and endothelia function.
ARB	Reduce peripheral vascular resistance and aldosterone secretion, lower blood pressure.
Aspirin	Decrease expression of iNOS and Cox-2
Statins	Reduce in ROS levels, increase NO synthesis and neoangiogenesis.
β-blockers	Reduce cardiac output, blood pressure and myocardial oxygen consumption
PCSK9 inhibitors	Lower LDL levels, regulate inflammation and immunity, improve endothelial function
Acarbose	Stop postprandial blood sugar spikes.
N-acetylcysteine	Antioxidant, anti-inflammatory and immunomodulatory.
sGC Agonizts	Activate cGMP pathway and promote vasodilation.
CCBs	Reduce vascular resistance associated with ageing.
DPP-4 inhibitors	Mitigate age-related glucose intolerance.

*AMPK AMP-activated protein kinase, mTOR mammalian target of rapamycin, SGLT-2 sodium-glucose cotransporter 2, GLP-1 glucagon-like peptide-1, ACEI angiotensin-converting enzyme inhibitor, NO nitric oxide, ARB angiotensin II receptor blocker, iNOS inducible nitric oxide synthase, Cox-2 cyclooxygenase-2, ROS reactive oxygen species, PCSK9 Proprotein Convertase Subtilisin/Kexin Type 9, LDL low-density lipoprotein, sGC soluble guanylate cyclase, cGMP cyclic guanosine monophosphate, CCBs calcium channel blockers, DPP-4 dipeptidyl peptidase-4*

### Crosstalk among cardiovascular ageing hallmarks

The hallmarks of cardiovascular ageing exhibit profound interconnectivity, wherein experimental amplification or suppression of a single hallmark invariably cascades to influence the majority, if not all, of the remaining features.

For example, senescent cells secrete a range of proinflammatory cytokines and chemokines, growth factors, and matrix metalloproteinases, collectively known as the SASP, which can induce “secondary” or “paracrine” senescence in neighboring nonsenescent cells.^305^ Mechanistically, inflammation and cellular senescence are closely linked through the SASP. The innate immune cGAS‒STING pathway senses cytosolic DNA and promotes downstream activation of interferon-dependent inflammatory pathways, which are essential for the acquisition of cellular senescence and the SASP.^306^ Moreover, the SASP drives inflammation, primarily through the accumulation of senescent cells and the buildup of extracellular debris and infectious pathogens that cannot be cleared due to senescence.^307^ Therefore, targeting excess senescent cells represents a promising strategy for delaying vascular ageing. In conclusion, inflammatory processes contribute to cardiac and vascular ageing, and anti-inflammatory interventions targeting key nodes of the SASP may delay or halt this process. Additionally, leveraging lineage tracing and functional studies of senescent cells in vivo to develop cell type-specific senolytic and rejuvenation strategies offers new tools and directions for advancing senescent cell clearance therapies.^235^

In addition, genomic instability can directly promote inflammation through clonal hematopoiesis of indeterminate potential (CHIP).^308^ Moreover, epigenetic alterations not only affect gene expression but also induce cellular senescence and disrupt neurohormonal signaling pathways in the ageing cardiovascular system.^190,309^ This interdependence is further reflected in experimental anti-ageing interventions, which frequently engage multiple hallmarks simultaneously through their mechanisms of action. For example, SIRT activators, such as NAD^+^ precursors, counteract multiple hallmarks of cardiovascular ageing.^310^ They mitigate genomic instability via enhanced DNA repair, reverse epigenetic alterations through histone deacetylation, restore proteostasis by promoting the clearance of protein aggregates, and ameliorate mitochondrial dysfunction by augmenting mitophagy-mediated quality control. However, metformin has pleiotropic effects, including activation of AMPK (a nutrient scarcity sensor), inhibition of mitochondrial respiration, attenuation of adipocyte senescence, suppression of proinflammatory pathways, and modulation of the gut microbiota toward a metabolically favorable composition.^311^

These interactions increase the complexity of cardiovascular ageing, indicating that abnormalities in a single mechanism may trigger widespread biological cascades. Therefore, future research on mechanisms should not focus solely on amplifying the effects of a specific hallmark but should consider the systemic interactions between these hallmarks, providing a theoretical basis for the development of multitargeted therapeutic strategies.

## The role of cardiovascular ageing in ageing-related diseases

### Cardiovascular diseases

#### Heart failure

**Fig. 5:**
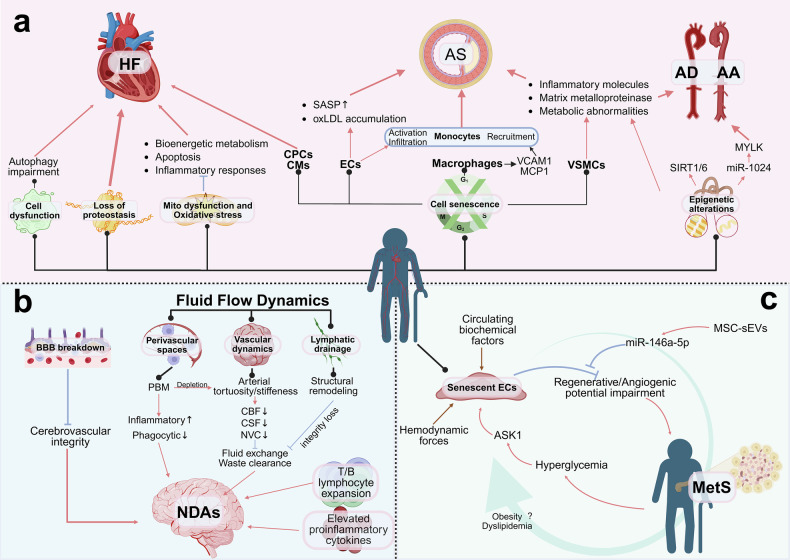
Disease-specific mechanisms linked to cardiovascular ageing. **a** Cell senescence and dysfunction, loss of proteostasis, mitochondrial dysfunction and oxidative stress all contribute to heart failure. Among these mechanisms, the loss of proteostasis plays a central role. Different senescent cells play important roles in atherosclerosis. Aortic dissection and aortic aneurysm are partly caused by cell senescence and epigenetic alterations. These factors are all hallmarks of cardiovascular ageing. **b** Blood‒brain barrier breakdown and impaired fluid flow dynamics collectively exacerbate neurodegenerative disorders associated with ageing. The latter encompasses three interdependent components: vascular dynamics, maintenance of perivascular spaces, and meningeal lymphatic drainage. Additionally, T and B lymphocyte expansion alongside elevated proinflammatory cytokines contribute to the pathogenesis of these age-related neurodegenerative conditions. **c** Circulating biochemical factors and hemodynamic forces drive cellular senescence in endothelial cells, which compromises their regenerative and angiogenic potential, thereby exacerbating the pathogenesis and progression of diabetes. This impairment can be reversed by MSC-sEVs carrying miR-146a-5p. Conversely, hyperglycemia accelerates EC senescence through ASK1 activation, thereby promoting cardiovascular ageing. Consequently, diabetes and cardiovascular ageing establish a vicious cycle. Whether obesity and hyperlipidemia—established risk factors for cardiovascular ageing—form analogous pathological circuits remains unclear. HF heart failure, CPCs cardiac progenitor cells, CMs cardiomyocytes, AS atherosclerosis, AD aortic dissection, AA aortic aneurysm, NDAs neurodegenerative disorders of ageing, MetS metabolic disorders, SASP senescence-associated secretory phenotype, ECs endothelial cells, VSMCs vascular smooth muscle cells, BBB blood‒brain barrier, PBM parenchymal border macrophage, CBF cerebral blood flow, CSF cerebrospinal fluid, NVC neurovascular coupling, MSC‒sEVs mesenchymal stem cell-derived small extracellular vesicles. Created in BioRender (2025) https://BioRender.com/fozj7xk

Heart failure (HF) is a clinical syndrome that usually occurs in elderly individuals. Each HF phenotype is determined by the patient’s risk factors, comorbidities, and disease modifiers superimposed on the cardiovascular ageing process, which act as a scaffold.^312^ Current theories of ageing mechanisms identify the progressive dysregulation of cellular protein homeostasis (proteostasis) and loss of protein quality control as central determinants of senescence, a phenomenon now termed “loss of proteostasis”. Proteostasis collapse has been mechanistically linked to HF development following myocardial infarction or valvulopathy. As evidenced in genetic forms of cardiomyopathy, disrupted proteostasis is recognized as a critical contributor to acquired cardiomyopathy pathogenesis.^313,314^ While the presence of autophagosomes does not invariably indicate increased autophagic protein degradation (i.e., increased autophagic flux), ultrastructural evidence demonstrating autophagosome-associated sarcomere degradation suggests that activated autophagic proteolysis accelerates protein turnover, thereby potentiating cardiac remodeling and disease progression in patients with cardiac hypertrophy or mitral regurgitation. Experimental studies in mice with pressure overload-induced cardiac hypertrophy demonstrated the tripartite interplay of proteotoxic stress, the induction of autophagic degradation, and the formation of protein aggregates/aggregate-like structures.^315^ These aggregates emerge when proteolytic systems become impaired, overwhelmed, or pharmacologically inhibited.^315^ Notably, cardiomyocytes from HF patients with ischemic or valvular etiologies exhibit increased autophagic proteolysis. This maladaptive response may drive excessive sarcomeric degradation, contractile dysfunction, and pathological disease progression. Crucially, the dual role of autophagy—as both a quality control mechanism and a potential contributor to proteostatic imbalance—highlights the context-dependent nature of proteostasis regulation in HFs. Apart from the loss of proteostasis, mitochondrial and oxidative stress are considered important factors in cardiac ageing and in the development of cardiac diseases such as heart failure, cardiac hypertrophy, and diabetic cardiomyopathy. As we age, cellular processes associated with mitochondrial function, such as bioenergetic metabolism, apoptosis, and inflammatory responses, change, leading to cardiac dysfunction. An in-depth understanding of the mitochondrial mechanisms associated with the ageing process will provide new strategies to ameliorate this process, particularly in heart failure.^316^ Notably, CPCs and CMs develop senescent phenotypes with increasing age. This cellular senescence suggests that senolytic approaches may provide therapeutic benefits for age-related cardiac deterioration and restore regenerative capacity in the ageing heart.^207,234^ In addition, cardiovascular ageing may ultimately trigger heart failure by causing impaired cardiac function, fibrosis, and associated changes in genetic and biological processes. In particular, in the Tgαq*44 mouse model, these changes are observed in the early stages of heart failure and persist as heart failure progresses.^317^ In senescence-accelerated mice, endothelial senescence contributes to HFpEF. The addition of a high-salt, high-fat diet accelerated endothelial senescence and promoted endothelial inflammation. This coincides with the hemodynamic and structural changes typical of HFpEF.^318^ Overall, cardiovascular ageing manifests as a decline in function and structure leading to heart failure, and targeting this process may lead to new options for the treatment and management of heart failure (Fig. 5a).

#### Atherosclerosis

Atherosclerosis is characterized by the accumulation of fibrofatty lesions in the intima and is one of the most common underlying causes of cardiovascular disease. The key factors involved in the development of atherosclerosis include endothelial dysfunction, leukocyte adhesion, foam macrophage formation, and VSMC phenotypic transition.^319^ Endothelial dysfunction is considered the initial step in atherosclerosis, and VSMCs are the major cellular origin of atherosclerotic lesions. Notably, a growing body of evidence indicates that senescent ECs are frequently found in atherosclerosis.^320^ These senescent ECs increase the secretion of SASP factors and promote the activation of monocytes and their infiltration into the subendothelial region. In addition, senescent ECs compromise endothelium integrity and permeability, facilitating the accumulation of oxidized low-density lipoprotein (LDL), which further contributes to atherosclerosis.^321,322^ Elimination of endothelial senescent cells by angptl2 knockdown could promote endothelium repair and limit the progression of atherosclerotic lesions in the aortic wall.^323^ Senescent VSMCs are considered to be a driving force of atherosclerosis, promoting plaque instability by producing inflammatory adhesion molecules, matrix metalloproteinases and metabolic abnormalities.^324^ SIRT6 has been demonstrated to delay VSMC senescence by preserving telomere integrity, thereby reducing atherosclerotic plaque burden and preserving plaque stability.^325^ An ageing-associated metabolic regulator, TRAP1, can mediate metabolic reprogramming to increase lactate-dependent H4K12la via HDAC3, promoting SASP expression and offering a new therapeutic direction for VSMC senescence and atherosclerosis.^326^ Macrophages infiltrate the arterial wall, take up lipids/cholesterol and convert into foam cells. A high accumulation of senescent foamy macrophages occurs in fatty-streaked lesions, and these cells can express VCAM1 and MCP1 to recruit circulating monocytes and further promote the development of senescent cells. This results in the production of multiple inflammatory cytokines and MMPs.^327^ In addition, the senescence of T cells, B cells, and dendritic cells significantly contributes to vascular ageing and atherosclerosis.^328^

#### Aortic dissection (AD) and aortic aneurysm (AA)

Aortic aneurysm (AA) occurs when the progressive weakening of the aortic wall causes the aorta to enlarge, whereas aortic dissection (AD) occurs when a tear forms within the aortic wall and causes blood to flow between the laminar layers of the media, thereby separating them and creating a false lumen with a severely weakened outer aortic wall.^329^ Recent studies have demonstrated that vascular ageing affects AA/AD formation. According to the proteomics results, approximately 2/3 of the proteins differentially expressed in aged vs young aortic tissue were also differentially expressed in patients with thoracic AA (TAA) compared with healthy aortic tissue.^330^ In addition, ageing induces miR-1204, which inhibits myosin light chain kinase, facilitating the acquisition of the SASP and the loss of the contractile phenotype in VSMCs. aggravates abdominal AD via the miR-1204-MYLK signaling axis.^331^ VSMC senescence has been observed in mouse and human AAA and TAA samples. Senescent VSMCs release a variety of proinflammatory cytokines and matrix-related molecules, which are correlated with AA progression. The age-related sirtuin members SIIRT1 and SIRT6 were decreased in human AAA samples. VSMC-specific Sirt1 knockout and transgenic mice have consistently shown that SIRT1 significantly attenuates AAA formation via inhibition of the p21 and nuclear factor kappa B pathways in angiotensin II and CaCl2 aortic aneurysm mouse models.^332^ The overexpression of SIRT6 significantly prevents AAA formation in an Ang II infusion model and attenuates premature senescence, the inflammatory response and neoangiogenesis in VSMCs under Ang II stimulation.^333^ SIRT6 has also been demonstrated to protect against TAA by epigenetically inhibiting vascular inflammation and senescence. The VSMC senescence/proinflammatory phenotype in AAA is partly mediated by MLK1, and MLK1 deficiency abolishes p38MAPK activity and is accompanied by reduced senescence/proinflammation in the vessel wall and cultured VSMCs.^334^ Stress-induced premature senescence (SIPS) was detected in AAAs from patients and mouse models, and SIPS promoted the transformation of VSMCs from a contractile phenotype to a synthetic phenotype, whereas inhibition of SIPS by the senolytic agent ABT263 suppressed VSMC phenotype switching.^335^ On the basis of these related studies, great progress may be made in AAA treatment by interfering with VSMC senescence.

### Neurodegenerative disorders

Neurodegenerative disorders of ageing (NDAs), such as Alzheimer’s disease, Parkinson’s disease, frontotemporal dementia, Huntington’s disease and amyotrophic lateral sclerosis, are characterized by progressive neuronal dysfunction and cognitive decline. They represent a major socioeconomic challenge in view of their high prevalence yet poor treatment.^336^ Traditionally, ageing in the healthy brain is characterized by a low-grade, chronic and sterile inflammatory process called neuroinflammation. This condition is characterized primarily by the upregulation of inflammatory responses at the brain level, contributing to the development of age-related neurodegenerative disorders.^337^ However, few studies have explored the effects of ageing on neurodegenerative disorders at the cardiovascular level.

The blood‒brain barrier (BBB) is highly specialized to protect the brain from harmful circulating factors in the blood and maintain homeostasis in the brain.^338,339^ A hallmark pathological feature of NDAs is BBB breakdown (Fig. 5b), which is observed in nearly all neurodegenerative disorders.^340^ Researchers have reported breakdown of the BBB in both rodent models and humans, which begins as early as middle age and progresses to the end of the life span.^341^ The brain endothelial glycocalyx, a key structural component of the BBB,^342^ may strongly contribute to NDAs during ageing.^343^ BBB disruption in Alzheimer’s disease (AD) has been confirmed through multiple independent postmortem human studies.^344^ Recent neuroimaging investigations revealed BBB compromise in patients with mild cognitive impairment (MCI) and early-stage AD, preceding measurable cognitive decline and other cerebral pathologies.^345^ MRI studies further demonstrated increased cerebral microbleeds—reflecting cerebrovascular integrity loss—in 25% of MCI patients and 45–78% of early AD patients during predementia stages.^346–348^ These findings suggest that BBB dysfunction is not merely a consequence but also a causative contributor to AD pathogenesis.^349,350^ Vascular cognitive impairment (VCI), defined as cognitive deficits linked to cerebrovascular disease, represents another prevalent dementia subtype.^351^ Like AD, BBB disruption is closely associated with VCI. For example, serum-derived proteins have been detected in the brain tissues of VCI patients,^352,353^ whereas elevated cerebrospinal fluid (CSF) levels of albumin and laminin have been detected in this population.^352,354^ MRI analyses additionally revealed enhanced BBB leakage in VCI patients,^355,356^ collectively implicating BBB breakdown as a critical driver of VCI pathogenesis. BBB impairment has also been documented in Parkinson’s disease (PD) animal models.^357,358^ Postmortem studies revealed increased BBB permeability in the posterior commissural putamen of PD patients.^359^ Positron emission tomography imaging has demonstrated dysfunction of the BBB transporter system in PD,^360^ and dynamic contrast-enhanced MRI has revealed increased BBB leakage,^361^ underscoring the pivotal role of BBB integrity in PD pathophysiology. In amyotrophic lateral sclerosis (ALS), blood–central nervous system barrier (BCNSB) disruption has been reported. Rodent ALS models exhibit blood‒spinal cord barrier (BSCB) rupture preceding motor neuron degeneration and neuroinflammation, with progressive deterioration during the disease course^362,363^—although one human study revealed no correlation between BSCB leakage and motor neuron pathology.^364^ Postmortem analyses revealed structural and functional impairments in BSCB-associated microvasculature within the gray and white matter of the medulla and spinal cord tissues of ALS patients.^363,365^ Emerging neuroimaging studies have further revealed early-stage BSCB dysfunction in ALS patients,^366,367^ suggesting that barrier disruption contributes to ALS pathogenesis. Within the CNS, the vasculature resides in the meningeal (dura/leptomeninges) and parenchymal compartments. Meningeal vessels exhibit location-dependent specialization: dural vessels are fenestrated and lack tight junctions, permitting macromolecule and immune cell trafficking from the blood into the dura.^368,369^ However, the precise alterations in the meningeal vasculature during ageing and neurodegeneration remain uncharacterized, representing an intriguing area for future investigations.

In addition to the structural breakdown of the BBB, changes in cerebral vascular dynamics with ageing contribute to the onset and progression of neurodegenerative disorders. Brain health is intimately connected to fluid flow dynamics that cleanse the brain of potentially harmful waste material. This system is regulated by vascular dynamics, the maintenance of perivascular spaces and lymphatic drainage in the meningeal layers. However, ageing can impinge on each of these layers of regulation, leading to impaired brain cleansing and the emergence of various age-associated neurological disorders, including Alzheimer’s and Parkinson’s diseases.^337^ Age profoundly impacts cerebrovascular dynamics. With advancing age, increased arterial tortuosity alters cerebral blood flow (CBF) patterns,^370^ whereas progressive arterial stiffening—closely associated with perivascular ECM deposition—becomes evident.^371^ During ageing, the resting luminal diameter of cerebral vessels expands, impairing vascular tone regulation. This results in reduced baseline CBF and diminished neurovascular coupling (NVC) efficiency.^372^ Concurrently, BBB permeability and CSF dynamics undergo age-dependent modifications, disrupting molecular and fluid exchange between the brain and periphery.^373,374^ Notably, modifiable age-related risk factors (e.g., hypertension) exacerbate vascular dysfunction and increase cerebrovascular disease susceptibility.^375,376^ In aged murine models, NVC impairment predominantly localizes to precapillary sphincters, potentially obstructing CSF transport and cerebral waste clearance—mechanistic perturbations implicated in cognitive decline and neurodegenerative pathogenesis.^372,377^ Parenchymal border macrophages (PBMs), comprising perivascular macrophages (PVMs) and leptomeningeal macrophages, critically regulate CSF flow dynamics. First characterized by Pío del Río-Hortega in the 1920s, PVMs were later identified as a distinct myeloid lineage.^378^ PBMs originate from embryonic yolk sac progenitors, migrate to perivascular niches, and maintain population stability throughout adulthood.^379–381^ Residing in perivascular spaces (excluding capillaries), PBM modulates CSF hydrodynamics and participates in cerebral homeostasis through ROS signaling, β-amyloid, autoimmunity and hypertension.^382,383^ ageing is correlated with PBM dysfunction: single-cell RNA sequencing reveals an age-associated shift toward proinflammatory phenotypes marked by upregulated MHC class II gene expression.^384–386^ Functionally, aged PBM exhibit diminished phagocytic capacity,^385,387^ which is correlated with impaired CSF dynamics. Furthermore, PBM interacts with perivascular fibroblasts—primary ECM producers—to regulate vascular compliance. PBM depletion drives ECM accumulation and arterial stiffening, exacerbating vasomotor dysfunction^385^ and establishing a vicious cycle. These observations align with histopathological findings in aged human brains,^386^ underscoring the role of PBM in maintaining cerebral waste clearance. ageing also induces structural remodeling of meningeal lymphatic vessels,^388,389^ characterized by reduced diameter, coverage, and branching of initial lymphatics,^374^ increased endothelial permeability,^374^ and atrophy of downstream cervical lymph nodes.^390,391^ Paradoxically, basal meningeal lymphatics display hyperplastic phenotypes.^389^ These architectural changes impair meningolymphatic function, diminishing CSF and waste efflux capacity^374,389,392^ and exacerbating age-related declines in cerebral clearance efficiency.^390,393^ Consequently, cognitive impairment and neurodegenerative risk increase.^374,394^ In combination with these effects, ageing promotes a proinflammatory meningeal milieu characterized by T/B lymphocyte expansion,^395–397^ elevated proinflammatory cytokines,^391^ and loss of lymphatic integrity. These inflammatory perturbations further reduce CSF drainage efficacy, aggravating toxic metabolite accumulation.^391^ Therapeutic strategies targeting VEGFR3 signaling to enhance meningolymphatic function have shown promise in restoring cerebral waste clearance.^374^

However, more research focusing on the impact of the ageing cardiovascular system on neurodegenerative disorders is still needed to fill the knowledge gap in this area and address age-related declines in neurologic function.

### Metabolic disorders

#### Diabetes

Age-related vascular dysfunction plays a pivotal role in the pathogenesis of multiple age-associated disorders, including impaired wound healing, heart failure, diabetes mellitus, Alzheimer’s disease, and renal diseases.^109^ The cellular senescence of endothelial cells represents a central mechanism underlying this process. ECs are persistently exposed to both circulating biochemical factors and hemodynamic forces. These factors include oxygen, nutrients (such as glucose, amino acids, and lipids), hormones (such as insulin, angiotensin II, and endothelin-1), and cytotoxic agents (including chemotherapeutic drugs).^398^ Senescent ECs exhibit significant alterations in gene expression, replicative capacity, and morphology, compromising vascular endothelial integrity by impairing regenerative/angiogenic potential and promoting pathogenic progression—ultimately driving vascular ageing pathologies such as diabetes.^399^ Critically, premature EC senescence contributes to diabetic microvascular complications, including retinopathy. Physiological angiogenesis supports wound healing through neovascularization.^400^ However, this process becomes dysregulated with ageing, manifesting as delayed healing in elderly individuals compared with younger individuals. Diabetic conditions further impair angiogenesis, exacerbating wound healing deficits.^60^ Mesenchymal stem cell-derived small extracellular vesicles (MSC-sEVs) enhance senescent EC recovery and accelerate wound closure in aged/diabetic murine models by augmenting angiogenesis, and miR-146a-5p serves as a key mediator.^401^

Notably, the diabetes-vascular ageing relationship is bidirectional (as detailed in “Etiology and Risk Factors”). Dysglycemia independently predicts increased aortic stiffness over 5 years in elderly Black adults,^402^ highlighting weight and glucose management as critical preventive measures against premature vascular ageing in African populations. Postprandial hyperglycemia—a hallmark of impaired glucose tolerance prevalent in ageing—represents a prediabetic state. Intermittent hyperglycemia accelerates endothelial senescence more potently than sustained hyperglycemia does, partially through superoxide overproduction, establishing a vicious cycle between diabetes and vascular ageing (Fig. 5c). Breaking this pathological loop at any node may mitigate progressive deterioration. Yamagishi et al. demonstrated that hyperglycemia activates ASK1, accelerating EC senescence and upregulating PAI-1, thus identifying ASK1 as a therapeutic target for diabetic vascular ageing.^36^ Recent evidence implicates NLRP3 inflammasome-mediated immunosenescence as both a precursor and a driver of diabetic vascular ageing. Senescent immune cells promote vascular dysfunction directly and indirectly via perivascular adipose tissue (PVAT) dysregulation—a unique modulator of vasomotor tone. Consequently, NLRP3 pathway inhibition may prevent early immunosenescence, alleviating diabetes-associated vascular injury, whereas senolytic T-cell-or PVAT-targeted strategies represent complementary therapeutic avenues.^403^

#### Obesity and dyslipidemia

As previously described, metabolic disorders, including obesity and dyslipidemia, constitute major contributors to cardiovascular ageing.^25,404^ Nevertheless, few investigations have addressed the reciprocal impact of vascular ageing on metabolic pathophysiology—specifically, whether endothelial dysfunction arising from vascular senescence^405^ modulates lipid metabolism. Analogous to the vicious cycle established between diabetes and cardiovascular ageing, the potential existence of similar feedback loops between vascular ageing and other metabolic diseases warrants rigorous exploration. Elucidating such bidirectional relationships may reveal novel therapeutic targets for metabolic disorders rooted in age-related vascular dysfunction.

## Turning back time with emerging rejuvenation strategies for cardiovascular ageing

In Greek mythology, the Moirai (Fates)—Clotho, Lachesis, and Atropos—personify life’s temporal boundaries: Clotho spins the thread of life, Lachesis measures its length, and Atropos severs it with relentless shears, symbolizing the irrevocable nature of mortality.

**Fig. 6:**
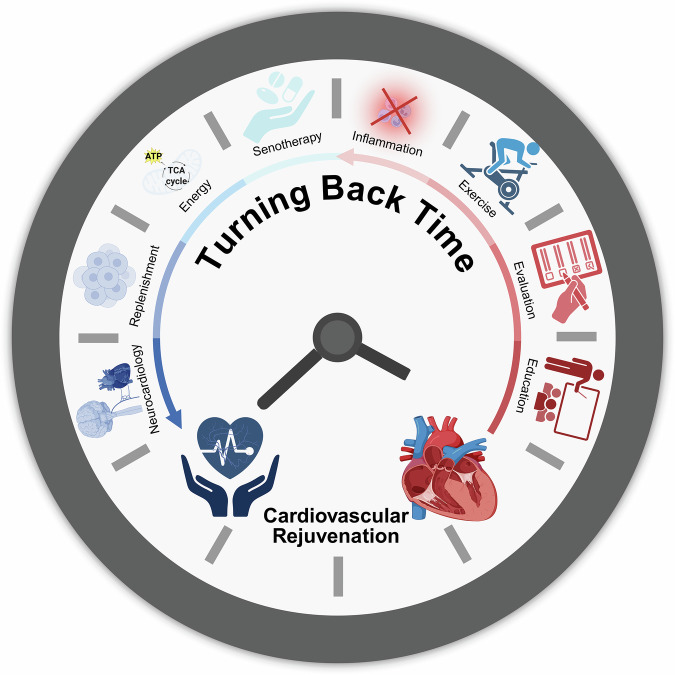
Specific strategies for cardiovascular rejuvenation. Through health education and ageing evaluation, both doctors and patients can gain a clearer understanding of ageing and the extent of its progression. Lifestyle interventions such as exercise and dietary plans are adaptive strategies to counteract cardiovascular ageing. Furthermore, targeted therapeutics such as immunotherapy, clearance of senescent cells and stem cell replenishment are being tested to alleviate cardiovascular ageing. In addition, psycho-cardiological therapy may also be an effective strategy for age-related CVDs. *Created in BioRender (2025)* https://BioRender.com/e79x749

However, humanity’s quest to prolong life has persisted unabated across civilizations. From alchemical elixirs of immortality in antiquity to contemporary geroscience targeting fundamental ageing mechanisms, mankind has relentlessly sought to transcend these biological constraints encoded in our cellular machinery. This enduring endeavor reflects not merely a defiance of mythological predestination but also a scientific odyssey to decode the molecular signatures of senescence and reshape cardiovascular ageing trajectories through targeted interventions. The etiology, risk factors and hallmarks discussed above reveal the complex biological underpinnings of cardiovascular ageing. However, understanding these mechanisms alone is insufficient to effectively address cardiovascular ageing. On the basis of these findings, it is essential to explore specific management strategies aimed at the targeted prevention and treatment of age-related CVD (Fig. 6).

### Targeting senescent cells

Since the 1960s, when the cell biologist Leonard Hayflick proposed the “replicative senescence” theory,^406^ senescent cells have been characterized by an irreversible cell cycle arrest state. Upon entering this terminally arrested phase, cells become trapped in a temporally constrained state, progressively advancing toward functional decline. Senescent cells typically exhibit upregulated expression of multiple cytokines and increased secretory capacity, a phenomenon termed the SASP.^407^ This process exacerbates chronic sterile inflammation and tissue dysfunction. Consequently, cellular senescence is recognized as a critical driver of organismal ageing and age-related pathologies. Thus, targeting senescent cells has emerged as a viable anti-ageing strategy, primarily through three approaches: immunotherapy, gene delivery and immunological interventions.

Three types of immunotherapies are used: enolytics (targeted elimination of senescent cells), senomorphics (suppression of the SASP), and the reverse (reversal of stress-induced cell cycle arrest). Among these, senolytics represent the most extensively investigated senotherapeutic approach and are currently used in clinical studies.^408,409^ Notably, proof-of-concept studies in preclinical models have established that senolytic interventions not only treat but also prevent, delay, or ameliorate age-related disorders. A seminal study of senolytics, published in 2011, demonstrated that clearance of p16^+^ senescent cells delays age-related pathologies.^17^ This was followed in 2015 by the first pharmacological validation of senolytic efficacy in aged mice.^410^ Navitoclax—a senolytic agent—ameliorates age-related cardiac dysfunction in murine models, concurrently reducing CD8^+^ effector memory T-cell populations.^411^ Senolytics have demonstrated therapeutic potential against various age-related pathologies and may synergize with conventional therapies in oncology.^412^ Senomorphics—pharmacological agents that modulate critical senescence features (e.g., SASP) without eliminating SnCs—serve as alternative therapeutic candidates.^413^ Although multiple signaling pathways contribute to the production of proinflammatory SASP factors, the most frequently studied pathways include the mTOR, p38 MAPK, and NF-κB pathways.^414^ The miR-302b-mediated reverse strategy has shown precision in reversing senescence-associated proliferative arrest, significantly extending the organismal lifespan, ameliorating ageing phenotypes, and attenuating chronic inflammation without detectable safety concerns, thereby validating the feasibility of this approach both theoretically and practically.^415^ The boundaries between senotherapeutic modalities remain fluid. For example, mTOR inhibitors may function as senolytics under specific conditions^416^ or senomorphics^417,418^ or delay senescence progression depending on context.^419^ Similarly, the flavonoid procyanidin C1 exhibits dose-dependent dual functionality as either a senomorphic or a senolytic agent.^420^ Despite their therapeutic promise, these strategies face inherent limitations. Although more than 20 clinical trials are currently ongoing and no major complications have been reported to date, senolytic therapies risk tissue damage when applied to environments with high senescent cell burdens, as evidenced by delayed wound healing—a primary adverse effect observed in INK-ATTAC mice.^17^ Emerging concerns include hepatic fibrosis secondary to vascular leakage following elimination of senescent liver sinusoidal endothelial cells.^421^ Compared with senolytics, senomorphics may require chronic administration rather than intermittent dosing, potentially increasing off-target toxicity risks. Furthermore, while effectively suppressing inflammation, senomorphics might compromise immune surveillance functions. In the opposite strategies, miR-302b successfully reverses stress-induced cell cycle arrest, yet its efficacy in postmitotic cells (e.g., neurons and cardiomyocytes) remains underexplored.

In addition to immunotherapy, gene delivery-mediated targeted clearance of senescent cells has emerged as a highly promising therapeutic strategy. Therefore, selecting the appropriate target is critical. p16 is widely used as a marker of senescent cells.^207^ p16^tdTom^ reporter mice, which use fluorescence to visualize senescent cells, allow for cell sorting and the study of senescence mechanisms, enabling targeted senescent cell clearance.^422^ p16^3MR^ reporter mice have demonstrated that clearing senescent cells improves age-related diseases such as atherosclerosis.^423^ However, a recent preprint raised concerns about the validity of p16^3MR^ mice,^424^ necessitating further research to validate this model and studies built upon it. In the p16-INK-ATTAC transgenic murine model—engineered with a “suicide” transgene under the control of the p16^Ink4a^ promoter to enable senescent cell-specific apoptosis—pharmacologically mediated clearance of senescent cells significantly improves cardiovascular function. The core design principle of this INK-ATTAC system utilizes the p16^Ink4a^ promoter to drive inducible caspase-8 expression, thereby achieving targeted elimination of p16^Ink4a+^ senescent cells. Studies have confirmed that transgene activation efficiently depletes senescent cell populations. Critically, eliminating these cells in aged subjects mitigates established age-related pathologies. In addition to p16, other markers of senescent cells can also serve as potential targets for gene delivery. Similar to the p16-INK-ATTAC model, the p21-ATTAC mouse model also enables efficient clearance of senescent cells and has demonstrated enhanced efficacy in certain disease contexts.^425^ The development of the p21-Cre mouse model has facilitated in vivo monitoring, sorting, imaging, elimination, and manipulation of p21^high^ cells, providing a powerful tool for investigating the biology of senescent cells.^426^ In p19-DTR mice, targeted removal of p19^ARF^-expressing senescent cells in lung tissue prevents elastase-induced pulmonary dysfunction.^427^ These findings substantiate that cellular senescence acts as a causal contributor to age-associated phenotypes, whereas senescent cell clearance prevents or delays tissue dysfunction and extends the healthspan.^17,428^ Overall, the advantage of targeting senescent cells is that they promote tissue regeneration and repair, offering a promising approach to slow age-related cardiovascular decline.^307^ However, in certain cases, senescent cells may have beneficial effects,^429–431^ and excessive clearance of these cells could lead to the loss of cellular and tissue functions, potentially disrupting the balance and stability of the entire physiological system.^421^ Therefore, carefully weighing the therapeutic benefits and potential side effects and identifying more precise methods to clear senescent cells are crucial. Bin Zhou et al.,^235^ on the basis of a dual-homologous recombinase system, established cell senescence lineage tracing techniques and developed four genetic strategies, laying the theoretical foundation for precision-targeted therapies. As therapies targeting stem cell exhaustion, such as replenishment, have already been summarized elsewhere, we do not discuss them further here.^432^

Immunotherapy targeting SnCs represents an emerging therapeutic strategy that primarily functions by augmenting immune-mediated clearance of SnCs. These approaches leverage the intrinsic surveillance mechanisms of the immune system to eliminate SnCs through multiple modalities. Key immune effectors, including natural killer (NK) cells, macrophages, and T-cell subsets (CD8^+^ cytotoxic and CD4^+^ helper T cells), have been implicated in SnC surveillance and clearance.^433–435^ However, SnCs may evade immune detection through the expression of inhibitory receptors and checkpoint proteins—such as programmed death-ligand 1/2 (PD-L1/PD-L2), CD80, and HLA-E—or via alternative mechanisms, including matrix metalloproteinase-mediated shedding of the NK cell costimulatory receptor NKG2D.^436–439^ Owing to the clinical success of checkpoint inhibitors in oncology,^440^ their repurposing to enhance immune-mediated SnC elimination has been actively explored. Additionally, immunotherapy targeting senescence-specific surface antigens—including dipeptidyl peptidase-4 (DPP4), urokinase-type plasminogen activator receptor (uPAR), β2-microglobulin (B2M), and glycoprotein nonmetastatic melanoma protein B (GPNMB)—is under development.^441,442^ These antigens may be exploited through chimeric antigen receptor (CAR)-T-cell engineering, monoclonal antibodies, or vaccine-based strategies. Vaccination approaches to prime adaptive immunity against SnC-derived neoantigens are being investigated. The use of specific antibodies and antibody‒drug conjugates (ADCs) has further expanded the immunotherapeutic arsenal against SnCs. Collectively, these immune-focused modalities provide promising alternatives for senescent cell clearance, with potential applications in mitigating age-related pathologies and extending the healthspan.

### Adjusting energy sensor pathways

In relation directly to the influence of diet, energy sensor pathways are important regulatory mechanisms controlling cardiac homeostasis. The mTOR pathway plays crucial roles in many physiological and pathological processes, particularly in ageing. Rapamycin, a well-known mTOR inhibitor, has been widely used in research and clinical antiageing therapies.^443^ Manipulation of proline-rich AKT1 substrate 1 (PRAS40; also known as AKT1S1), an endogenous inhibitor of mTOR complex 1 (mTORC1) signaling, also confers cardioprotection after ischemic damage and prevents diabetic cardiomyopathy in obese mice.^444^ However, the adverse effects of pharmacological mTOR inhibitors hinder their development as anti-ageing therapies. Low doses of one rapalogue have been demonstrated to safely enhance immune function in elderly patients.^445^ Future research aimed at identifying mTOR inhibitors with lower toxicity and optimizing dosing regimens could uncover strategies to promote healthy cardiac phenotypes while mitigating adverse effects. SIRT1 is a family of redox-sensitive nicotinamide adenine dinucleotide-dependent deacetylases that catalyze the posttranslational modification of hundreds of proteins that are involved in metabolism and cellular homeostasis. More importantly, SIRT1 plays a role in ageing regulation by integrating multiple signaling pathways. Postnatally, SIRT1 expression decreases in most organs but remains elevated in healthy hearts unless myocardial ageing or cardiomyopathy manifests.^446–448^ Mild to moderate SIRT1 upregulation exerts cardioprotective effects,^449^ as demonstrated in multiple experimental models.^450^ SIRT1 activation triggers antioxidant mechanisms to reduce oxidative stress and promote mitochondrial health and biogenesis while simultaneously suppressing proinflammatory pathways in cardiomyocytes, thereby increasing cellular survival.^451,452^ Its activator, resveratrol, is considered an effective anti-ageing drug because its broad biological activities help mitigate the negative effects of ageing.^126^ However, while most healthy subjects can tolerate doses of up to 2.5 g per day well, some may also experience reactions such as nausea, stomach pain, or headache as a result of ingesting high doses of resveratrol.^453^ Owing to interindividual differences, it has not yet been possible to determine an optimal dosage for all people. In addition to these classic drugs, recent discoveries have shown that the ability to activate the AMP-activated protein kinase (AMPK) pathway decreases with age, impairing cellular homeostasis and accelerating the ageing process. Metformin, an AMPK activator, has been shown to slow ageing through various mechanisms.^311^ Metformin has been extensively utilized as a first-line therapeutic for diabetes mellitus since the 1990s. Subsequent studies demonstrated its capacity to extend the healthspan in *Caenorhabditis elegans* and both the healthspan and lifespan in murine models,^454,455^ although contradictory evidence revealed no lifespan extension in mice.^456^ Metformin has been used in humans for more than six decades; it has a well-characterized safety profile and uniquely targets multiple pathways implicated in ageing and age-related pathologies.^457^ Epidemiological analyses revealed geroprotective effects, including a reduced incidence of age-related comorbidities and attenuated all-cause mortality in diabetic and nondiabetic populations.^458,459^ Nevertheless, this renowned therapeutic agent has inherent adverse effects, most notably gastrointestinal disturbances—such as abdominal pain, early satiety, appetite suppression, and diarrhea—which typically resolve within 1–2 weeks of treatment initiation.^460^ Few patients may complain about anxiety, sleeplessness, fast breathing, and other symptoms soon after taking metformin and will usually stop the drugs on their own. Metformin increases lactate levels while maintaining the normal range, but it is possible that those with severe side effects, including MALA, may be more sensitive to the inhibition of mitochondrial complex I, which is attributable to genetic mechanisms.^311^

Like many other biological processes, ageing is regulated by classical signaling pathways and transcription factors, and interventions targeting these pathways can alter the ageing process and delay disease onset. However, it remains unclear whether drugs such as rapamycin and metformin directly modulate the ageing process or act through indirect mechanisms. For example, rapamycin may exert its effects via immunosuppression, whereas metformin may function through glycemic control. Therefore, further exploration of classical ageing regulatory pathways may promote the development of specific therapeutic strategies for cardiovascular ageing.

### Addressing central inflammatory pathways

As we reviewed before, in “inflammageing”, key players such as TNF-α, IL-1β, and IL-6 are central.^172^ Targeting central inflammatory pathways, such as the use of the IL-1β antagonist canakinumab, has been shown to improve chronic inflammation in the microcirculation, thereby reducing cardiovascular ageing.^175^ T-cell-specific deficiency in mitochondrial transcription factor A (TFAM) is sufficient to drive cardiovascular ageing, accompanied by increased circulating cytokines. The TNF-α inhibitor etanercept partially reverses this phenotype.^174^ Additionally, chemokines play critical roles in inflammation and immunity,^461^ and targeting chemokines is a promising therapeutic approach.^462^ There is substantial evidence indicating that targeting central inflammatory pathways can improve cardiovascular ageing. However, a large phase III clinical trial involving aspirin in individuals over 70 years of age produced negative results,^463^ leaving questions about which patient populations may benefit from anti-inflammatory interventions. In addition, targeting inflammation via TNF-α inhibitors such as etanercept ameliorates age-related cardiovascular dysfunction, yet concomitant immunosuppression may elicit adverse off-target effects. Kopp et al. demonstrated that patients with spondyloarthropathies (SpAs) receiving anti-TNFα therapy presented an elevated risk of noninfectious inflammatory events (NIEs) compared with untreated cohorts,^464^ suggesting that systemic immunosuppression could counteract these benefits in ageing populations. As the inflammatory system is redundant, compensatory, and crucial for survival, evaluation of risks as well as benefits must drive the development of agents in this class.

### Modulating neurocardiology dynamics

The physiological connection between the heart and the brain, known as HBA, operates through a complex network involving the autonomic nervous system, the RAAS, and the hypothalamic‒pituitary axis and plays a significant role in common diseases.^268^ The influence of heart–brain interactions on cardiovascular health should not be underestimated. Sleep deprivation, a widespread phenomenon in modern society, has been shown to cause cardiac dysfunction, including increased heart rate variability, elevated blood pressure, and myocardial injury, thereby exacerbating the risk of CVD.^465^ Cardiogenic regulation of sleep after heart injury, which restricts cardiac sympathetic input, limits inflammation and damage.^466^ Depression, a common mental disorder, not only affects mood and cognitive function but is also closely associated with increased morbidity and mortality in patients following acute myocardial infarction, highlighting the potential threat that mental health issues pose to cardiovascular health.^32,467^ We have shown that stress-related neural activity increases the risk of cardiovascular events by influencing the vulnerability of coronary artery plaques and promoting coronary inflammation, further highlighting the close connection between mental and cardiovascular health.^468^ In addition, family or friend incarceration was strongly associated with indices of EVA. The mass incarceration of others may affect the physical health of African American women, which may contribute to CVD disparities.^31^ This may represent a new therapeutic direction. In brief, psycho-cardiological therapy, which simultaneously addresses mental and cardiac health, may be an effective strategy for managing CVD driven by cardiovascular ageing.

### Adopting healthy lifestyles

Smoking causes a decrease in life expectancy, premature biological ageing, and early onset of multiple diseases; thus, quitting smoking may be a good choice.^20^ Maintaining healthy body weight throughout life is crucial for healthy ageing and longevity, reflecting the importance of caloric restriction (such as the Mediterranean diet^143^) and alternative approaches, such as “lazy strategies” (e.g., GLP-1 receptor agonizts^469,470^), which may benefit heart function. However, the use of GLP-1 receptor agonizts may lead to muscle mass reduction,^471^ necessitating the development of therapies that specifically target fat tissue without influencing muscle. Given the importance of nutrition in ageing, dietary supplements offer a popular and convenient method for maintaining or restoring youthfulness in an ageing population.^472^ For example, spermidine increases lifespan and improves cardiac function in aged mice and rats, which is correlated with a reduced incidence of CVD in humans.^199^ While adhering to the core principles of a healthy diet, it is important to tailor dietary recommendations on the basis of individual preferences, culture, and the nutritional needs of the ageing population.^473^ Additionally, exercise, a “medicine” available without prescription, plays an irreplaceable role in cardiovascular health. An appropriate exercise regimen not only slows cardiovascular ageing but also improves overall quality of life.^474^ Embracing a healthy lifestyle can significantly delay or reverse the structural and functional changes caused by cardiac ageing and thus reduce the risk of age-related chronic diseases.^318,475–477^

### Assessing and preventing the degree of ageing

Research efforts to identify biomarkers quantifying biological age—particularly multiomics-based biomarkers—have increased in recent years. These biomarkers not only predict ageing-related health outcomes but also serve as surrogate endpoints for evaluating interventions promoting healthy longevity. Comprehensive reviews have synthesized evidence on the predictive validity of omics biomarkers in population-based ageing studies.^478^ Ageing clocks comprehensively monitor multiple dimensions of ageing, including hormonal signaling, lipid metabolism, chronic inflammation, and systemic manifestations. These clocks hold translational potential, enabling the identification of at-risk populations and guiding precision medicine.^479^ Notably, DNA methylation (DNAm) in ageing clocks and their multimodal derivatives can effectively predict biological age and may provide further insights into factors influencing ageing rates.^480^ However, while machine learning models that use DNAm data accurately predict biological age, they often lack causal insights. Epigenome-wide Mendelian randomization leveraging large-scale genetic data can identify CpG sites potentially causally linked to ageing phenotypes. By mapping CpG sites with putative causal relationships with lifespan and healthspan, this approach facilitates the development of ageing biomarkers, the evaluation of anti-ageing interventions and investigations into the reversibility of age-related changes.^481^ Experimental validation confirmed the utility of a 3-CpG estimator for murine blood age prediction. A novel epigenetic age (EA) model was further developed for the mouse aorta, revealing significant correlations between epigenetic age acceleration (EAA) in blood/aortic samples and age-dependent endothelial dysfunction.^482^ Additionally, large-scale plasma proteomics combined with machine learning can noninvasively assess organ health and ageing.^483^ The SenMayo gene set enables the precise identification of senescent cells across different tissues and species, allowing the characterization of senescent cells at the single-cell level and the identification of key intercellular signaling pathways.^484^ By employing a comprehensive assessment from the individual level to the cellular level and leveraging various technological approaches, these tools provide comprehensive frameworks for targeting cardiovascular ageing and related disorders, such as hypertension.^485^

## Conclusion and future perspectives

With age, the cardiovascular system gradually degenerates, resulting in a series of pathophysiological changes. It may precede or even underlie body-wide, age-related health deterioration. In addition to the increase in time itself, we have innovatively summarized five major etiological factors as well as risk factors that have the potential to promote cardiovascular ageing (Fig. 2). On this basis, we propose several specific management strategies for cardiovascular ageing (Fig. 6). Tables 2 and 3 list FDA-approved drugs and clinical trials targeting cardiovascular ageing. However, it is important to note that categorization does not mean that the etiologies are independent of each other. A poor lifestyle (e.g., diet) can lead to obesity,^486^ which, as a metabolic syndrome, can accelerate cardiovascular ageing.^25^ Together, all the etiologies and risk factors form a complex ‘spider web’ of cardiovascular ageing. Obviously, focusing on only one aspect of the problem is limited and ineffective. Therefore, future research should take a holistic approach to propose a macroscopic framework for the etiology of cardiovascular ageing and corresponding therapeutic improvement methods.

**Table 3:** Clinical trials targeting cardiovascular ageing

NCT Number	Study Title	Study Status	Interventions
NCT01395277	Role of Flavanols In Cardiovascular Function in Healthy ageing	COMPLETED	DIETARY_SUPPLEMENT: High Flavanol first then Low Flavanol|DIETARY_SUPPLEMENT: Low Flavanol first then High Flavanol
NCT05301192	Angiotensin-(1-7) Cardiovascular Effects in ageing	RECRUITING	DRUG: Angiotensin-(1-7) | DRUG: Saline
NCT05235958	VascuFit: Exercise and Vascular ageing	COMPLETED	OTHER: nonlinear periodized exercise (NLPE) | OTHER: Exercise counseling
NCT03535844	Cardio-vascular Protective Effects of Wolfberry in Middle-aged and Older Adults	COMPLETED	OTHER: Wolfberry|OTHER: Healthy diet
NCT01883271	Effect of Aerobic Interval Training on Cardiovascular Function in ageing	COMPLETED	OTHER: High intensity aerobic interval training|OTHER: Continuous moderate intensity exercise
NCT04344873	Impact of T Cells on Age-related Vascular Dysfunction: A Translational Approach	NOT_YET_RECRUITING	OTHER: Placebo|DRUG: Abatacept 10 mg/kg
NCT01575288	Oral Trehalose Therapy to Reverse Arterial ageing in Middle-Aged and Older Adults	COMPLETED	DRUG: Placebo|DRUG: High-dose trehalose
NCT01775865	Targeting Inflammation to Treat Cardiovascular ageing	COMPLETED	DRUG: Salsalate|DRUG: Placebo (for salsalate)
NCT05872139	Role of Mitochondrial-derived Oxidative Stress to Promote Vascular Endothelial Dysfunction in Nonexercisers With ageing	COMPLETED	DIETARY_SUPPLEMENT: Placebo|DIETARY_SUPPLEMENT: Mitoquinone Mesylate
NCT05598359	TA-65 and ageing Associated Microvascular Dysfunction	NOT_YET_RECRUITING	DIETARY_SUPPLEMENT: TA-65 | OTHER: Placebo
NCT04530916	Wild Blueberries and Cardiovascular Health in Middle-aged/Older Men and Postmenopausal Women	RECRUITING	DIETARY_SUPPLEMENT: Blueberry Powder|DIETARY_SUPPLEMENT: Placebo Powder
NCT01417663	Effects of Exercise Training and AGE-crosslink Breaker on Cardiovascular Structure and Function	COMPLETED	DRUG: Alt-711 | BEHAVIORAL: Physical exercise training
NCT03476785	Prevention of Cardiovascular Stiffening with ageing and Hypertensive Heart Disease	COMPLETED	BEHAVIORAL: High intensity exercise|BEHAVIORAL: Yoga
NCT01953705	n-3 PUFA for Vascular Cognitive ageing	UNKNOWN	DRUG: Omega 3 PUFA | DRUG: Placebo
NCT01891513	ACE Inhibitors Combined with Exercise for Seniors - Pilot Study	COMPLETED	BEHAVIORAL: Exercise|DRUG: ACE inhibitor + exercise|DRUG: Thiazide diuretic + exercise|DRUG: Angiotensin receptor blocker + exercise
NCT05706181	Heat Therapy, Functional Capacity, and Vascular Health in Older Adults	RECRUITING	OTHER: Home-based leg heat therapy|OTHER: Home-based sham therapy
NCT04763291	Cardiovascular and Inflammageing Study	RECRUITING	DIETARY_SUPPLEMENT: Juice Plus+ Fruit, Vegetable and Berry blends|DIETARY_SUPPLEMENT: Juice Plus+ Omega blend
NCT01842399	Resveratrol and Cardiovascular Health in the Elderly	TERMINATED	DIETARY_SUPPLEMENT: Resveratrol|DRUG: Placebo
NCT03821623	Nicotinamide Riboside for Treating Elevated Systolic Blood Pressure and Arterial Stiffness in Middle-aged and Older Adults	RECRUITING	DRUG: Nicotinamide riboside|OTHER: Placebo
NCT04588649	The ageing Brain and Cognition: Contribution of Vascular Injury, Amyloid Plaque and Tau Protein to Cognitive Dysfunction After Stroke	COMPLETED	DRUG: THK-5351 | DRUG: AV-45
NCT05433233	Effects of Lifestyle Walking on Blood Pressure in Older Adults with Hypertension	COMPLETED	BEHAVIORAL: Walking|OTHER: HAPA Behavior Change Counseling
NCT03295734	ACES - ACE Inhibitors Combined with Exercise for Seniors with Hypertension	COMPLETED	BEHAVIORAL: Aerobic exercise|DRUG: Perindopril|DRUG: Losartan|DRUG: HCTZ
NCT03370991	Blueberries for Improving Vascular Endothelial Function in Postmenopausal Women with Elevated Blood Pressure	COMPLETED	DIETARY_SUPPLEMENT: Blueberry Powder|DIETARY_SUPPLEMENT: Placebo Powder

*ACE angiotensin-converting enzyme, ARB angiotensin II receptor blocker, HAPA health action process approach, HCTZ hydrochlorothiazide, NLPE nonlinear periodized exercise, PUFA polyunsaturated fatty acid(s), AGE advanced glycation end-product(s)*

For the first time, we have grouped the twelve hallmarks of cardiovascular ageing into three broad categories, from the micro- to the macrolevel, which are the molecular, cellular and systemic levels (Fig. 4). These findings collectively reveal that cardiovascular ageing is a complex process driven by the accumulation of multiple factors. Importantly, these hallmark features do not act independently but are intricately intertwined and synergistically interact, collectively exerting profound effects on ageing-related diseases (Fig. 5). However, many issues remain to be resolved. For example, the search for more specific ageing biomarkers than p16 remains an important direction for future research. In addition, different cardiac cells undergo senescence, either in naturally senescent or drug-induced senescent hearts.^487^ It remains unknown whether they play the same role in cardiac function or in the development of CVD.^208^ Whether reciprocal feedback loops exist between vascular ageing and nondiabetic metabolic disorders represents a compelling hypothesis that warrants further mechanistic investigation.

In conclusion, the ageing process is central to the development of CVD. It is crucial to understand and intervene in this degeneration process. Future research should continue to focus on integrating mechanisms and therapeutic strategies to address the global challenges of ageing and the high incidence of CVD.

## References

[1] Mensah, G. A., Roth, G. A. & Fuster, V. The global burden of cardiovascular diseases and risk factors. *J. Am. Coll. Cardiol.* **74**, 2529–2532 (2019). 31727292 10.1016/j.jacc.2019.10.009

[2] Zhen, Y. & Lin, C. The current status and prospect of cardiovascular disease in China. *Hainan Med* **24**, 1873–1876 (2013).

[3] Lakatta, E. G. & Levy, D. Arterial and cardiac aging: Major shareholders in cardiovascular disease enterprises: part I: aging arteries: a ‘set up’ for vascular disease. *Circulation* **107**, 139–146 (2003). 12515756 10.1161/01.cir.0000048892.83521.58

[4] Christou, D. D. & Seals, D. R. Decreased maximal heart rate with aging is related to reduced β-adrenergic responsiveness but is largely explained by a reduction in intrinsic heart rate. *J. Appl. Physiol.* **105**, 24–29 (2008). 18483165 10.1152/japplphysiol.90401.2008 PMC2494835

[5] Horn, M. A. & Trafford, A. W. Aging and the cardiac collagen matrix: Novel mediators of fibrotic remodelling. *J. Mol. Cell. Cardiol.* **93**, 175–185 (2016). 26578393 10.1016/j.yjmcc.2015.11.005 PMC4945757

[6] Strait, J. B. & Lakatta, E. G. Aging-associated cardiovascular changes and their relationship to heart failure. *Heart Fail. Clin.* **8**, 143–164 (2012). 22108734 10.1016/j.hfc.2011.08.011 PMC3223374

[7] Ren, J. et al. The Aging Biomarker Consortium represents a new era for aging research in China. *Nat. Med.* **29**, 2162–2165 (2023). 37468667 10.1038/s41591-023-02444-y

[8] Ding, Y.-N., Wang, H.-Y., Chen, H.-Z. & Liu, D.-P. Targeting senescent cells for vascular aging and related diseases. *J. Mol. Cell. Cardiol.* **162**, 43–52 (2022). 34437878 10.1016/j.yjmcc.2021.08.009

[9] Regnault, V., Challande, P., Pinet, F., Li, Z. & Lacolley, P. Cell senescence: basic mechanisms and the need for computational networks in vascular ageing. *Cardiovasc. Res.* **117**, 1841–1858 (2021). 33206947 10.1093/cvr/cvaa318

[10] Triposkiadis, F., Xanthopoulos, A. & Butler, J. Cardiovascular aging and heart failure: JACC review topic of the week. *J. Am. Coll. Cardiol.* **74**, 804–813 (2019). 31395131 10.1016/j.jacc.2019.06.053

[11] Han, J.-D. J. The ticking of aging clocks. *Trends Endocrinol. Metab.* **35**, 11–22 (2024). 37880054 10.1016/j.tem.2023.09.007

[12] López-Otín, C., Blasco, M. A., Partridge, L., Serrano, M. & Kroemer, G. Hallmarks of aging: an expanding universe. *Cell* **186**, 243–278 (2023). 36599349 10.1016/j.cell.2022.11.001

[13] Liu, E. & Lampert, B. C. Heart failure in older adults: medical management and advanced therapies. *Geriatrics* **7**, 36 (2022). 35447839 10.3390/geriatrics7020036 PMC9029870

[14] Roh, J., Rhee, J., Chaudhari, V. & Rosenzweig, A. The role of exercise in cardiac aging: from physiology to molecular mechanisms. *Circ. Res.* **118**, 279–295 (2016). 26838314 10.1161/CIRCRESAHA.115.305250 PMC4914047

[15] Alfaras, I. et al. Pharmacological strategies to retard cardiovascular aging. *Circ. Res.* **118**, 1626–1642 (2016). 27174954 10.1161/CIRCRESAHA.116.307475 PMC4894351

[16] Li, H. et al. Targeting age-related pathways in heart failure. *Circ. Res.* **126**, 533–551 (2020). 32078451 10.1161/CIRCRESAHA.119.315889 PMC7041880

[17] Baker, D. J. et al. Clearance of p16Ink4a-positive senescent cells delays ageing-associated disorders. *Nature* **479**, 232–236 (2011). 22048312 10.1038/nature10600 PMC3468323

[18] Mohammed, S. F. et al. Left ventricular amyloid deposition in patients with heart failure and preserved ejection fraction. *JACC Heart Fail* **2**, 113–122 (2014). 24720917 10.1016/j.jchf.2013.11.004 PMC3984539

[19] Tanskanen, M. et al. Senile systemic amyloidosis affects 25% of the very aged and associates with genetic variation in *alpha2-macroglobulin* and *tau*: a population-based autopsy study. *Ann. Med.* **40**, 232–239 (2008). 18382889 10.1080/07853890701842988

[20] Rastogi, T. et al. Impact of smoking on cardiovascular risk and premature ageing: Findings from the STANISLAS cohort. *Atherosclerosis* **346**, 1–9 (2022). 35247627 10.1016/j.atherosclerosis.2022.02.017

[21] Kumboyono, K. et al. Differences in senescence of late endothelial progenitor cells in non-smokers and smokers. *Tob. Induc. Dis.* **19**, 1–8 (2021). 10.18332/tid/135320 PMC8171388 34131419

[22] Farhat, N. et al. Stress-induced senescence predominates in endothelial cells isolated from atherosclerotic chronic smokers. *Can. J. Physiol. Pharmacol.* **86**, 761–769 (2008). 19011671 10.1139/Y08-082 PMC3701584

[23] Kasch, F. W., Boyer, J. L., Van Camp, S. P., Verity, L. S. & Wallace, J. P. Effect of exercise on cardiovascular ageing. *Age Ageing* **22**, 5–10 (1993). 8438667 10.1093/ageing/22.1.5

[24] Jørgensen, A. B., Frikke-Schmidt, R., Nordestgaard, B. G. & Tybjærg-Hansen, A. Loss-of-function mutations in *Apoc3* and risk of ischemic vascular disease. *N. Engl. J. Med.* **371**, 32–41 (2014). 24941082 10.1056/NEJMoa1308027

[25] Ruperez, C., Madeo, F., De Cabo, R., Kroemer, G. & Abdellatif, M. Obesity accelerates cardiovascular ageing. *Eur. Heart J.* **46**, 2161–2185 (2025). 40197620 10.1093/eurheartj/ehaf216 PMC12167665

[26] Gómez-Sánchez, L. et al. The Association of Dietary Intake with Arterial Stiffness and Vascular Ageing in a population with intermediate cardiovascular risk—a MARK study. *Nutrients* **14**, 244 (2022). 35057425 10.3390/nu14020244 PMC8778402

[27] Patino-Alonso, M. C. et al. Factors associated with adherence to the Mediterranean diet in the adult population. *J. Acad. Nutr. Diet.* **114**, 583–589 (2014). 24209889 10.1016/j.jand.2013.07.038

[28] Strazhesko, I. D. et al. The renin-angiotensin-aldosterone system and vascular aging]. *Kardiologiia* **53**, 78–84 (2013). 24087966

[29] Khurana, S., Venkataraman, K., Hollingsworth, A., Piche, M. & Tai, T. Polyphenols: benefits to the cardiovascular system in health and in aging. *Nutrients* **5**, 3779–3827 (2013). 24077237 10.3390/nu5103779 PMC3820045

[30] Gomez-Sanchez, M. et al. Vascular aging and its relationship with lifestyles and other risk factors in the general Spanish population: early vascular ageing study. *J. Hypertens.* **38**, 1110–1122 (2020). 32371801 10.1097/HJH.0000000000002373

[31] Fields, N. D. et al. Does stress from incarceration of family and friends contribute to signs of early vascular ageing in African American women?. *J. Epidemiol. Community Health* **78**, 745–751 (2024). 39122410 10.1136/jech-2024-222227 PMC11560605

[32] Lespérance, F., Frasure-Smith, N., Talajic, M. & Bourassa, M. G. Five-year risk of cardiac mortality in relation to initial severity and one-year changes in depression symptoms after myocardial infarction. *Circulation* **105**, 1049–1053 (2002). 11877353 10.1161/hc0902.104707

[33] Janszky, I., Ahnve, S., Lundberg, I. & Hemmingsson, T. Early-onset depression, anxiety, and risk of subsequent coronary heart disease. *J. Am. Coll. Cardiol.* **56**, 31–37 (2010). 20620714 10.1016/j.jacc.2010.03.033

[34] Shah, M. et al. Environmental and genetic predictors of human cardiovascular ageing. *Nat. Commun.* **14**, 4941 (2023). 37604819 10.1038/s41467-023-40566-6 PMC10442405

[35] Guzik, T. J. & Touyz, R. M. Oxidative stress, inflammation, and vascular aging in hypertension. *Hypertension* **70**, 660–667 (2017). 28784646 10.1161/HYPERTENSIONAHA.117.07802

[36] Yokoi, T. et al. Apoptosis signal-regulating kinase 1 mediates cellular senescence induced by high glucose in endothelial cells. *Diabetes* **55**, 1660–1665 (2006). 16731828 10.2337/db05-1607

[37] Kang, H., Ma, X., Liu, J., Fan, Y. & Deng, X. High glucose–induced endothelial progenitor cell dysfunction. *Diabetes Vasc. Dis. Res* **14**, 381–394 (2017). 10.1177/1479164117719058 28718318

[38] Shakeri, H., Lemmens, K., Gevaert, A. B., De Meyer, G. R. Y. & Segers, V. F. M. Cellular senescence links aging and diabetes in cardiovascular disease. *Am. J. Physiol. Circ. Physiol.* **315**, H448–H462 (2018). 10.1152/ajpheart.00287.2018 29750567

[39] Liang, J. et al. Notch pathway activation mediated the senescence of endothelial progenitor cells in hypercholesterolemic mice. *J. Bioenerg. Biomembr.* **52**, 431–440 (2020). 32940860 10.1007/s10863-020-09853-5

[40] Tie, G., Messina, K. E., Yan, J., Messina, J. A. & Messina, L. M. Hypercholesterolemia induces oxidant stress that accelerates the ageing of hematopoietic stem cells. *J. Am. Heart Assoc.* **3**, e000241 (2014). 24470519 10.1161/JAHA.113.000241 PMC3959695

[41] Yan, W. et al. Decreased autophagy of vascular smooth muscle cells was involved in hyperhomocysteinemia-induced vascular ageing. *Clin. Exp. Pharma. Physio.* **48**, 524–533 (2021). 10.1111/1440-1681.13442 33325046

[42] Zhang, D. et al. Homocysteine accelerates senescence of endothelial cells via DNA hypomethylation of human telomerase reverse transcriptase. *Arterioscler., Thromb., Vasc. Biol.* **35**, 71–78 (2015). 25359865 10.1161/ATVBAHA.114.303899

[43] Zanoli, L. et al. Inflammation and ventricular-vascular coupling in hypertensive patients with metabolic syndrome. *Nutr., Metab. Cardiovasc. Dis.* **28**, 1222–1229 (2018). 30348591 10.1016/j.numecd.2018.08.003

[44] Dai, L., Qureshi, A. R., Witasp, A., Lindholm, B. & Stenvinkel, P. Early vascular ageing and cellular senescence in chronic kidney disease. *Comput. Struct. Biotechnol. J.* **17**, 721–729 (2019). 31303976 10.1016/j.csbj.2019.06.015 PMC6603301

[45] Cobo, G., Qureshi, A. R., Lindholm, B. & Stenvinkel, P. C-reactive protein: repeated measurements will improve dialysis patient care. *Semin Dial* **29**, 7–14 (2016). 26360923 10.1111/sdi.12440

[46] Cobo, G., Lindholm, B. & Stenvinkel, P. Chronic inflammation in end-stage renal disease and dialysis. *Nephrol. Dial. Transplant.* **33**, iii35–iii40 (2018). 30281126 10.1093/ndt/gfy175 PMC6168801

[47] Kyriakidis, N. C., Cobo, G., Dai, L., Lindholm, B. & Stenvinkel, P. Role of uremic toxins in early vascular ageing and calcification. *Toxins* **13**, 26 (2021). 33401534 10.3390/toxins13010026 PMC7824162

[48] Jankowski, J., Floege, J., Fliser, D., Böhm, M. & Marx, N. Cardiovascular disease in chronic kidney disease: pathophysiological insights and therapeutic options. *Circulation* **143**, 1157–1172 (2021). 33720773 10.1161/CIRCULATIONAHA.120.050686 PMC7969169

[49] Bridge, L. A. et al. Two cosmoses, one universe: a narrative review exploring the gut microbiome’s role in the effect of urban risk factors on vascular ageing. *Maturitas* **184**, 107951 (2024). 38471294 10.1016/j.maturitas.2024.107951

[50] Feng, Y. et al. Associations between heavy metal exposure and vascular age: a large cross-sectional study. *J. Transl. Med.* **23**, 4 (2025). 39754096 10.1186/s12967-024-06021-w PMC11697934

[51] Conroy, G. The human heart shows signs of ageing after just a month in space. *Nature* **634**, 17–18 (2024). 39313654 10.1038/d41586-024-03105-x

[52] Maejima, Y., Adachi, S., Ito, H., Hirao, K. & Isobe, M. Induction of premature senescence in cardiomyocytes by doxorubicin as a novel mechanism of myocardial damage. *Aging Cell* **7**, 125–136 (2008). 18031568 10.1111/j.1474-9726.2007.00358.x

[53] Booth, L. K., Redgrave, R. E., Folaranmi, O., Gill, J. H. & Richardson, G. D. Anthracycline-induced cardiotoxicity and senescence. *Front. Aging* **3**, 1058435 (2022). 36452034 10.3389/fragi.2022.1058435 PMC9701822

[54] Reece, A. S. & Hulse, G. K. Impact of lifetime opioid exposure on arterial stiffness and vascular age: cross-sectional and longitudinal studies in men and women. *BMJ Open* **4**, e004521 (2014). 24889849 10.1136/bmjopen-2013-004521 PMC4054659

[55] Reece Opiate dependence as an independent and interactive risk factor for arterial stiffness and cardiovascular ageing - a longitudinal study in females. *J. Clin. Med. Res.* **5**, 356–367 (2013). 23976908 10.4021/jocmr1496w PMC3748660

[56] Holroyd, K. B. et al. Framingham risk score based vascular outcomes in acute versus chronic HIV cohorts after 6 years of ART. *HIV Med.* **25**, 725–736 (2024). 38383057 10.1111/hiv.13621 PMC11153003

[57] Testai, L., Citi, V., Martelli, A., Brogi, S. & Calderone, V. Role of hydrogen sulfide in cardiovascular ageing. *Pharmacol. Res.* **160**, 105125 (2020). 32783975 10.1016/j.phrs.2020.105125

[58] Cannon, L. & Bodmer, R. Genetic manipulation of cardiac ageing. *J. Physiol.* **594**, 2075–2083 (2016). 26060055 10.1113/JP270563 PMC4933115

[59] Liberale, L., Kraler, S., Camici, G. G. & Lüscher, T. F. Ageing and longevity genes in cardiovascular diseases. *Basic Clin. Pharmacol. Toxicol.* **127**, 120–131 (2020). 32357271 10.1111/bcpt.13426

[60] Shakeri, H. et al. Neuregulin-1 attenuates stress-induced vascular senescence. *Cardiovasc. Res.* **114**, 1041–1051 (2018). 29528383 10.1093/cvr/cvy059

[61] Li-zhen, L., Chen, Z., Wang, S.-S., Liu, W. & Zhuang, X. Klotho deficiency causes cardiac ageing by impairing autophagic and activating apoptotic activity. *Eur. J. Pharmacol.* **911**, 174559 (2021). 34637700 10.1016/j.ejphar.2021.174559

[62] Wallace, R. G. et al. The role of epigenetics in cardiovascular health and ageing: a focus on physical activity and nutrition. *Mech. Ageing Dev.* **174**, 76–85 (2018). 29155255 10.1016/j.mad.2017.11.013

[63] Ibarrola, J. et al. Smooth muscle mineralocorticoid receptor as an epigenetic regulator of vascular ageing. *Cardiovasc. Res.* **118**, 3386–3400 (2023). 35020830 10.1093/cvr/cvac007 PMC10060709

[64] Bink, D. I., Pauli, J., Maegdefessel, L. & Boon, R. A. Endothelial microRNAs and long noncoding RNAs in cardiovascular ageing. *Atherosclerosis* **374**, 99–106 (2023). 37059656 10.1016/j.atherosclerosis.2023.03.019

[65] Jusic, A. et al. Noncoding RNAs in age-related cardiovascular diseases. *Ageing Res. Rev.* **77**, 101610 (2022). 35338919 10.1016/j.arr.2022.101610

[66] Boon, R. A. et al. MicroRNA-34a regulates cardiac ageing and function. *Nature* **495**, 107–110 (2013). 23426265 10.1038/nature11919

[67] Trembinski, D. J. et al. Aging-regulated anti-apoptotic long non-coding RNA Sarrah augments recovery from acute myocardial infarction. *Nat. Commun.* **11**, 2039 (2020). 32341350 10.1038/s41467-020-15995-2 PMC7184724

[68] Zhou, L.-Y. et al. The circular RNA ACR attenuates myocardial ischemia/reperfusion injury by suppressing autophagy via modulation of the Pink1/ FAM65B pathway. *Cell Death Differ* **26**, 1299–1315 (2019). 30349076 10.1038/s41418-018-0206-4 PMC6748144

[69] Salvado, R. et al. Gut microbiota and its relationship with early vascular ageing in a Spanish population (MIVAS study). *Eur. J. Clin. Investig.* **54**, e14228 (2024). 38655910 10.1111/eci.14228

[70] Bosco, N. & Noti, M. The aging gut microbiome and its impact on host immunity. *Genes. Immun.* **22**, 289–303 (2021). 33875817 10.1038/s41435-021-00126-8 PMC8054695

[71] Pang, S. et al. Longevity of centenarians is reflected by the gut microbiome with youth-associated signatures. *Nat. Aging* **3**, 436–449 (2023). 37117794 10.1038/s43587-023-00389-y

[72] Papadopoulos, P. D. et al. The emerging role of the gut microbiome in cardiovascular disease: current knowledge and perspectives. *Biomedicines* **10**, 948 (2022). 35625685 10.3390/biomedicines10050948 PMC9139035

[73] Witkowski, M., Weeks, T. L. & Hazen, S. L. Gut microbiota and cardiovascular disease. *Circ. Res.* **127**, 553–570 (2020). 32762536 10.1161/CIRCRESAHA.120.316242 PMC7416843

[74] Honarpisheh, P., Bryan, R. M. & McCullough, L. D. Aging microbiota-gut-brain axis in stroke risk and outcome. *Circ. Res.* **130**, 1112–1144 (2022). 35420913 10.1161/CIRCRESAHA.122.319983 PMC9674376

[75] Madison, A. & Kiecolt-Glaser, J. K. Stress, depression, diet, and the gut microbiota: human–bacteria interactions at the core of psychoneuroimmunology and nutrition. *Curr. Opin. Behav. Sci.* **28**, 105–110 (2019). 32395568 10.1016/j.cobeha.2019.01.011 PMC7213601

[76] Derrien, M. & Veiga, P. Rethinking diet to aid human–microbe symbiosis. *Trends Microbiol.* **25**, 100–112 (2017). 27916707 10.1016/j.tim.2016.09.011

[77] Sfera, A. et al. Endothelial senescence and chronic fatigue syndrome, a COVID-19 based hypothesis. *Front. Cell. Neurosci.* **15**, 673217 (2021). 34248502 10.3389/fncel.2021.673217 PMC8267916

[78] Meyer, K., Patra, T., Vijayamahantesh & Ray, R. SARS-CoV-2 spike protein induces paracrine senescence and leukocyte adhesion in endothelial cells. *J. Virol.* **95**, e00794–21 (2021). 34160250 10.1128/JVI.00794-21 PMC8354225

[79] Bellon, M. & Nicot, C. Telomere dynamics in immune senescence and exhaustion triggered by chronic viral infection. *Viruses* **9**, 289 (2017). 28981470 10.3390/v9100289 PMC5691640

[80] Ahuja, S. K. et al. Immune resilience despite inflammatory stress promotes longevity and favorable health outcomes including resistance to infection. *Nat. Commun.* **14**, 3286 (2023). 37311745 10.1038/s41467-023-38238-6 PMC10264401

[81] Quiros-Roldan, E., Sottini, A., Natali, P. G. & Imberti, L. The impact of immune system aging on infectious diseases. *Microorganisms* **12**, 775 (2024). 38674719 10.3390/microorganisms12040775 PMC11051847

[82] Schubert, U., Müller, M., Abdul-Khaliq, H. & Norman, M. Preterm birth is associated with altered myocardial function in infancy. *J. Am. Soc. Echocardiogr.* **29**, 670–678 (2016). 27156903 10.1016/j.echo.2016.03.011

[83] Merz, A. A. & Cheng, S. Sex differences in cardiovascular ageing. *Heart* **102**, 825–831 (2016). 26917537 10.1136/heartjnl-2015-308769 PMC5993677

[84] Garcia, M., Mulvagh, S. L., Bairey Merz, C. N., Buring, J. E. & Manson, J. E. Cardiovascular disease in women: clinical perspectives. *Circ. Res.* **118**, 1273–1293 (2016). 27081110 10.1161/CIRCRESAHA.116.307547 PMC4834856

[85] Li, X. et al. Longitudinal trajectories, correlations and mortality associations of nine biological ages across 20-years follow-up. *Elife* **9**, e51507 (2020). 32041686 10.7554/eLife.51507 PMC7012595

[86] Appiah, D., Nwabuo, C. C., Ebong, I. A., Wellons, M. F. & Winters, S. J. Trends in age at natural menopause and reproductive life span among US women, 1959-2018. *J. Am. Heart Assoc.* **325**, 1328 (2021). 10.1001/jama.2021.0278 PMC8025101 33821908

[87] Georgiopoulos, G. et al. Prolactin as a predictor of endothelial dysfunction and arterial stiffness progression in menopause. *J. Hum. Hypertens.* **31**, 520–524 (2017). 28332508 10.1038/jhh.2017.15

[88] Sharma, K. H. et al. Are Gujarati Asian Indians ‘older’ for their ‘vascular age’ as compared to their ‘Chronological age’? *QJM* **108**, 105–112 (2015). 25086109 10.1093/qjmed/hcu158

[89] Yu, Y.-L. et al. Racial and regional disparities in the risk of noncommunicable disease between sub-Saharan black and European white patients. *J. Hypertens.* **43**, 481–491 (2025). 39655600 10.1097/HJH.0000000000003930 PMC11789602

[90] Haddad, F., Hunt, S. A., Rosenthal, D. N. & Murphy, D. J. Right ventricular function in cardiovascular disease, part I: anatomy, physiology, aging, and functional assessment of the right ventricle. *Circulation* **117**, 1436–1448 (2008). 18347220 10.1161/CIRCULATIONAHA.107.653576

[91] Evin, M. et al. Left atrial aging: a cardiac magnetic resonance feature-tracking study. *Am. J. Physiol. Circ. Physiol.* **310**, H542–H549 (2016). 10.1152/ajpheart.00504.2015 26747498

[92] van de Hoef, T. P. et al. Contribution of age-related microvascular dysfunction to abnormal coronary: hemodynamics in patients with ischemic heart disease. *JACC Cardiovasc. Interv.* **13**, 20–29 (2020). 31918939 10.1016/j.jcin.2019.08.052

[93] de Moraes, R. & Tibirica, E. Early functional and structural microvascular changes in hypertension related to aging. *Curr. Hypertens. Rev.* **13**, 24–32 (2017). 28412915 10.2174/1573402113666170413095508

[94] Faria, D. et al. Age-related structural remodelling of the coronary circulation. *Cathet. Cardiovasc. Intervent.* **104**, 968–979 (2024). 10.1002/ccd.31223 39279189

[95] Finkel, T. & Holbrook, N. J. Oxidants, oxidative stress and the biology of ageing. *Nature* **408**, 239–247 (2000). 11089981 10.1038/35041687

[96] Mengozzi, A. et al. Microvascular ageing links metabolic disease to age-related disorders: the role of oxidative stress and inflammation in promoting microvascular dysfunction. *J. Cardiovasc. Pharm.* **78**, S78–S87 (2021). 10.1097/FJC.0000000000001109 34840260

[97] Vitullo, J. C., Penn, M. S., Rakusan, K. & Wicker, P. Effects of hypertension and aging on coronary arteriolar density. *Hypertension* **21**, 406–414 (1993). 8458642 10.1161/01.hyp.21.4.406

[98] Fang, Z. et al. Systemic aging fuels heart failure: molecular mechanisms and therapeutic avenues. *ESC Heart Fail* **12**, 1059–1080 (2025). 39034866 10.1002/ehf2.14947 PMC11911610

[99] Hwang, H. J., Kim, N., Herman, A. B., Gorospe, M. & Lee, J.-S. Factors and pathways modulating endothelial cell senescence in vascular aging. *Int. J. Mol. Sci.* **23**, 10135 (2022). 36077539 10.3390/ijms231710135 PMC9456027

[100] Ding, Y.-N., Tang, X., Chen, H.-Z. & Liu, D.-P. Epigenetic regulation of vascular aging and age-related vascular diseases. *Adv. Exp. Med. Biol.* **1086**, 55–75 (2018). 30232752 10.1007/978-981-13-1117-8_4

[101] Faleeva, M. et al. Sox9 accelerates vascular aging by regulating extracellular matrix composition and stiffness. *Circ. Res.* **134**, 307–324 (2024). 38179698 10.1161/CIRCRESAHA.123.323365 PMC10826924

[102] Bautista-Niño, P. K. et al. Local endothelial DNA repair deficiency causes aging-resembling endothelial-specific dysfunction. *Clin. Sci.* **134**, 727–746 (2020). 10.1042/CS20190124 32202295

[103] Andreassi, M. G. et al. Subclinical carotid atherosclerosis and early vascular aging from long-term low-dose ionizing radiation exposure. *JACC Cardiovasc. Interv.* **8**, 616–627 (2015). 25907089 10.1016/j.jcin.2014.12.233

[104] Remes, T. M. et al. Radiation-induced accelerated aging of the brain vasculature in young adult survivors of childhood brain tumors. *Neurooncol. Pract.* **7**, 415–427 (2020). 32760593 10.1093/nop/npaa002 PMC7393284

[105] López-Otín, C., Blasco, M. A., Partridge, L., Serrano, M. & Kroemer, G. The hallmarks of aging. *Cell* **153**, 1194–1217 (2013). 23746838 10.1016/j.cell.2013.05.039 PMC3836174

[106] Hoeijmakers, J. H. J. DNA damage, aging, and cancer. *N. Engl. J. Med.* **361**, 1475–1485 (2009). 19812404 10.1056/NEJMra0804615

[107] Bautista-Niño, P. K., Portilla-Fernandez, E., Vaughan, D. E., Danser, A. H. J. & Roks, A. J. M. DNA damage: a main determinant of vascular aging. *Int. J. Mol. Sci.* **17**, 748 (2016). 27213333 10.3390/ijms17050748 PMC4881569

[108] Vermeij, W. P., Hoeijmakers, J. H. J. & Pothof, J. Genome integrity in aging: Human syndromes, mouse models, and therapeutic options. *Annu. Rev. Pharmacol. Toxicol.* **56**, 427–445 (2016). 26514200 10.1146/annurev-pharmtox-010814-124316

[109] Ungvari, Z., Tarantini, S., Donato, A. J., Galvan, V. & Csiszar, A. Mechanisms of vascular aging. *Circ. Res.* **123**, 849–867 (2018). 30355080 10.1161/CIRCRESAHA.118.311378 PMC6248882

[110] de Waard, M. C. et al. Age-related motor neuron degeneration in DNA repair-deficient Ercc1 mice. *Acta Neuropathol* **120**, 461–475 (2010). 20602234 10.1007/s00401-010-0715-9 PMC2923326

[111] Borgesius, N. Z. et al. Accelerated age-related cognitive decline and neurodegeneration, caused by deficient DNA repair. *J. Neurosci.* **31**, 12543–12553 (2011). 21880916 10.1523/JNEUROSCI.1589-11.2011 PMC6703271

[112] Barnhoorn, S. et al. Cell-autonomous progeroid changes in conditional mouse models for repair endonuclease XPG deficiency. *PLoS Genet.* **10**, e1004686 (2014). 25299392 10.1371/journal.pgen.1004686 PMC4191938

[113] Raj, D. D. A. et al. Priming of microglia in a DNA-repair deficient model of accelerated aging. *Neurobiol. Aging* **35**, 2147–2160 (2014). 24799273 10.1016/j.neurobiolaging.2014.03.025

[114] Blackburn, E. H., Epel, E. S. & Lin, J. Human telomere biology: a contributory and interactive factor in aging, disease risks, and protection. *Science* **350**, 1193–1198 (2015). 26785477 10.1126/science.aab3389

[115] Yabluchanskiy, A. et al. Pharmacological or genetic depletion of senescent astrocytes prevents whole brain irradiation-induced impairment of neurovascular coupling responses protecting cognitive function in mice. *GeroScience* **42**, 409–428 (2020). 31960269 10.1007/s11357-020-00154-8 PMC7205933

[116] Greco, C. M. & Condorelli, G. Epigenetic modifications and noncoding RNAs in cardiac hypertrophy and failure. *Nat. Rev. Cardiol.* **12**, 488–497 (2015). 25962978 10.1038/nrcardio.2015.71

[117] Pepin, M. E. et al. DNA methylation reprograms cardiac metabolic gene expression in end-stage human heart failure. *Am. J. Physiol. Circ. Physiol.* **317**, H674–H684 (2019). 10.1152/ajpheart.00016.2019 PMC6843013 31298559

[118] Zheng, Y. et al. Association of cardiovascular health through young adulthood with genome-wide DNA methylation patterns in midlife: the CARDIA study. *Circulation* **146**, 94–109 (2022). 35652342 10.1161/CIRCULATIONAHA.121.055484 PMC9348746

[119] Lo, Y.-H. & Lin, W.-Y. Cardiovascular health and four epigenetic clocks. *Clin. Epigenet.* **14**, 73 (2022). 10.1186/s13148-022-01295-7 PMC9185918 35681159

[120] Yoshino, J., Baur, J. A. & Imai, S.-I. NAD+ intermediates: the biology and therapeutic potential of NMN and NR. *Cell Metab* **27**, 513–528 (2018). 29249689 10.1016/j.cmet.2017.11.002 PMC5842119

[121] Zhang, X., Zhang, Y., Sun, A. & Ge, J. The effects of nicotinamide adenine dinucleotide in cardiovascular diseases: Molecular mechanisms, roles and therapeutic potential. *Genes. Dis.* **9**, 959–972 (2022). 35685463 10.1016/j.gendis.2021.04.001 PMC9170600

[122] Li, P., Ge, J. & Li, H. Lysine acetyltransferases and lysine deacetylases as targets for cardiovascular disease. *Nat. Rev. Cardiol.* **17**, 96–115 (2020). 31350538 10.1038/s41569-019-0235-9

[123] Zeng, H. & Chen, J.-X. Microvascular rarefaction and heart failure with preserved ejection fraction. *Front. Cardiovasc. Med.* **6**, 15 (2019). 30873415 10.3389/fcvm.2019.00015 PMC6403466

[124] Maizel, J. et al. Sirtuin 1 ablation in endothelial cells is associated with impaired angiogenesis and diastolic dysfunction. *Am. J. Physiol. Circ. Physiol.* **307**, H1691–H1704 (2014). 10.1152/ajpheart.00281.2014 PMC4269697 25239805

[125] He, X. et al. Endothelial-specific SIRT3 deletion impairs glycolysis and angiogenesis and causes diastolic dysfunction. *J. Mol. Cell. Cardiol.* **112**, 104–113 (2017). 28935506 10.1016/j.yjmcc.2017.09.007 PMC5647246

[126] Baur, J. A. & Sinclair, D. A. Therapeutic potential of resveratrol: the in vivo evidence. *Nat. Rev. Drug Discov.* **5**, 493–506 (2006). 16732220 10.1038/nrd2060

[127] Zhang, Y. et al. Sirtuin 2 deficiency aggravates ageing-induced vascular remodelling in humans and mice. *Eur. Heart J.* **44**, 2746–2759 (2023). 37377116 10.1093/eurheartj/ehad381 PMC10393077

[128] Ponting, C. P. & Haerty, W. Genome-wide analysis of human long noncoding RNAs: a provocative review. *Annu. Rev. Genomics Hum. Genet.* **23**, 153–172 (2022). 35395170 10.1146/annurev-genom-112921-123710

[129] Hipp, M. S., Kasturi, P. & Hartl, F. U. The proteostasis network and its decline in ageing. *Nat. Rev. Mol. Cell Biol.* **20**, 421–435 (2019). 30733602 10.1038/s41580-019-0101-y

[130] López-Otín, C. & Kroemer, G. Hallmarks of health. *Cell* **184**, 33–63 (2021). 33340459 10.1016/j.cell.2020.11.034

[131] Sabath, N. et al. Cellular proteostasis decline in human senescence. *Proc. Natl. Acad. Sci. USA* **117**, 31902–31913 (2020). 33257563 10.1073/pnas.2018138117 PMC7749315

[132] Li, Y. et al. A mitochondrial FUNDC1/HSC70 interaction organizes the proteostatic stress response at the risk of cell morbidity. *EMBO J.* **38**, e98786 (2019). 30591555 10.15252/embj.201798786 PMC6356068

[133] Koyuncu, S. et al. Rewiring of the ubiquitinated proteome determines ageing in *C. elegans*. *Nature* **596**, 285–290 (2021). 34321666 10.1038/s41586-021-03781-z PMC8357631

[134] Kaushik, S. & Cuervo, A. M. The coming of age of chaperone-mediated autophagy. *Nat. Rev. Mol. Cell Biol.* **19**, 365–381 (2018). 29626215 10.1038/s41580-018-0001-6 PMC6399518

[135] Alberti, S. & Hyman, A. A. Biomolecular condensates at the nexus of cellular stress, protein aggregation disease and ageing. *Nat. Rev. Mol. Cell Biol.* **22**, 196–213 (2021). 33510441 10.1038/s41580-020-00326-6

[136] Gouveia, M., Xia, K., Colón, W., Vieira, S. I. & Ribeiro, F. Protein aggregation, cardiovascular diseases, and exercise training: where do we stand?. *Ageing Res. Rev.* **40**, 1–10 (2017). 28757291 10.1016/j.arr.2017.07.005

[137] Henning, R. H. & Brundel, B. J. J. M. Proteostasis in cardiac health and disease. *Nat. Rev. Cardiol.* **14**, 637–653 (2017). 28660894 10.1038/nrcardio.2017.89

[138] González-López, E. et al. Wild-type transthyretin amyloidosis as a cause of heart failure with preserved ejection fraction. *Eur. Heart J.* **36**, 2585–2594 (2015). 26224076 10.1093/eurheartj/ehv338

[139] Rainer, P. P. et al. Desmin phosphorylation triggers preamyloid oligomers formation and myocyte dysfunction in acquired heart failure. *Circ. Res.* **122**, e75–e83 (2018). 29483093 10.1161/CIRCRESAHA.117.312082 PMC5948147

[140] Zhang, C. & Cuervo, A. M. Restoration of chaperone-mediated autophagy in aging liver improves cellular maintenance and hepatic function. *Nat. Med.* **14**, 959–965 (2008). 18690243 10.1038/nm.1851 PMC2722716

[141] Madrigal-Matute, J. et al. Protective role of chaperone-mediated autophagy against atherosclerosis. *Proc. Natl. Acad. Sci. USA* **119**, e2121133119 (2022). 35363568 10.1073/pnas.2121133119 PMC9168839

[142] Tabula Sapiens Consortium* et al. The tabula sapiens: a multiple-organ, single-cell transcriptomic atlas of humans. *Science* **376**, eabl4896 (2022). 10.1126/science.abl4896 PMC9812260 35549404

[143] Fontana, L. Interventions to promote cardiometabolic health and slow cardiovascular ageing. *Nat. Rev. Cardiol.* **15**, 566–577 (2018). 29795242 10.1038/s41569-018-0026-8

[144] Colotti, C. et al. Effects of aging and anti-aging caloric restrictions on carbonyl and heat shock protein levels and expression. *Biogerontology* **6**, 397–406 (2005). 16518701 10.1007/s10522-005-4906-z

[145] Yang, L. et al. Long-term calorie restriction enhances cellular quality-control processes in human skeletal muscle. *Cell Rep* **14**, 422–428 (2016). 26774472 10.1016/j.celrep.2015.12.042

[146] Rajasekaran, N. S. et al. Human alpha B-crystallin mutation causes oxido-reductive stress and protein aggregation cardiomyopathy in mice. *Cell* **130**, 427–439 (2007). 17693254 10.1016/j.cell.2007.06.044 PMC2962423

[147] Tannous, P. et al. Autophagy is an adaptive response in desmin-related cardiomyopathy. *Proc. Natl. Acad. Sci. USA* **105**, 9745–9750 (2008). 18621691 10.1073/pnas.0706802105 PMC2474535

[148] Nikolova, V. et al. Defects in nuclear structure and function promote dilated cardiomyopathy in lamin a/C-deficient mice. *J. Clin. Investig.* **113**, 357–369 (2004). 14755333 10.1172/JCI19448 PMC324538

[149] Pattison, J. S. et al. Cardiomyocyte expression of a polyglutamine preamyloid oligomer causes heart failure. *Circulation* **117**, 2743–2751 (2008). 18490523 10.1161/CIRCULATIONAHA.107.750232 PMC2413062

[150] Nemtsova, V., Bilovol, O., Ilchenko, I. & Shalimova, A. Age-associated features of oxidative stress as marker of vascular aging in comorbid course of hypertension and type 2 diabetes mellitus. *Vessel Plus* **2**, 27 (2018).

[151] Milan, M. et al. Time-restricted feeding improves aortic endothelial relaxation by enhancing mitochondrial function and attenuating oxidative stress in aged mice. *Redox Biol.* **73**, 103189 (2024). 38788541 10.1016/j.redox.2024.103189 PMC11140804

[152] Masi, S. et al. Epigenetic remodeling in obesity-related vascular disease. *Antioxid. Redox Signal.* **34**, 1165–1199 (2021). 32808539 10.1089/ars.2020.8040

[153] Cosentino, F. et al. Final common molecular pathways of aging and cardiovascular disease: Role of the p66Shc protein. *Arterioscler., Thromb., Vasc. Biol*. **28**, (2008). 10.1161/ATVBAHA.107.156059 18162611

[154] Paneni, F., Diaz Cañestro C., Libby P., Lüscher T.F. & Camici G.G. The aging cardiovascular system: Understanding it at the cellular and clinical levels. *J. Am. Coll. Cardiol*. **69**, (2017). 10.1016/j.jacc.2017.01.064 28408026

[155] Zhou, S. et al. Repression of P66Shc expression by SIRT1 contributes to the prevention of hyperglycemia-induced endothelial dysfunction. *Circ. Res.* **109**, 639–648 (2011). 21778425 10.1161/CIRCRESAHA.111.243592

[156] Bratic, A. & Larsson, N.-G. The role of mitochondria in aging. *J. Clin. Investig.* **123**, 951–957 (2013). 23454757 10.1172/JCI64125 PMC3582127

[157] Aimo, A. et al. Oxidative stress and inflammation in the evolution of heart failure: From pathophysiology to therapeutic strategies. *Eur. J. Prev. Cardiol*. **27**, (2020). 10.1177/2047487319870344 31412712

[158] De Bock, K. et al. Role of PFKFB3-driven glycolysis in vessel sprouting. *Cell* **154**, 651–663 (2013). 23911327 10.1016/j.cell.2013.06.037

[159] Wilson, C., Lee, M. D., Buckley, C., Zhang, X. & McCarron, J. G. Mitochondrial ATP production is required for endothelial cell control of vascular tone. *Function* **4**, zqac063 (2023). 36778749 10.1093/function/zqac063 PMC9909368

[160] Tracy, E. P. et al. Aging-induced impairment of vascular function: mitochondrial redox contributions and physiological/clinical implications. *Antioxid. Redox Signal.* **35**, 974–1015 (2021). 34314229 10.1089/ars.2021.0031 PMC8905248

[161] Steenman, M. & Lande, G. Cardiac aging and heart disease in humans. *Biophys. Rev.* **9**, 131–137 (2017). 28510085 10.1007/s12551-017-0255-9 PMC5418492

[162] Sena, C. M., Leandro, A., Azul, L., Seiça, R. & Perry, G. Vascular oxidative stress: impact and therapeutic approaches. *Front. Physiol.* **9**, 1668 (2018). 30564132 10.3389/fphys.2018.01668 PMC6288353

[163] Janaszak-Jasiecka, A., Płoska, A., Wierońska, J. M., Dobrucki, L. W. & Kalinowski, L. Endothelial dysfunction due to eNOS uncoupling: molecular mechanisms as potential therapeutic targets. *Cell. Mol. Biol. Lett.* **28**, 21 (2023). 36890458 10.1186/s11658-023-00423-2 PMC9996905

[164] Layland, J., Li, J.-M. & Shah, A. M. Role of cyclic GMP-dependent protein kinase in the contractile response to exogenous nitric oxide in rat cardiac myocytes. *J. Physiol.* **540**, 457–467 (2002). 11956336 10.1113/jphysiol.2001.014126 PMC2290258

[165] Lesnefsky, E. J., Chen, Q. & Hoppel, C. L. Mitochondrial metabolism in aging heart. *Circ. Res.* **118**, 1593–1611 (2016). 27174952 10.1161/CIRCRESAHA.116.307505 PMC5009371

[166] Bonora, M. et al. Targeting mitochondria for cardiovascular disorders: therapeutic potential and obstacles. *Nat. Rev. Cardiol.* **16**, 33–55 (2019). 30177752 10.1038/s41569-018-0074-0 PMC6349394

[167] Tian, R. et al. Unlocking the secrets of mitochondria in the cardiovascular system: path to a cure in heart failure—a report from the 2018 national heart, lung, and blood institute workshop. *Circulation* **140**, 1205–1216 (2019). 31769940 10.1161/CIRCULATIONAHA.119.040551 PMC6880654

[168] Franceschi, C. et al. Inflamm-aging: an evolutionary perspective on immunosenescence. *Ann. N.Y. Acad. Sci.* **908**, 244–254 (2000). 10911963 10.1111/j.1749-6632.2000.tb06651.x

[169] Sanada, F. et al. Source of chronic inflammation in aging. *Front. Cardiovasc. Med.* **5**, 12 (2018). 29564335 10.3389/fcvm.2018.00012 PMC5850851

[170] Abdellatif, M., Sedej, S., Carmona-Gutierrez, D., Madeo, F. & Kroemer, G. Autophagy in cardiovascular aging. *Circ. Res.* **123**, 803–824 (2018). 30355077 10.1161/CIRCRESAHA.118.312208

[171] Cooke, J. P. Endotheliopathy of obesity. *Circulation* **142**, 380–383 (2020). 32718250 10.1161/CIRCULATIONAHA.120.047574 PMC7391057

[172] Wang, L., Zhang, Y. & Zhang, S. Cardiovascular impairment in COVID-19: Learning from current options for cardiovascular anti-inflammatory therapy. *Front. Cardiovasc. Med.* **7**, 78 (2020). 32426374 10.3389/fcvm.2020.00078 PMC7203508

[173] Ferrucci, L. & Fabbri, E. Inflammageing: Chronic inflammation in ageing, cardiovascular disease, and frailty. *Nat. Rev. Cardiol.* **15**, 505–522 (2018). 30065258 10.1038/s41569-018-0064-2 PMC6146930

[174] Desdín-Micó, G. et al. T cells with dysfunctional mitochondria induce multimorbidity and premature senescence. *Science* **368**, 1371–1376 (2020). 32439659 10.1126/science.aax0860 PMC7616968

[175] Everett, B. M. et al. Inhibition of interleukin-1β and reduction in atherothrombotic cardiovascular events in the CANTOS trial. *J. Am. Coll. Cardiol.* **76**, 1660–1670 (2020). 33004131 10.1016/j.jacc.2020.08.011

[176] Schafer, S. et al. IL-11 is a crucial determinant of cardiovascular fibrosis. *Nature* **552**, 110–115 (2017). 29160304 10.1038/nature24676 PMC5807082

[177] Saltiel, A. R. & Olefsky, J. M. Inflammatory mechanisms linking obesity and metabolic disease. *J. Clin. Invest.* **127**, 1–4 (2017). 28045402 10.1172/JCI92035 PMC5199709

[178] Levine, B. & Kroemer, G. Biological functions of autophagy genes: a disease perspective. *Cell* **176**, 11–42 (2019). 30633901 10.1016/j.cell.2018.09.048 PMC6347410

[179] Nicolás-Ávila, J. A. et al. A network of macrophages supports mitochondrial homeostasis in the heart. *Cell* **183**, 94–109.e23 (2020). 32937105 10.1016/j.cell.2020.08.031

[180] Abdellatif, M. & Kroemer, G. Co-ordinated mitochondrial degradation by autophagy and heterophagy in cardiac homeostasis. *Cardiovasc. Res.* **117**, e1–e3 (2021). 33393622 10.1093/cvr/cvaa345

[181] Picca, A. et al. Mitochondrial quality control mechanisms as molecular targets in cardiac ageing. *Nat. Rev. Cardiol.* **15**, 543–554 (2018). 30042431 10.1038/s41569-018-0059-z PMC6283278

[182] Abdellatif, M., Ljubojevic-Holzer, S., Madeo, F. & Sedej, S. Autophagy in cardiovascular health and disease. *Prog. Mol. Biol. Transl. Sci.* **172**, 87–106 (2020). 32620252 10.1016/bs.pmbts.2020.04.022

[183] Klionsky, D. J. et al. Autophagy in major human diseases. *EMBO J.* **40**, e108863 (2021). 34459017 10.15252/embj.2021108863 PMC8488577

[184] Kaushik, S. et al. Autophagy and the hallmarks of aging. *Ageing Res. Rev.* **72**, 101468 (2021). 34563704 10.1016/j.arr.2021.101468 PMC8616816

[185] Rubinsztein, D. C., Mariño, G. & Kroemer, G. Autophagy and aging. *Cell* **146**, 682–695 (2011). 21884931 10.1016/j.cell.2011.07.030

[186] LaRocca, T. J. et al. Translational evidence that impaired autophagy contributes to arterial ageing. *J. Physiol.* **590**, 3305–3316 (2012). 22570377 10.1113/jphysiol.2012.229690 PMC3459044

[187] Ma, L. et al. Restoring pharmacologic preconditioning in the aging heart: role of mitophagy/autophagy. *J. Gerontol. A Biol. Sci. Med. Sci.* **72**, glw168 (2016). 10.1093/gerona/glw168 27565512

[188] Chang, K. et al. TGFB-INHB/activin signaling regulates age-dependent autophagy and cardiac health through inhibition of MTORC2. *Autophagy* **16**, 1807–1822 (2020). 31884871 10.1080/15548627.2019.1704117 PMC8386626

[189] Cho, J. M. et al. Late-in-life treadmill training rejuvenates autophagy, protein aggregate clearance, and function in mouse hearts. *Aging Cell* **20**, e13467 (2021). 34554626 10.1111/acel.13467 PMC8520717

[190] Abdellatif, M. Hallmarks of cardiovascular ageing. *Nat. Rev. Cardiol.* **20**, 754–777 (2023). 37193857 10.1038/s41569-023-00881-3

[191] Kane, A. E. & Sinclair, D. A. Sirtuins and NAD^+^ in the development and treatment of metabolic and cardiovascular diseases. *Circ. Res.* **123**, 868–885 (2018). 30355082 10.1161/CIRCRESAHA.118.312498 PMC6206880

[192] Pietrocola, F., Galluzzi, L., Bravo-San Pedro, J. M., Madeo, F. & Kroemer, G. Acetyl coenzyme A: a central metabolite and second messenger. *Cell Metab.* **21**, 805–821 (2015). 26039447 10.1016/j.cmet.2015.05.014

[193] Harraz, O. F. & Jensen, L. J. Vascular calcium signalling and ageing. *J. Physiol.* **599**, 5361–5377 (2021). 34705288 10.1113/JP280950 PMC9002240

[194] Shaikh, S. et al. Regulation of cardiomyocyte autophagy by calcium. *Am. J. Physiol. Endocrinol. Metab.* **310**, E587–E596 (2016). 26884385 10.1152/ajpendo.00374.2015 PMC4835942

[195] Behringer, E. J. & Segal, S. S. Impact of aging on calcium signaling and membrane potential in endothelium of resistance arteries: a role for mitochondria. *J. Gerontol. A Biol. Sci. Med. Sci.* **72**, 1627–1637 (2017). 28510636 10.1093/gerona/glx079 PMC5861896

[196] Pucciarelli, S. et al. Spermidine and spermine are enriched in whole blood of nona/centenarians. *Rejuvenation Res.* **15**, 590–595 (2012). 22950434 10.1089/rej.2012.1349

[197] Wang, J. et al. Spermidine alleviates cardiac aging by improving mitochondrial biogenesis and function. *Aging* **12**, 650–671 (2020). 31907336 10.18632/aging.102647 PMC6977682

[198] Madeo, F., Eisenberg, T., Pietrocola, F. & Kroemer, G. Spermidine in health and disease. *Science* **359**, eaan2788 (2018). 29371440 10.1126/science.aan2788

[199] Eisenberg, T. et al. Cardioprotection and lifespan extension by the natural polyamine spermidine. *Nat. Med.* **22**, 1428–1438 (2016). 27841876 10.1038/nm.4222 PMC5806691

[200] LaRocca, T. J., Gioscia-Ryan, R. A., Hearon, C. M. & Seals, D. R. The autophagy enhancer spermidine reverses arterial aging. *Mech. Ageing Dev.* **134**, 314–320 (2013). 23612189 10.1016/j.mad.2013.04.004 PMC3700669

[201] Venkatasubramanian, R., Mahoney, S. A. & Clayton, Z. S. Could angiotensin-II induced T-cell senescence exacerbate age-related vascular dysfunction?. *J. Physiol.* **600**, 1821–1823 (2022). 35238408 10.1113/JP282581 PMC9012694

[202] Coelho-Lima, J. & Spyridopoulos, I. Non-coding RNA regulation of T cell biology: implications for age-associated cardiovascular diseases. *Exp. Gerontol.* **109**, 38–46 (2018). 28652179 10.1016/j.exger.2017.06.014

[203] Zhang, S., Chen, R., Chakrabarti, S. & Su, Z. Resident macrophages as potential therapeutic targets for cardiac ageing and injury. *Clin. Transl. Immunol.* **9**, e1167 (2020). 10.1002/cti2.1167 PMC7450172 32874584

[204] Bruegmann, T. & Smith, G. L. Macrophages: new players in cardiac ageing?. *Cardiovasc. Res.* **114**, e47–e49 (2018). 29800376 10.1093/cvr/cvy047

[205] Spyridopoulos, I. et al. Accelerated telomere shortening in leukocyte subpopulations of patients with coronary heart disease: role of cytomegalovirus seropositivity. *Circulation* **120**, 1364–1372 (2009). 19770396 10.1161/CIRCULATIONAHA.109.854299

[206] Covre, L. P., De Maeyer, R. P. H., Gomes, D. C. O. & Akbar, A. N. The role of senescent T cells in immunopathology. *Aging Cell* **19**, e13272 (2020). 33166035 10.1111/acel.13272 PMC7744956

[207] Anderson, R. et al. Length-independent telomere damage drives post-mitotic cardiomyocyte senescence. *EMBO J* **38**, e100492 (2019). 30737259 10.15252/embj.2018100492 PMC6396144

[208] Mehdizadeh, M., Aguilar, M., Thorin, E., Ferbeyre, G. & Nattel, S. The role of cellular senescence in cardiac disease: Basic biology and clinical relevance. *Nat. Rev. Cardiol.* **19**, 250–264 (2022). 34667279 10.1038/s41569-021-00624-2

[209] Sheydina, A., Riordon, D. R. & Boheler, K. R. Molecular mechanisms of cardiomyocyte aging. *Clin. Sci.* **121**, 315–329 (2011). 10.1042/CS20110115 PMC5928524 21699498

[210] Papp, Z., Czuriga, D., Balogh, L., Balogh, Á & Borbély, A. How cardiomyocytes make the heart old. *Curr. Pharm. Biotechnol.* **13**, 2515–2521 (2012). 22280426

[211] Kajstura, J. et al. Telomere shortening is an in vivo marker of myocyte replication and aging. *Am. J. Pathol.* **156**, 813–819 (2000). 10702397 10.1016/S0002-9440(10)64949-8 PMC1876843

[212] Hall, B. M. et al. Aging of mice is associated with p16(Ink4a)- and β-galactosidase-positive macrophage accumulation that can be induced in young mice by senescent cells. *Aging* **8**, 1294–1315 (2016). 27391570 10.18632/aging.100991 PMC4993332

[213] Campisi, J. Cellular senescence: putting the paradoxes in perspective. *Curr. Opin. Genet. Dev.* **21**, 107–112 (2011). 21093253 10.1016/j.gde.2010.10.005 PMC3073609

[214] Siddiqi, S. & Sussman, M. A. Cardiac hegemony of senescence. *Curr. Transl. Geriatr. and Exp. Gerontol. Rep.* **2**, 247–254 (2013). 10.1007/s13670-013-0064-3 PMC3862253 24349878

[215] Childs, B. G., Durik, M., Baker, D. J. & Van Deursen, J. M. Cellular senescence in aging and age-related disease: from mechanisms to therapy. *Nat. Med.* **21**, 1424–1435 (2015). 26646499 10.1038/nm.4000 PMC4748967

[216] McHugh, D. & Gil, J. Senescence and aging: causes, consequences, and therapeutic avenues. *J. Cell Biol.* **217**, 65–77 (2018). 29114066 10.1083/jcb.201708092 PMC5748990

[217] Biernacka, A. & Frangogiannis, N. G. Aging and cardiac fibrosis. *Aging Dis* **2**, 158–173 (2011). 21837283 PMC3153299

[218] Riquelme, J. A. et al. Increased production of functional small extracellular vesicles in senescent endothelial cells. *J. Cell. Mol. Med.* **24**, 4871–4876 (2020). 32101370 10.1111/jcmm.15047 PMC7176858

[219] Riches-Suman, K. & Hussain, A. Identifying and targeting the molecular signature of smooth muscle cells undergoing early vascular ageing. *Biochim. Biophys. Acta Mol. Basis Dis.* **1868**, 166403 (2022). 35367337 10.1016/j.bbadis.2022.166403

[220] Rota, M., Goichberg, P., Anversa, P. & Leri, A. Aging effects on cardiac progenitor cell physiology. *Compr. Physiol.* **5**, 1775–1814 (2015). 26426466 10.1002/cphy.c140082

[221] Cesselli, D. et al. Effects of age and heart failure on human cardiac stem cell function. *Am. J. Pathol.* **179**, 349–366 (2011). 21703415 10.1016/j.ajpath.2011.03.036 PMC3175070

[222] Hariharan, N. & Sussman, M. A. Cardiac aging—getting to the stem of the problem. *J. Mol. Cell. Cardiol.* **83**, 32–36 (2015). 25886698 10.1016/j.yjmcc.2015.04.008 PMC4459886

[223] Allsopp, R. C., Morin, G. B., DePinho, R., Harley, C. B. & Weissman, I. L. Telomerase is required to slow telomere shortening and extend replicative lifespan of HSCs during serial transplantation. *Blood* **102**, 517–520 (2003). 12663456 10.1182/blood-2002-07-2334

[224] Nollet, E. et al. Accelerated cellular senescence as underlying mechanism for functionally impaired bone marrow-derived progenitor cells in ischemic heart disease. *Atherosclerosis* **260**, 138–146 (2017). 28434530 10.1016/j.atherosclerosis.2017.03.023

[225] Gorgoulis, V. et al. Cellular senescence: defining a path forward. *Cell* **179**, 813–827 (2019). 31675495 10.1016/j.cell.2019.10.005

[226] Sano, M. et al. p53-induced inhibition of hif-1 causes cardiac dysfunction during pressure overload. *Nature* **446**, 444–448 (2007). 17334357 10.1038/nature05602

[227] Gogiraju, R. et al. Endothelial p53 deletion improves angiogenesis and prevents cardiac fibrosis and heart failure induced by pressure overload in mice. *J. Am. Heart Assoc.* **4**, e001770 (2015). 25713289 10.1161/JAHA.115.001770 PMC4345879

[228] Yoshida, Y. et al. p53-induced inflammation exacerbates cardiac dysfunction during pressure overload. *J. Mol. Cell. Cardiol.* **85**, 183–198 (2015). 26055447 10.1016/j.yjmcc.2015.06.001

[229] el-Deiry, W. S. et al. WAF1, a potential mediator of p53 tumor suppression. *Cell* **75**, 817–825 (1993). 8242752 10.1016/0092-8674(93)90500-p

[230] Spyridopoulos, I., Isner, J. M. & Losordo, D. W. Oncogenic RAS induces premature senescence in endothelial cells: role of p21(Cip1/Waf1). *Basic Res. Cardiol.* **97**, 117–124 (2002). 12002258 10.1007/s003950200001

[231] Wu, L. et al. p53 mediated transcription of omi/HtrA2 in aging myocardium. *Biochem. Biophys. Res. Commun.* **519**, 734–739 (2019). 31543347 10.1016/j.bbrc.2019.09.062

[232] Lee, C.-L. et al. p53 functions in endothelial cells to prevent radiation-induced myocardial injury in mice. *Sci. Signal* **5**, ra52 (2012). 22827996 10.1126/scisignal.2002918 PMC3533440

[233] Rossman, M. J. et al. Endothelial cell senescence with aging in healthy humans: prevention by habitual exercise and relation to vascular endothelial function. *Am. J. Physiol. Circ. Physiol.* **313**, H890–H895 (2017). 10.1152/ajpheart.00416.2017 PMC5792201 28971843

[234] Lewis-McDougall, F. C. et al. Aged-senescent cells contribute to impaired heart regeneration. *Aging Cell* **18**, e12931 (2019). 30854802 10.1111/acel.12931 PMC6516154

[235] Zhao, H. et al. Identifying specific functional roles for senescence across cell types. *Cell* **187**, 7314–7334.e21 (2024). 39368477 10.1016/j.cell.2024.09.021

[236] Lázár, E., Sadek, H. A. & Bergmann, O. Cardiomyocyte renewal in the human heart: insights from the fall-out. *Eur. Heart J.* **38**, 2333–2342 (2017). 28810672 10.1093/eurheartj/ehx343 PMC5837331

[237] Bergmann, O. et al. Dynamics of cell generation and turnover in the human heart. *Cell* **161**, 1566–1575 (2015). 26073943 10.1016/j.cell.2015.05.026

[238] Sun, S. et al. CD133+ endothelial-like stem cells restore neovascularization and promote longevity in progeroid and naturally aged mice. *Nat. Aging* **3**, 1401–1414 (2023). 37946040 10.1038/s43587-023-00512-z PMC10645602

[239] Tang, J. et al. Arterial Sca1+ vascular stem cells generate de novo smooth muscle for artery repair and regeneration. *Cell Stem Cell* **26**, 81–96.e4 (2020). 31883835 10.1016/j.stem.2019.11.010

[240] Feng, R. et al. Stem cell-derived extracellular vesicles mitigate ageing-associated arterial stiffness and hypertension. *J. Extracell. Vesicles* **9**, 1783869 (2020). 32939234 10.1080/20013078.2020.1783869 PMC7480600

[241] Barzegar, M. et al. Human placenta mesenchymal stem cell protection in ischemic stroke is angiotensin converting enzyme-2 and Masr receptor-dependent. *Stem Cells* **39**, 1335–1348 (2021). 34124808 10.1002/stem.3426 PMC8881785

[242] Ohta, H., Liu, X. & Maeda, M. Autologous adipose mesenchymal stem cell administration in arteriosclerosis and potential for anti-aging application: a retrospective cohort study. *Stem Cell Res. Ther.* **11**, 538 (2020). 33308301 10.1186/s13287-020-02067-x PMC7733281

[243] Holm, A., Heumann, T. & Augustin, H. G. Microvascular mural cell organotypic heterogeneity and functional plasticity. *Trends Cell Biol* **28**, 302–316 (2018). 29307447 10.1016/j.tcb.2017.12.002

[244] Bergers, G. & Song, S. The role of pericytes in blood-vessel formation and maintenance. *NEUONC* **7**, 452–464 (2005). 10.1215/S1152851705000232 PMC1871727 16212810

[245] Shibahara, T. et al. Pericyte-mediated tissue repair through PDGFRβ promotes peri-infarct astrogliosis, oligodendrogenesis, and functional recovery after acute ischemic stroke. *eNeuro* **7**, (2020). 10.1523/ENEURO.0474-19.2020 PMC7070447 32046974

[246] Daniel, J.-M. et al. Regulator of G-protein signaling 5 prevents smooth muscle cell proliferation and attenuates neointima formation. *Arterioscler. Thromb. Vasc. Biol.* **36**, 317–327 (2016). 26663397 10.1161/ATVBAHA.115.305974

[247] Gunaje, J. J., Bahrami, A. J., Schwartz, S. M., Daum, G. & Mahoney, W. M. PDGF-dependent regulation of regulator of G protein signaling-5 expression and vascular smooth muscle cell functionality. *Am. J. Physiol. Cell Physiol.* **301**, C478–C489 (2011). 21593453 10.1152/ajpcell.00348.2010 PMC3154557

[248] Tamiato, A. et al. Age-dependent RGS5 loss in pericytes induces cardiac dysfunction and fibrosis. *Circ. Res.* **134**, 1240–1255 (2024). 38563133 10.1161/CIRCRESAHA.123.324183 PMC11081481

[249] Clevers, H. & Watt, F. M. Defining adult stem cells by function, not by phenotype. *Annu. Rev. Biochem.* **87**, 1015–1027 (2018). 29494240 10.1146/annurev-biochem-062917-012341

[250] Ratajczak, M. Z., Machalinski, B., Wojakowski, W., Ratajczak, J. & Kucia, M. A hypothesis for an embryonic origin of pluripotent oct-4(+) stem cells in adult bone marrow and other tissues. *Leukemia* **21**, 860–867 (2007). 17344915 10.1038/sj.leu.2404630

[251] Takahashi, K. & Yamanaka, S. Induction of pluripotent stem cells from mouse embryonic and adult fibroblast cultures by defined factors. *Cell* **126**, 663–676 (2006). 16904174 10.1016/j.cell.2006.07.024

[252] Ocampo, A. et al. In vivo amelioration of age-associated hallmarks by partial reprogramming. *Cell* **167**, 1719–1733.e12 (2016). 27984723 10.1016/j.cell.2016.11.052 PMC5679279

[253] Chen, Y. et al. Reversible reprogramming of cardiomyocytes to a fetal state drives heart regeneration in mice. *Science* **373**, 1537–1540 (2021). 34554778 10.1126/science.abg5159

[254] Sabbatinelli, J. et al. Where metabolism meets senescence: focus on endothelial cells. *Front. Physiol.* **10**, 1523 (2019). 31920721 10.3389/fphys.2019.01523 PMC6930181

[255] Mroueh, A. et al. SGLT2 expression in human vasculature and heart correlates with low-grade inflammation and causes eNOS-NO/ROS imbalance. *Cardiovasc. Res.* **121**, 643–657 (2025). 39739876 10.1093/cvr/cvae257

[256] Clayton, Z. S. et al. Cellular senescence contributes to large elastic artery stiffening and endothelial dysfunction with aging: amelioration with senolytic treatment. *Hypertension* **80**, 2072–2087 (2023). 37593877 10.1161/HYPERTENSIONAHA.123.21392 PMC10530538

[257] Giuliani, A. et al. Senescent endothelial cells sustain their senescence-associated secretory phenotype (SASP) through enhanced fatty acid oxidation. *Antioxidants* **12**, 1956 (2023). 38001810 10.3390/antiox12111956 PMC10668971

[258] Ma, T. K., Kam, K. K., Yan, B. P. & Lam, Y. Renin–angiotensin–aldosterone system blockade for cardiovascular diseases: current status. *Br. J. Pharmacol.* **160**, 1273–1292 (2010). 20590619 10.1111/j.1476-5381.2010.00750.x PMC2938800

[259] Jia, G., Aroor, A. R., Hill, M. A. & Sowers, J. R. Role of renin-angiotensin-aldosterone system activation in promoting cardiovascular fibrosis and stiffness. *Hypertension* **72**, 537–548 (2018). 29987104 10.1161/HYPERTENSIONAHA.118.11065 PMC6202147

[260] Conti, S., Cassis, P. & Benigni, A. Aging and the renin-angiotensin system. *Hypertension* **60**, 878–883 (2012). 22926952 10.1161/HYPERTENSIONAHA.110.155895

[261] Dai, D.-F. et al. Overexpression of catalase targeted to mitochondria attenuates murine cardiac aging. *Circulation* **119**, 2789–2797 (2009). 19451351 10.1161/CIRCULATIONAHA.108.822403 PMC2858759

[262] Dinh, Q. N. et al. Pressor response to angiotensin II is enhanced in aged mice and associated with inflammation, vasoconstriction and oxidative stress. *Aging* **9**, 1595–1606 (2017). 28659507 10.18632/aging.101255 PMC5509458

[263] Krug, A. W. et al. Elevated mineralocorticoid receptor activity in aged rat vascular smooth muscle cells promotes a proinflammatory phenotype via extracellular signal-regulated kinase 1/2 mitogen-activated protein kinase and epidermal growth factor receptor-dependent pathways. *Hypertension* **55**, 1476–1483 (2010). 20421514 10.1161/HYPERTENSIONAHA.109.148783 PMC2883813

[264] DuPont, J. J. et al. Vascular mineralocorticoid receptor regulates microRNA-155 to promote vasoconstriction and rising blood pressure with aging. *J. Clin. Investig. Insight* **1**, e88942 (2016). 10.1172/jci.insight.88942 PMC5033871 27683672

[265] McCurley, A. et al. Direct regulation of blood pressure by smooth muscle cell mineralocorticoid receptors. *Nat. Med.* **18**, 1429–1433 (2012). 22922412 10.1038/nm.2891 PMC3491085

[266] Kim, S. K. et al. Smooth muscle cell-mineralocorticoid receptor as a mediator of cardiovascular stiffness with aging. *Hypertension* **71**, 609–621 (2018). 29463624 10.1161/HYPERTENSIONAHA.117.10437 PMC5843545

[267] Harvey, A., Montezano, A. C., Lopes, R. A., Rios, F. & Touyz, R. M. Vascular fibrosis in aging and hypertension: molecular mechanisms and clinical implications. *Can. J. Cardiol.* **32**, 659–668 (2016). 27118293 10.1016/j.cjca.2016.02.070 PMC4906153

[268] Hu, J.-R., Abdullah, A., Nanna, M. G. & Soufer, R. The brain-heart axis: neuroinflammatory interactions in cardiovascular disease. *Curr. Cardiol. Rep.* **25**, 1745–1758 (2023). 37994952 10.1007/s11886-023-01990-8 PMC10908342

[269] Lymperopoulos, A., Rengo, G. & Koch, W. J. Adrenergic nervous system in heart failure: pathophysiology and therapy. *Circ. Res.* **113**, 739–753 (2013). 23989716 10.1161/CIRCRESAHA.113.300308 PMC3843360

[270] Elia, A. et al. Aging is associated with cardiac autonomic nerve fiber depletion and reduced cardiac and circulating BDNF levels. *J. Geriatr. Cardiol.* **18**, 549–559 (2021). 34404991 10.11909/j.issn.1671-5411.2021.07.009 PMC8352776

[271] Ferrara, N. et al. -adrenergic receptor responsiveness in aging heart and clinical implications. *Front. Physiol.* **4**, 396 (2014). 24409150 10.3389/fphys.2013.00396 PMC3885807

[272] Xiao, R. P., Spurgeon, H. A., O’Connor, F. & Lakatta, E. G. Age-associated changes in beta-adrenergic modulation on rat cardiac excitation-contraction coupling. *J. Clin. Investig.* **94**, 2051–2059 (1994). 7962551 10.1172/JCI117559 PMC294640

[273] Stratton, J. R. et al. Differences in cardiovascular responses to isoproterenol in relation to age and exercise training in healthy men. *Circulation* **86**, 504–512 (1992). 1638718 10.1161/01.cir.86.2.504

[274] Engelhardt, S., Hein, L., Wiesmann, F. & Lohse, M. J. Progressive hypertrophy and heart failure in beta1-adrenergic receptor transgenic mice. *Proc. Natl. Acad. Sci. USA* **96**, 7059–7064 (1999). 10359838 10.1073/pnas.96.12.7059 PMC22055

[275] Ziff, O. J. et al. Beta-blocker efficacy across different cardiovascular indications: an umbrella review and meta-analytic assessment. *BMC Med.* **18**, 103 (2020). 32366251 10.1186/s12916-020-01564-3 PMC7199339

[276] Sipahi, I. et al. Beta-blockers and progression of coronary atherosclerosis: pooled analysis of 4 intravascular ultrasonography trials. *Ann. Intern. Med.* **147**, 10–18 (2007). 17606956 10.7326/0003-4819-147-1-200707030-00003

[277] Santulli, G. & Iaccarino, G. Adrenergic signaling in heart failure and cardiovascular aging. *Maturitas* **93**, 65–72 (2016). 27062709 10.1016/j.maturitas.2016.03.022 PMC5036981

[278] Santulli, G. et al. CaMK4 gene deletion induces hypertension. *J. Am. Heart Assoc.* **1**, e001081 (2012). 23130158 10.1161/JAHA.112.001081 PMC3487344

[279] Santulli, G. Coronary heart disease risk factors and mortality. *J. Am. Heart Assoc*. **307**, (2012). 10.1001/jama.2012.323 22436947

[280] Vu, T. T. et al. Prospective relationship of low cardiovascular risk factor profile at younger ages to ankle-brachial index: 39-year follow-up—the Chicago Healthy Aging study. *J. Am. Heart Assoc.* **1**, e001545 (2012). 23316312 10.1161/JAHA.112.001545 PMC3540658

[281] Ciccarelli, M. et al. The possible role of chromosome X variability in hypertensive familiarity. *J. Hum. Hypertens.* **31**, 37–42 (2017). 26911533 10.1038/jhh.2016.9 PMC4999347

[282] Galasso, G. et al. 2-adrenergic receptor stimulation improves endothelial progenitor cell-mediated ischemic neoangiogenesis. *Circ. Res.* **112**, 1026–1034 (2013). 23418295 10.1161/CIRCRESAHA.111.300152

[283] Ciccarelli, M. et al. Impaired neoangiogenesis in β₂-adrenoceptor gene-deficient mice: restoration by intravascular human β₂-adrenoceptor gene transfer and role of NFκB and CREB transcription factors. *Br. J. Pharmacol.* **162**, 712–721 (2011). 20958287 10.1111/j.1476-5381.2010.01078.x PMC3041259

[284] Chisalita, S. I. & Arnqvist, H. J. Insulin-like growth factor I receptors are more abundant than insulin receptors in human micro- and macrovascular endothelial cells. *Am. J. Physiol. Endocrinol. Metab.* **286**, E896–E901 (2004). 14722023 10.1152/ajpendo.00327.2003

[285] Chisalita, S. I., Johansson, G. S., Liefvendahl, E., Bäck, K. & Arnqvist, H. J. Human aortic smooth muscle cells are insulin resistant at the receptor level but sensitive to IGF1 and IGF2. *J. Mol. Endocrinol.* **43**, 231–239 (2009). 19589910 10.1677/JME-09-0021

[286] Johansson, G. S., Chisalita, S. I. & Arnqvist, H. J. Human microvascular endothelial cells are sensitive to IGF-I but resistant to insulin at the receptor level. *Mol. Cell. Endocrinol.* **296**, 58–63 (2008). 18708119 10.1016/j.mce.2008.07.012

[287] Khan, A. S., Sane, D. C., Wannenburg, T. & Sonntag, W. E. Growth hormone, insulin-like growth factor-1 and the aging cardiovascular system. *Cardiovasc. Res.* **54**, 25–35 (2002). 12062358 10.1016/s0008-6363(01)00533-8

[288] Ungvari, Z. & Csiszar, A. The Emerging Role of IGF-1 deficiency in cardiovascular aging: recent advances. *J. Gerontol. A Biol. Sci. Med. Sci.* **67A**, 599–610 (2012). 10.1093/gerona/gls072 PMC3348495 22451468

[289] Sonntag, W. E., Lynch, C. D., Cooney, P. T. & Hutchins, P. M. Decreases in cerebral microvasculature with age are associated with the decline in growth hormone and insulin-like growth factor 1. *Endocrinology* **138**, 3515–3520 (1997). 9231806 10.1210/endo.138.8.5330

[290] Hua, K., Forbes, M. E., Lichtenwalner, R. J., Sonntag, W. E. & Riddle, D. R. Adult-onset deficiency in growth hormone and insulin-like growth factor-I alters oligodendrocyte turnover in the corpus callosum. *Glia* **57**, 1062–1071 (2009). 19115393 10.1002/glia.20829 PMC2696576

[291] Sonntag, W. E., Ramsey, M. & Carter, C. S. Growth hormone and insulin-like growth factor-1 (IGF-1) and their influence on cognitive aging. *Ageing Res. Rev.* **4**, 195–212 (2005). 16024298 10.1016/j.arr.2005.02.001

[292] Ramsey, M. M., Weiner, J. L., Moore, T. P., Carter, C. S. & Sonntag, W. E. Growth hormone treatment attenuates age-related changes in hippocampal short-term plasticity and spatial learning. *Neuroscience* **129**, 119–127 (2004). 15489035 10.1016/j.neuroscience.2004.08.001

[293] Poe, B. H., Linville, C., Riddle, D. R., Sonntag, W. E. & Brunso-Bechtold, J. K. Effects of age and insulin-like growth factor-1 on neuron and synapse numbers in area CA3 of hippocampus. *Neuroscience* **107**, 231–238 (2001). 11731097 10.1016/s0306-4522(01)00341-4

[294] Lichtenwalner, R. J. et al. Intracerebroventricular infusion of insulin-like growth factor-I ameliorates the age-related decline in hippocampal neurogenesis. *Neuroscience* **107**, 603–613 (2001). 11720784 10.1016/s0306-4522(01)00378-5

[295] Lopez-Lopez, C., LeRoith, D. & Torres-Aleman, I. Insulin-like growth factor I is required for vessel remodeling in the adult brain. *Proc. Natl. Acad. Sci. USA* **101**, 9833–9838 (2004). 15210967 10.1073/pnas.0400337101 PMC470760

[296] Khan, A. S., Lynch, C. D., Sane, D. C., Willingham, M. C. & Sonntag, W. E. Growth hormone increases regional coronary blood flow and capillary density in aged rats. *J. Gerontol. A Biol. Sci. Med. Sci.* **56**, B364–B371 (2001). 11487595 10.1093/gerona/56.8.b364

[297] Granger, D. & Granger, J. P. *Colloquium Series on Integrated Systems Physiology: From Molecule to Function to Disease* (Morgan & Claypool Life Sciences, 2011).

[298] Rivard, A. et al. Age-dependent impairment of angiogenesis. *Circulation* **99**, 111–120 (1999). 9884387 10.1161/01.cir.99.1.111

[299] Ungvari, Z. et al. Endothelial dysfunction and angiogenesis impairment in the ageing vasculature. *Nat. Rev. Cardiol.* **15**, 555–565 (2018). 29795441 10.1038/s41569-018-0030-z PMC6612360

[300] Pérez-Gutiérrez, L. & Ferrara, N. Biology and therapeutic targeting of vascular endothelial growth factor a. *Nat. Rev. Mol. Cell Biol.* **24**, 816–834 (2023). 37491579 10.1038/s41580-023-00631-w

[301] Gogiraju, R., Bochenek, M. L. & Schäfer, K. Angiogenic endothelial cell signaling in cardiac hypertrophy and heart failure. *Front. Cardiovasc. Med.* **6**, 20 (2019). 30895179 10.3389/fcvm.2019.00020 PMC6415587

[302] Grunewald, M. et al. Counteracting age-related VEGF signaling insufficiency promotes healthy aging and extends life span. *Science* **373**, eabc8479 (2021). 34326210 10.1126/science.abc8479

[303] Wang, L. et al. Integrin-YAP/TAZ-JNK cascade mediates atheroprotective effect of unidirectional shear flow. *Nature* **540**, 579–582 (2016). 27926730 10.1038/nature20602

[304] Sladitschek-Martens, H. L. et al. YAP/TAZ activity in stromal cells prevents ageing by controlling cGAS-STING. *Nature* **607**, 790–798 (2022). 35768505 10.1038/s41586-022-04924-6 PMC7613988

[305] Admasu, T. D., Rae, M. & Stolzing, A. Dissecting primary and secondary senescence to enable new senotherapeutic strategies. *Ageing Res. Rev.* **70**, 101412 (2021). 34302996 10.1016/j.arr.2021.101412

[306] Yang, H., Wang, H., Ren, J., Chen, Q. & Chen, Z. J. cGAS is essential for cellular senescence. *Proc. Natl. Acad. Sci. USA* **114**, E4612–E4620 (2017). 28533362 10.1073/pnas.1705499114 PMC5468617

[307] Meyer, K., López-Domínguez, J. A., Maus, M., Kovatcheva, M. & Serrano, M. Senescence as a therapeutic target: Current state and future challenges. *Cell. Senescence Dis.* 425–442 (2022).

[308] Jaiswal, S. & Ebert, B. L. Clonal hematopoiesis in human aging and disease. *Science* **366**, eaan4673 (2019). 31672865 10.1126/science.aan4673 PMC8050831

[309] Cheng, L.-Q., Zhang, Z.-Q., Chen, H.-Z. & Liu, D.-P. Epigenetic regulation in cell senescence. *J. Mol. Med.* **95**, 1257–1268 (2017). 28887637 10.1007/s00109-017-1581-x

[310] Katsyuba, E., Romani, M., Hofer, D. & Auwerx, J. NAD+ homeostasis in health and disease. *Nat. Metab.* **2**, 9–31 (2020). 32694684 10.1038/s42255-019-0161-5

[311] Kulkarni, A. S., Gubbi, S. & Barzilai, N. Benefits of metformin in attenuating the hallmarks of aging. *Cell Metab* **32**, 15–30 (2020). 32333835 10.1016/j.cmet.2020.04.001 PMC7347426

[312] Triposkiadis, F. et al. The continuous heart failure spectrum: moving beyond an ejection fraction classification. *Eur. Heart J.* **40**, 2155–2163 (2019). 30957868 10.1093/eurheartj/ehz158 PMC7963129

[313] Lakatta, E. G. So! What’s aging? Is cardiovascular aging a disease?. *J. Mol. Cell. Cardiol.* **83**, 1–13 (2015). 25870157 10.1016/j.yjmcc.2015.04.005 PMC4532266

[314] Ma, S., Wang, Y., Chen, Y. & Cao, F. The role of the autophagy in myocardial ischemia/reperfusion injury. *Biochim. Biophys. Acta Mol. Basis Dis.* **1852**, 271–276 (2015). 10.1016/j.bbadis.2014.05.010 24859226

[315] Tannous, P. et al. Intracellular protein aggregation is a proximal trigger of cardiomyocyte autophagy. *Circulation* **117**, 3070–3078 (2008). 18541737 10.1161/CIRCULATIONAHA.107.763870 PMC2601596

[316] Martín-Fernández, B. & Gredilla, R. Estrés oxidativo mitocondrial y envejecimiento cardíaco. *Clín. Investig. Art.* **30**, 74–83 (2018). 10.1016/j.arteri.2017.12.002 29398015

[317] Berkowicz, P. et al. Accelerated ageing and coronary microvascular dysfunction in chronic heart failure in Tgαq*44 mice. *GeroScience* **45**, 1619–1648 (2023). 36692592 10.1007/s11357-022-00716-y PMC10400753

[318] Gevaert, A. B. et al. Endothelial senescence contributes to heart failure with preserved ejection fraction in an aging mouse model. *Circ. Heart Fail.* **10**, e003806 (2017). 28611124 10.1161/CIRCHEARTFAILURE.116.003806

[319] Libby, P. The changing landscape of atherosclerosis. *Nature* **592**, 524–533 (2021). 33883728 10.1038/s41586-021-03392-8

[320] Minamino, T. et al. Endothelial cell senescence in human atherosclerosis: role of telomere in endothelial dysfunction. *Circulation* **105**, 1541–1544 (2002). 11927518 10.1161/01.cir.0000013836.85741.17

[321] Xiang, Q. et al. New insight into dyslipidemia-induced cellular senescence in atherosclerosis. *Biol. Rev.* **97**, 1844–1867 (2022). 35569818 10.1111/brv.12866 PMC9541442

[322] Stojanović, S. D., Fiedler, J., Bauersachs, J., Thum, T. & Sedding, D. G. Senescence-induced inflammation: an important player and key therapeutic target in atherosclerosis. *Eur. Heart J.* **41**, 2983–2996 (2020). 31898722 10.1093/eurheartj/ehz919 PMC7453834

[323] Caland, L. et al. Knockdown of angiopoietin-like 2 induces clearance of vascular endothelial senescent cells by apoptosis, promotes endothelial repair and slows atherogenesis in mice. *Aging* **11**, 3832–3850 (2019). 31186381 10.18632/aging.102020 PMC6594793

[324] Grootaert, M. O. J. et al. Vascular smooth muscle cell death, autophagy and senescence in atherosclerosis. *Cardiovasc. Res.* **114**, 622–634 (2018). 29360955 10.1093/cvr/cvy007

[325] Grootaert, M. O. J., Finigan, A., Figg, N. L., Uryga, A. K. & Bennett, M. R. SIRT6 protects smooth muscle cells from senescence and reduces atherosclerosis. *Circ. Res.* **128**, 474–491 (2021). 33353368 10.1161/CIRCRESAHA.120.318353 PMC7899748

[326] Li, X. et al. TRAP1 drives smooth muscle cell senescence and promotes atherosclerosis via HDAC3-primed histone H4 lysine 12 lactylation. *Eur. Heart J.* **45**, 4219–4235 (2024). 39088352 10.1093/eurheartj/ehae379 PMC11481199

[327] Childs, B. G. et al. Senescent intimal foam cells are deleterious at all stages of atherosclerosis. *Science* **354**, 472–477 (2016). 27789842 10.1126/science.aaf6659 PMC5112585

[328] Vellasamy, D. M. et al. Targeting immune senescence in atherosclerosis. *Int. J. Mol. Sci.* **23**, 13059 (2022). 36361845 10.3390/ijms232113059 PMC9658319

[329] Milewicz, D. M. & Ramirez, F. Therapies for thoracic aortic aneurysms and acute aortic dissections: old controversies and new opportunities. *Arterioscler., Thromb., Vasc. Biol.* **39**, 126–136 (2019). 30651002 10.1161/ATVBAHA.118.310956 PMC6398943

[330] Tyrrell, D. J. et al. Aging alters the aortic proteome in health and thoracic aortic aneurysm. *Arterioscler. Thromb. Vasc. Biol.* **42**, 1060–1076 (2022). 35510553 10.1161/ATVBAHA.122.317643 PMC9339483

[331] Liu, Z.-L. et al. Aging aggravates aortic aneurysm and dissection via miR-1204-MYLK signaling axis in mice. *Nat. Commun.* **15**, 5985 (2024). 39013850 10.1038/s41467-024-50036-2 PMC11252124

[332] Chen, H.-Z. et al. Age-associated sirtuin 1 reduction in vascular smooth muscle links vascular senescence and inflammation to abdominal aortic aneurysm. *Circ. Res.* **119**, 1076–1088 (2016). 27650558 10.1161/CIRCRESAHA.116.308895 PMC6546422

[333] Yang, L. et al. SIRT6-mediated vascular smooth muscle cells senescence participates in the pathogenesis of abdominal aortic aneurysm. *Atherosclerosis* **392**, 117483 (2024). 38490134 10.1016/j.atherosclerosis.2024.117483

[334] Gao, P. et al. MKL1 cooperates with p38MAPK to promote vascular senescence, inflammation, and abdominal aortic aneurysm. *Redox Biol* **41**, 101903 (2021). 33667992 10.1016/j.redox.2021.101903 PMC7937568

[335] Xie, J. et al. Clearance of stress-induced premature senescent cells alleviates the formation of abdominal aortic aneurysms. *Aging Dis* **14**, 1778 (2023). 37196124 10.14336/AD.2023.0215 PMC10529745

[336] Boland, B. et al. Promoting the clearance of neurotoxic proteins in neurodegenerative disorders of ageing. *Nat. Rev. Drug Discov.* **17**, 660–688 (2018). 30116051 10.1038/nrd.2018.109 PMC6456907

[337] Soraci, L. et al. Neuroinflammaging: a tight line between normal aging and age-related neurodegenerative disorders. *Aging Dis* **15**, 1726–1747 (2024). 38300639 10.14336/AD.2023.1001 PMC11272206

[338] Banks, W. A., Reed, M. J., Logsdon, A. F., Rhea, E. M. & Erickson, M. A. Healthy aging and the blood–brain barrier. *Nat. Aging* **1**, 243–254 (2021). 34368785 10.1038/s43587-021-00043-5 PMC8340949

[339] Chow, B. W. & Gu, C. The molecular constituents of the blood–brain barrier. *Trends Neurosci* **38**, 598–608 (2015). 26442694 10.1016/j.tins.2015.08.003 PMC4597316

[340] Sweeney, M. D., Sagare, A. P. & Zlokovic, B. V. Blood–brain barrier breakdown in Alzheimer disease and other neurodegenerative disorders. *Nat. Rev. Neurol.* **14**, 133–150 (2018). 29377008 10.1038/nrneurol.2017.188 PMC5829048

[341] Senatorov, V. V. et al. Blood-brain barrier dysfunction in aging induces hyperactivation of TGFβ signaling and chronic yet reversible neural dysfunction. *Sci. Transl. Med.* **11**, eaaw8283 (2019). 31801886 10.1126/scitranslmed.aaw8283

[342] Kutuzov, N., Flyvbjerg, H. & Lauritzen, M. Contributions of the glycocalyx, endothelium, and extravascular compartment to the blood–brain barrier. *Proc. Natl. Acad. Sci. USA* **115**, E9429–E9438 (2018). 30217895 10.1073/pnas.1802155115 PMC6176561

[343] Shi, S. M. et al. Glycocalyx dysregulation impairs blood–brain barrier in ageing and disease. *Nature* **639**, 985–994 (2025). 40011765 10.1038/s41586-025-08589-9 PMC11946907

[344] Nelson, A. R., Sweeney, M. D., Sagare, A. P. & Zlokovic, B. V. Neurovascular dysfunction and neurodegeneration in dementia and Alzheimer’s disease. *Biochim. Biophys. Acta Mol. Basis Dis.* **1862**, 887–900 (2016). 10.1016/j.bbadis.2015.12.016 PMC4821735 26705676

[345] Van De Haar, H. J. et al. Neurovascular unit impairment in early Alzheimer’s disease measured with magnetic resonance imaging. *Neurobiol. Aging* **45**, 190–196 (2016). 27459939 10.1016/j.neurobiolaging.2016.06.006

[346] Shams, S. & Wahlund, L.-O. Cerebral microbleeds as a biomarker in Alzheimer’S disease? A review in the field. *Biomarkers Med* **10**, 9–18 (2016). 10.2217/bmm.15.101 26641942

[347] Shams, S. et al. Cerebral microbleeds: different prevalence, topography, and risk factors depending on dementia diagnosis—The Karolinska Imaging Dementia Study. *Am. J. Neuroradiol.* **36**, 661–666 (2015). 25523590 10.3174/ajnr.A4176 PMC7964321

[348] Poliakova, T., Levin, O., Arablinskiy, A., Vasenina, E. & Zerr, I. Cerebral microbleeds in early Alzheimer’s disease. *J. Neurol.* **263**, 1961–1968 (2016). 27389080 10.1007/s00415-016-8220-2

[349] Halliday, M. R. et al. Accelerated pericyte degeneration and blood–brain barrier breakdown in apolipoprotein E4 carriers with Alzheimer’s disease. *J. Cereb. Blood Flow Metab.* **36**, 216–227 (2016). 25757756 10.1038/jcbfm.2015.44 PMC4758554

[350] Zhao, Z., Nelson, A. R., Betsholtz, C. & Zlokovic, B. V. Establishment and dysfunction of the blood-brain barrier. *Cell* **163**, 1064–1078 (2015). 26590417 10.1016/j.cell.2015.10.067 PMC4655822

[351] O’Brien, J. T. & Thomas, A. Vascular dementia. *The Lancet* **386**, 1698–1706 (2015). 10.1016/S0140-6736(15)00463-8 26595643

[352] Farrall, A. J. & Wardlaw, J. M. Blood–brain barrier: ageing and microvascular disease – systematic review and meta-analysis. *Neurobiol. Aging* **30**, 337–352 (2009). 17869382 10.1016/j.neurobiolaging.2007.07.015

[353] Akiguchi, I., Tomimoto, H., Suenaga, T., Wakita, H. & Budka, H. Blood-brain barrier dysfunction in Binswanger’s disease; an immunohistochemical study. *Acta Neuropathol* **95**, 78–84 (1998). 9452825 10.1007/s004010050768

[354] Matsuda, K. et al. Measurement of laminins in the cerebrospinal fluid obtained from patients with Alzheimer’s disease and vascular dementia using a modified enzyme-linked immunosorbent assay. *Dementia Geriatr. Cognit. Disord.* **14**, 113–122 (2002). 10.1159/000063601 12218253

[355] Taheri, S. et al. Blood–brain barrier permeability abnormalities in vascular cognitive impairment. *Stroke* **42**, 2158–2163 (2011). 21719768 10.1161/STROKEAHA.110.611731 PMC3584170

[356] Nation, D. A. et al. Blood–brain barrier breakdown is an early biomarker of human cognitive dysfunction. *Nat. Med.* **25**, 270–276 (2019). 30643288 10.1038/s41591-018-0297-y PMC6367058

[357] Chao, Y. X., He, B. P. & Tay, S. S. W. Mesenchymal stem cell transplantation attenuates blood-brain barrier damage and neuroinflammation and protects dopaminergic neurons against MPTP toxicity in the substantia nigra in a model of Parkinson’s disease. *J. Neuroimmunol.* **216**, 39–50 (2009). 19819031 10.1016/j.jneuroim.2009.09.003

[358] Rite, I., Machado, A., Cano, J. & Venero, J. L. Blood–brain barrier disruption induces in vivo degeneration of nigral dopaminergic neurons. *J. Neurochem.* **101**, 1567–1582 (2007). 17437543 10.1111/j.1471-4159.2007.04567.x

[359] Gray, M. T. & Woulfe, J. M. Striatal blood–brain barrier permeability in Parkinson’s disease. *J. Cereb. Blood Flow Metab.* **35**, 747–750 (2015). 25757748 10.1038/jcbfm.2015.32 PMC4420870

[360] Kortekaas, R. et al. Blood–brain barrier dysfunction in Parkinsonian midbrain in vivo. *Ann. Neurol.* **57**, 176–179 (2005). 15668963 10.1002/ana.20369

[361] Al-Bachari, S., Naish, J. H., Parker, G. J. M., Emsley, H. C. A. & Parkes, L. M. Blood–brain barrier leakage is increased in Parkinson’s disease. *Front. Physiol.* **11**, 593026 (2020). 33414722 10.3389/fphys.2020.593026 PMC7784911

[362] Nicaise, C. et al. Aquaporin-4 overexpression in Rat ALS model. *Anat. Rec.* **292**, 207–213 (2009). 10.1002/ar.20838 19089902

[363] Miyazaki, K. et al. Disruption of the neurovascular unit prior to motor neuron degeneration in amyotrophic lateral sclerosis. *J. Neurosci. Res.* **89**, 718–728 (2011). 21337372 10.1002/jnr.22594

[364] Waters, S. et al. Blood-spinal cord barrier leakage is independent of motor neuron pathology in ALS. *Acta Neuropathol. Commun.* **9**, 144 (2021). 34446086 10.1186/s40478-021-01244-0 PMC8393479

[365] Garbuzova-Davis, S. et al. Impaired blood–brain/spinal cord barrier in ALS patients. *Brain Res.* **1469**, 114–128 (2012). 22750125 10.1016/j.brainres.2012.05.056

[366] Vu, L. T. & Bowser, R. Fluid-based biomarkers for amyotrophic lateral sclerosis. *Neurotherapeutics* **14**, 119–134 (2017). 27933485 10.1007/s13311-016-0503-x PMC5233638

[367] Garbuzova-Davis, S. & Sanberg, P. R. Blood-CNS barrier impairment in ALS patients versus an animal model. *Front. Cell. Neurosci.* **8**, 21 (2014). 24550780 10.3389/fncel.2014.00021 PMC3910123

[368] Mastorakos, P. & McGavern, D. The anatomy and immunology of vasculature in the central nervous system. *Sci. Immunol.* **4**, eaav0492 (2019). 31300479 10.1126/sciimmunol.aav0492 PMC6816468

[369] Alves De Lima, K., Rustenhoven, J. & Kipnis, J. Meningeal immunity and its function in maintenance of the central nervous system in health and disease. *Annu. Rev. Immunol.* **38**, 597–620 (2020). 32340575 10.1146/annurev-immunol-102319-103410

[370] Bennett, H. C. et al. Aging drives cerebrovascular network remodeling and functional changes in the mouse brain. *Nat. Commun.* **15**, 6398 (2024). 39080289 10.1038/s41467-024-50559-8 PMC11289283

[371] Scuteri, A. et al. Longitudinal perspective on the conundrum of central arterial stiffness, blood pressure, and aging. *Hypertension* **64**, 1219–1227 (2014). 25225210 10.1161/HYPERTENSIONAHA.114.04127 PMC4231012

[372] Cai, C. et al. Impaired dynamics of precapillary sphincters and pericytes at first-order capillaries predict reduced neurovascular function in the aging mouse brain. *Nat. Aging* **3**, 173–184 (2023). 37118115 10.1038/s43587-022-00354-1 PMC11081516

[373] Yang, A. C. et al. Physiological blood–brain transport is impaired with age by a shift in transcytosis. *Nature* **583**, 425–430 (2020). 32612231 10.1038/s41586-020-2453-z PMC8331074

[374] Da Mesquita, S. et al. Functional aspects of meningeal lymphatics in ageing and Alzheimer’s disease. *Nature* **560**, 185–191 (2018). 30046111 10.1038/s41586-018-0368-8 PMC6085146

[375] Harvey, A., Montezano, A. C. & Touyz, R. M. Vascular biology of ageing—Implications in hypertension. *J. Mol. Cell. Cardiol.* **83**, 112–121 (2015). 25896391 10.1016/j.yjmcc.2015.04.011 PMC4534766

[376] Buford, T. W. Hypertension and aging. *Ageing Res. Rev.* **26**, 96–111 (2016). 26835847 10.1016/j.arr.2016.01.007 PMC4768730

[377] Holstein-Rønsbo, S. et al. Glymphatic influx and clearance are accelerated by neurovascular coupling. *Nat. Neurosci.* **26**, 1042–1053 (2023). 37264158 10.1038/s41593-023-01327-2 PMC10500159

[378] Faraco, G., Park, L., Anrather, J. & Iadecola, C. Brain perivascular macrophages: characterization and functional roles in health and disease. *J. Mol. Med.* **95**, 1143–1152 (2017). 28782084 10.1007/s00109-017-1573-x PMC5812456

[379] Van Hove, H. et al. A single-cell atlas of mouse brain macrophages reveals unique transcriptional identities shaped by ontogeny and tissue environment. *Nat. Neurosci.* **22**, 1021–1035 (2019). 31061494 10.1038/s41593-019-0393-4

[380] Masuda, T. et al. Specification of CNS macrophage subsets occurs postnatally in defined niches. *Nature* **604**, 740–748 (2022). 35444273 10.1038/s41586-022-04596-2

[381] Utz, S. G. et al. Early fate defines microglia and non-parenchymal brain macrophage development. *Cell* **181**, 557–573.e18 (2020). 32259484 10.1016/j.cell.2020.03.021

[382] Drieu, A. et al. Parenchymal border macrophages regulate tau pathology and tau-mediated neurodegeneration. *Life Sci. Alliance* **6**, e202302087 (2023). 37562846 10.26508/lsa.202302087 PMC10415611

[383] Iyonaga, T., Shinohara, K., Mastuura, T., Hirooka, Y. & Tsutsui, H. Brain perivascular macrophages contribute to the development of hypertension in stroke-prone spontaneously hypertensive rats via sympathetic activation. *Hypertens. Res.* **43**, 99–110 (2020). 31541222 10.1038/s41440-019-0333-4

[384] Mrdjen, D. et al. High-dimensional single-cell mapping of central nervous system immune cells reveals distinct myeloid subsets in health, aging, and disease. *Immunity* **48**, 599 (2018). 29562204 10.1016/j.immuni.2018.02.014

[385] Drieu, A. et al. Parenchymal border macrophages regulate the flow dynamics of the cerebrospinal fluid. *Nature* **611**, 585–593 (2022). 36352225 10.1038/s41586-022-05397-3 PMC9899827

[386] Levard, D. et al. Central nervous system-associated macrophages modulate the immune response following stroke in aged mice. *Nat. Neurosci.* **27**, 1721–1733 (2024). 38961228 10.1038/s41593-024-01695-3

[387] Mato, M. & Ookawara, S. Influences of age and vasopressin on the uptake capacity of fluorescent granular perithelial cells (FGP) of small cerebral vessels of the rat. *Am. J. Anat.* **162**, 45–53 (1981). 7304474 10.1002/aja.1001620105

[388] Rego, S., Sanchez, G. & Da Mesquita, S. Current views on meningeal lymphatics and immunity in aging and Alzheimer’s disease. *Mol. Neurodegener.* **18**, 55 (2023). 37580702 10.1186/s13024-023-00645-0 PMC10424377

[389] Ahn, J. H. et al. Meningeal lymphatic vessels at the skull base drain cerebrospinal fluid. *Nature* **572**, 62–66 (2019). 31341278 10.1038/s41586-019-1419-5

[390] Albayram, M. S. et al. Non-invasive MR imaging of human brain lymphatic networks with connections to cervical lymph nodes. *Nat. Commun.* **13**, 203 (2022). 35017525 10.1038/s41467-021-27887-0 PMC8752739

[391] Rustenhoven, J. et al. Age-related alterations in meningeal immunity drive impaired CNS lymphatic drainage. *J. Exp. Med.* **220**, e20221929 (2023). 37027179 10.1084/jem.20221929 PMC10083715

[392] Du, T. et al. Restoration of cervical lymphatic vessel function in aging rescues cerebrospinal fluid drainage. *Nat. Aging* **4**, 1418–1431 (2024). 39147980 10.1038/s43587-024-00691-3

[393] Nedergaard, M. & Goldman, S. A. Glymphatic failure as a final common pathway to dementia. *Science* **370**, 50–56 (2020). 33004510 10.1126/science.abb8739 PMC8186542

[394] Da Mesquita, S. et al. Meningeal lymphatics affect microglia responses and anti-Aβ immunotherapy. *Nature* **593**, 255–260 (2021). 33911285 10.1038/s41586-021-03489-0 PMC8817786

[395] Rustenhoven, J. et al. Functional characterization of the dural sinuses as a neuroimmune interface. *Cell* **184**, 1000–1016.e27 (2021). 33508229 10.1016/j.cell.2020.12.040 PMC8487654

[396] Brioschi, S. et al. Heterogeneity of meningeal B cells reveals a lymphopoietic niche at the CNS borders. *Science* **373**, eabf9277 (2021). 34083450 10.1126/science.abf9277 PMC8448524

[397] Rustenhoven, J. & Kipnis, J. Brain borders at the central stage of neuroimmunology. *Nature* **612**, 417–429 (2022). 36517712 10.1038/s41586-022-05474-7 PMC10205171

[398] Bloom, S. I., Islam, M. T., Lesniewski, L. A. & Donato, A. J. Mechanisms and consequences of endothelial cell senescence. *Nat. Rev. Cardiol.* **20**, 38–51 (2023). 35853997 10.1038/s41569-022-00739-0 PMC10026597

[399] Erusalimsky, J. D. Vascular endothelial senescence: from mechanisms to pathophysiology. *J. Appl. Physiol.* **106**, 326–332 (2009). 19036896 10.1152/japplphysiol.91353.2008 PMC2636933

[400] Chen, C.-Y. et al. Exosomal DMBT1 from human urine-derived stem cells facilitates diabetic wound repair by promoting angiogenesis. *Theranostics* **8**, 1607–1623 (2018). 29556344 10.7150/thno.22958 PMC5858170

[401] Xiao, X. et al. Mesenchymal stem cell-derived small extracellular vesicles mitigate oxidative stress-induced senescence in endothelial cells via regulation of miR-146a/Src. *Sig. Transduct. Target. Ther.* **6**, 354 (2021). 10.1038/s41392-021-00765-3 PMC8531331 34675187

[402] Maritz, M., Fourie, C. M. T., Van Rooyen, J. M. & Schutte, A. E. Evaluating several biomarkers as predictors of aortic stiffness in young and older Africans, not consuming alcohol based on self-report. *Diabetes Res. Clin. Pract.* **142**, 312–320 (2018). 29906479 10.1016/j.diabres.2018.05.048

[403] Tai, G.-J. et al. NLRP3 inflammasome-mediated premature immunosenescence drives diabetic vascular aging dependent on the induction of perivascular adipose tissue dysfunction. *Cardiovasc. Res.* **121**, 77–96 (2025). 38643484 10.1093/cvr/cvae079

[404] Bar, A. et al. Effects of life-long hyperlipidaemia on age-dependent development of endothelial dysfunction in humanised dyslipidaemic mice. *GeroScience* **47**, 2673–2701 (2025). 40240752 10.1007/s11357-025-01578-w PMC12181563

[405] Ahmed, B., Rahman, A. A., Lee, S. & Malhotra, R. The implications of aging on vascular health. *Int. J. Mol. Sci.* **25**, 11188 (2024). 39456971 10.3390/ijms252011188 PMC11508873

[406] Hayflick, L. The limited in vitro lifetime of human diploid cell strains. *Exp. Cell. Res.* **37**, 614–636 (1965). 14315085 10.1016/0014-4827(65)90211-9

[407] Wang, B., Han, J., Elisseeff, J. H. & Demaria, M. The senescence-associated secretory phenotype and its physiological and pathological implications. *Nat. Rev. Mol. Cell Biol.* **25**, 958–978 (2024). 38654098 10.1038/s41580-024-00727-x

[408] Roos, C. M. et al. Chronic senolytic treatment alleviates established vasomotor dysfunction in aged or atherosclerotic mice. *Aging Cell* **15**, 973–977 (2016). 26864908 10.1111/acel.12458 PMC5013022

[409] Suda, M. et al. Senolytic vaccination improves normal and pathological age-related phenotypes and increases lifespan in progeroid mice. *Nat. Aging* **1**, 1117–1126 (2021). 37117524 10.1038/s43587-021-00151-2

[410] Zhu, Y. et al. The Achilles’ heel of senescent cells: from transcriptome to senolytic drugs. *Aging Cell* **14**, 644–658 (2015). 25754370 10.1111/acel.12344 PMC4531078

[411] Martin-Ruiz, C. et al. CMV-independent increase in CD27−CD28+ CD8+ EMRA T cells is inversely related to mortality in octogenarians. *npj Aging Mech. Dis.* **6**, 3 (2020). 31993214 10.1038/s41514-019-0041-y PMC6972903

[412] McHugh, D., Durán, I. & Gil, J. Senescence as a therapeutic target in cancer and age-related diseases. *Nat. Rev. Drug Discov.* **24**, 57–71 (2025). 39548312 10.1038/s41573-024-01074-4

[413] Bracken, O. V., De Maeyer, R. P. H. & Akbar, A. N. Enhancing immunity during ageing by targeting interactions within the tissue environment. *Nat. Rev. Drug Discov.* **24**, 300–315 (2025). 39875569 10.1038/s41573-024-01126-9

[414] Zhang, L., Pitcher, L. E., Prahalad, V., Niedernhofer, L. J. & Robbins, P. D. Targeting cellular senescence with senotherapeutics: senolytics and senomorphics. *FEBS J* **290**, 1362–1383 (2023). 35015337 10.1111/febs.16350

[415] Bi, Y. et al. Exosomal miR-302b rejuvenates aging mice by reversing the proliferative arrest of senescent cells. *Cell Metab* **37**, 527–541.e6 (2025). 39818209 10.1016/j.cmet.2024.11.013

[416] Wang, C. et al. Inducing and exploiting vulnerabilities for the treatment of liver cancer. *Nature* **574**, 268–272 (2019). 31578521 10.1038/s41586-019-1607-3 PMC6858884

[417] Laberge, R.-M. et al. MTOR regulates the pro-tumorigenic senescence-associated secretory phenotype by promoting IL1A translation. *Nat. Cell. Biol.* **17**, 1049–1061 (2015). 26147250 10.1038/ncb3195 PMC4691706

[418] Herranz, N. et al. mTOR regulates MAPKAPK2 translation to control the senescence-associated secretory phenotype. *Nat. Cell. Biol.* **17**, 1205–1217 (2015). 26280535 10.1038/ncb3225 PMC4589897

[419] Pospelova, T. V. et al. Suppression of replicative senescence by rapamycin in rodent embryonic cells. *Cell Cycle* **11**, 2402–2407 (2012). 22672902 10.4161/cc.20882

[420] Xu, Q. et al. The flavonoid procyanidin C1 has senotherapeutic activity and increases lifespan in mice. *Nat. Metab.* **3**, 1706–1726 (2021). 34873338 10.1038/s42255-021-00491-8 PMC8688144

[421] Grosse, L. et al. Defined p16 high senescent cell types are indispensable for mouse healthspan. *Cell Metab* **32**, 87–99.e6 (2020). 32485135 10.1016/j.cmet.2020.05.002

[422] Liu, J.-Y. et al. Cells exhibiting strong *p16*^*INK4a*^ promoter activation in vivo display features of senescence. *Proc. Natl. Acad. Sci. USA* **116**, 2603–2611 (2019). 30683717 10.1073/pnas.1818313116 PMC6377452

[423] Sun, J., Wang, Y. N., Luo, S. S. & Liu, B. H. Advances in research on real-time monitoring of aging reporter mouse models. *Acta Physiol. Sin.* **75**, 836–846 (2023). 38151347

[424] Hori, N. et al. Shaving black fur uncovers hidden issues in p16-3MR mice. 2024.06.24.600181 Preprint at 10.1101/2024.06.24.600181 (2024).

[425] Chandra, A. et al. Targeted clearance of *p21* - but not *p16* -positive senescent cells prevents radiation-induced osteoporosis and increased marrow adiposity. *Aging Cell* **21**, e13602 (2022). 35363946 10.1111/acel.13602 PMC9124310

[426] Wang, B. et al. An inducible p21-Cre mouse model to monitor and manipulate p21-highly-expressing senescent cells in vivo. *Nat. Aging* **1**, 962–973 (2021). 35024619 10.1038/s43587-021-00107-6 PMC8746571

[427] Mikawa, R. et al. Elimination of p19^ARF^-expressing cells protects against pulmonary emphysema in mice. *Aging Cell* **17**, e12827 (2018). 30058137 10.1111/acel.12827 PMC6156494

[428] Farr, J. N. et al. Targeting cellular senescence prevents age-related bone loss in mice. *Nat. Med.* **23**, 1072–1079 (2017). 28825716 10.1038/nm.4385 PMC5657592

[429] Wang, X. et al. Signalling by senescent melanocytes hyperactivates hair growth. *Nature* **618**, 808–817 (2023). 37344645 10.1038/s41586-023-06172-8 PMC10284692

[430] Born, E. et al. Eliminating senescent cells can promote pulmonary hypertension development and progression. *Circulation* **147**, 650–666 (2023). 36515093 10.1161/CIRCULATIONAHA.122.058794

[431] Pu, W. et al. Bipotent transitional liver progenitor cells contribute to liver regeneration. *Nat. Genet.* **55**, 651–664 (2023). 36914834 10.1038/s41588-023-01335-9 PMC10101857

[432] De, D., Karmakar, P. & Bhattacharya, D. Stem cell aging and regenerative medicine. *Adv. Exp. Med. Biol.* **1326**, 11–37 (2021). 32910426 10.1007/5584_2020_577

[433] Hasegawa, T. et al. Cytotoxic CD4+ T cells eliminate senescent cells by targeting cytomegalovirus antigen. *Cell* **186**, 1417–1431.e20 (2023). 37001502 10.1016/j.cell.2023.02.033

[434] Ovadya, Y. et al. Impaired immune surveillance accelerates accumulation of senescent cells and aging. *Nat. Commun.* **9**, 5435 (2018). 30575733 10.1038/s41467-018-07825-3 PMC6303397

[435] Kang, T.-W. et al. Senescence surveillance of pre-malignant hepatocytes limits liver cancer development. *Nature* **479**, 547–551 (2011). 22080947 10.1038/nature10599

[436] Muñoz, D. P. et al. Targetable mechanisms driving immunoevasion of persistent senescent cells link chemotherapy-resistant cancer to aging. *J. Clin. Investig. Insight* **4**, e124716 (2019). 10.1172/jci.insight.124716 PMC6675550 31184599

[437] Shahbandi, A. et al. Breast cancer cells survive chemotherapy by activating targetable immune-modulatory programs characterized by PD-L1 or CD80. *Nat. Cancer* **3**, 1513–1533 (2022). 36482233 10.1038/s43018-022-00466-y PMC9923777

[438] Pereira, B. I. et al. Senescent cells evade immune clearance via HLA-E-mediated NK and CD8+ T cell inhibition. *Nat. Commun.* **10**, 2387 (2019). 31160572 10.1038/s41467-019-10335-5 PMC6547655

[439] Chaib, S. et al. The efficacy of chemotherapy is limited by intratumoral senescent cells expressing PD-L2. *Nat. Cancer* **5**, 448–462 (2024). 38267628 10.1038/s43018-023-00712-x PMC10965441

[440] Robert, C. A decade of immune-checkpoint inhibitors in cancer therapy. *Nat. Commun.* **11**, 3801 (2020). 32732879 10.1038/s41467-020-17670-y PMC7393098

[441] Amor, C. et al. Senolytic CAR T cells reverse senescence-associated pathologies. *Nature* **583**, 127–132 (2020). 32555459 10.1038/s41586-020-2403-9 PMC7583560

[442] Kim, K. M. et al. Identification of senescent cell surface targetable protein DPP4. *Genes. Dev.* **31**, 1529–1534 (2017). 28877934 10.1101/gad.302570.117 PMC5630018

[443] Mannick, J. B. & Lamming, D. W. Targeting the biology of aging with mTOR inhibitors. *Nat. Aging* **3**, 642–660 (2023). 37142830 10.1038/s43587-023-00416-y PMC10330278

[444] Völkers, M. et al. PRAS40 prevents development of diabetic cardiomyopathy and improves hepatic insulin sensitivity in obesity. *EMBO Mol. Med.* **6**, 57–65 (2014). 24408966 10.1002/emmm.201303183 PMC3936489

[445] Mannick, J. B. et al. mTOR inhibition improves immune function in the elderly. *Sci. Transl. Med.* **6**, 268ra179 (2014). 25540326 10.1126/scitranslmed.3009892

[446] Tong, C. et al. Impaired SIRT1 nucleocytoplasmic shuttling in the senescent heart during ischemic stress. *FASEB j* **27**, 4332–4342 (2013). 23024374 10.1096/fj.12-216473 PMC3804750

[447] Barcena De Arellano, M. L. et al. Sex differences in the aging human heart: decreased sirtuins, pro-inflammatory shift and reduced anti-oxidative defense. *Aging* **11**, 1918–1933 (2019). 30964749 10.18632/aging.101881 PMC6503880

[448] Ogawa, T. et al. Distribution of the longevity gene product, SIRT1, in developing mouse organs. *Congenital Anomalies* **51**, 70–79 (2011). 21054562 10.1111/j.1741-4520.2010.00304.x

[449] Alcendor, R. R. et al. Sirt1 regulates aging and resistance to oxidative stress in the heart. *Circ. Res.* **100**, 1512–1521 (2007). 17446436 10.1161/01.RES.0000267723.65696.4a

[450] Packer, M. Longevity genes, cardiac ageing, and the pathogenesis of cardiomyopathy: implications for understanding the effects of current and future treatments for heart failure. *Eur. Heart J.* **41**, 3856–3861 (2020). 32460327 10.1093/eurheartj/ehaa360 PMC7599035

[451] Tanno, M. et al. Induction of manganese superoxide dismutase by nuclear translocation and activation of SIRT1 promotes cell survival in chronic heart failure. *J. Biol. Chem.* **285**, 8375–8382 (2010). 20089851 10.1074/jbc.M109.090266 PMC2832987

[452] Yuan, Y.-P. et al. CTRP3 protected against doxorubicin-induced cardiac dysfunction, inflammation and cell death via activation of Sirt1. *J. Mol. Cell. Cardiol.* **114**, 38–47 (2018). 29061338 10.1016/j.yjmcc.2017.10.008

[453] Bailey, H. H. et al. A randomized, double-blind, dose-ranging, pilot trial of piperine with resveratrol on the effects on serum levels of resveratrol. *Eur. J. Cancer Prev.* **30**, 285–290 (2021). 32868637 10.1097/CEJ.0000000000000621 PMC7910313

[454] Martin-Montalvo, A. et al. Metformin improves healthspan and lifespan in mice. *Nat. Commun.* **4**, 2192 (2013). 23900241 10.1038/ncomms3192 PMC3736576

[455] Ramsey, K. M., Mills, K. F., Satoh, A. & Imai, S. Age-associated loss of Sirt1-mediated enhancement of glucose-stimulated insulin secretion in beta cell-specific Sirt1-overexpressing (BESTO) mice. *Aging Cell* **7**, 78–88 (2008). 18005249 10.1111/j.1474-9726.2007.00355.x PMC2238677

[456] Strong, R. et al. Longer lifespan in male mice treated with a weakly estrogenic agonist, an antioxidant, an α-glucosidase inhibitor or a Nrf2-inducer. *Aging Cell* **15**, 872–884 (2016). 27312235 10.1111/acel.12496 PMC5013015

[457] Barzilai, N., Crandall, J. P., Kritchevsky, S. B. & Espeland, M. A. Metformin as a tool to target aging. *Cell Metab* **23**, 1060–1065 (2016). 27304507 10.1016/j.cmet.2016.05.011 PMC5943638

[458] Valencia, W. M., Palacio, A., Tamariz, L. & Florez, H. Metformin and ageing: improving ageing outcomes beyond glycaemic control. *Diabetologia* **60**, 1630–1638 (2017). 28770328 10.1007/s00125-017-4349-5 PMC5709209

[459] Campbell, J. M., Bellman, S. M., Stephenson, M. D. & Lisy, K. Metformin reduces all-cause mortality and diseases of ageing independent of its effect on diabetes control: a systematic review and meta-analysis. *Ageing Res. Rev.* **40**, 31–44 (2017). 28802803 10.1016/j.arr.2017.08.003

[460] McCreight, L. J., Bailey, C. J. & Pearson, E. R. Metformin and the gastrointestinal tract. *Diabetologia* **59**, 426–435 (2016). 26780750 10.1007/s00125-015-3844-9 PMC4742508

[461] Yan, Y., Thakur, M., van der Vorst, E. P. C., Weber, C. & Döring, Y. Targeting the chemokine network in atherosclerosis. *Atherosclerosis* **330**, 95–106 (2021). 34247863 10.1016/j.atherosclerosis.2021.06.912

[462] Guo, D., Zhu, W. & Qiu, H. C-C motif chemokine ligand 2 and chemokine receptor 2 in cardiovascular and neural aging and aging-related diseases. *Int. J. Mol. Sci.* **25**, 8794 (2024). 39201480 10.3390/ijms25168794 PMC11355023

[463] McNeil, J. J. et al. Effect of aspirin on cardiovascular events and bleeding in the healthy elderly. *N. Engl. J. Med.* **379**, 1509–1518 (2018). 30221597 10.1056/NEJMoa1805819 PMC6289056

[464] Kaltsonoudis, E., Pelechas, E., Voulgari, P. V. & Drosos, A. A. Neuroinflammatory events after anti-TNFα therapy. *Ann. Rheum. Dis.* **81**, e73 (2022). 32434825 10.1136/annrheumdis-2020-217723

[465] Cappuccio, F. P., Cooper, D., D’Elia, L., Strazzullo, P. & Miller, M. A. Sleep duration predicts cardiovascular outcomes: A systematic review and meta-analysis of prospective studies. *Eur. Heart J.* **32**, 1484–1492 (2011). 21300732 10.1093/eurheartj/ehr007

[466] Huynh, P. et al. Myocardial infarction augments sleep to limit cardiac inflammation and damage. *Nature* **635**, 168–177 (2024). 39478215 10.1038/s41586-024-08100-w PMC11998484

[467] Welberg, L. The brain heals the heart. *Nat. Neurosci.* **27**, 1638 (2024). 39237819 10.1038/s41593-024-01761-w

[468] Dai, N. et al. Stress-related neural activity associates with coronary plaque vulnerability and subsequent cardiovascular events. *JACC Cardiovasc. Imaging* **16**, 1404–1415 (2023). 37269269 10.1016/j.jcmg.2023.04.004

[469] Guarente, L., Sinclair, D. A. & Kroemer, G. Human trials exploring anti-aging medicines. *Cell Metab.* **36**, 354–376 (2024). 38181790 10.1016/j.cmet.2023.12.007

[470] Peng, W., Zhou, R., Sun, Z.-F., Long, J.-W. & Gong, Y.-Q. Novel insights into the roles and mechanisms of GLP-1 receptor agonists against aging-related diseases. *Aging Dis* **13**, 468–490 (2022). 35371594 10.14336/AD.2021.0928 PMC8947838

[471] Ida, S. et al. Effects of antidiabetic drugs on muscle mass in type 2 diabetes mellitus. *Curr. Diabetes Rev.* **17**, 293–303 (2021). 32628589 10.2174/1573399816666200705210006

[472] Aiello, A. et al. Nutrigerontology: a key for achieving successful ageing and longevity. *Immun. Ageing* **13**, 17 (2016). s12979-016-0071–2. 27213002 10.1186/s12979-016-0071-2 PMC4875663

[473] Hu, F. B. Diet strategies for promoting healthy aging and longevity: an epidemiological perspective. *J. Intern. Med.* **295**, 508–531 (2024). 37867396 10.1111/joim.13728 PMC10939982

[474] Roh, J. D. et al. Exercise training reverses cardiac aging phenotypes associated with heart failure with preserved ejection fraction in male mice. *Aging Cell* **19**, e13159 (2020). 32441410 10.1111/acel.13159 PMC7294786

[475] Shibata, S. et al. Congestive heart failure with preserved ejection fraction is associated with severely impaired dynamic Starling mechanism. *J. Appl. Physiol.* **110**, 964–971 (2011). 21310890 10.1152/japplphysiol.00826.2010 PMC3075124

[476] Barzilai, N., Cuervo, A. M. & Austad, S. Aging as a biological target for prevention and therapy. *J. Am. Heart Assoc.* **320**, 1321–1322 (2018). 10.1001/jama.2018.9562 30242337

[477] Oxenham, H. & Sharpe, N. Cardiovascular aging and heart failure. *Eur. J. Heart Fail.* **5**, 427–434 (2003). 12921803 10.1016/s1388-9842(03)00011-4

[478] Moqri, M. et al. Validation of biomarkers of aging. *Nat. Med.* **30**, 360–372 (2024). 38355974 10.1038/s41591-023-02784-9 PMC11090477

[479] Li, J. et al. Determining a multimodal aging clock in a cohort of Chinese women. *Medicines* **4**, 825–848.e13 (2023). 10.1016/j.medj.2023.06.010 37516104

[480] Zheng, Z. et al. DNA methylation clocks for estimating biological age in Chinese cohorts. *Protein Cell* **15**, 575–593 (2024). 38482631 10.1093/procel/pwae011 PMC11259550

[481] Ying, K. et al. Causality-enriched epigenetic age uncouples damage and adaptation. *Nat. Aging* **4**, 231–246 (2024). 38243142 10.1038/s43587-023-00557-0 PMC11070280

[482] Pośpiech, E. et al. Epigenetic clock in the aorta and age-related endothelial dysfunction in mice. *GeroScience* **46**, 3993–4002 (2024). 38381284 10.1007/s11357-024-01086-3 PMC11226569

[483] Oh, H. S.-H. et al. Organ aging signatures in the plasma proteome track health and disease. *Nature* **624**, 164–172 (2023). 38057571 10.1038/s41586-023-06802-1 PMC10700136

[484] Saul, D. et al. A new gene set identifies senescent cells and predicts senescence-associated pathways across tissues. *Nat. Commun.* **13**, 4827 (2022). 35974106 10.1038/s41467-022-32552-1 PMC9381717

[485] Wang, X.-M., Wang, H.-P., Chen, H.-Z. & Liu, D.-P. Epigenetic clock: future of hypertension prediction? *Hypertension* **80**, 1569–1571 (2023). 37470774 10.1161/HYPERTENSIONAHA.123.21197

[486] Hu, F. B. Obesity in the USA: diet and lifestyle key to prevention. *Lancet Diabetes Endocrinol* **11**, 642–643 (2023). 37459874 10.1016/S2213-8587(23)00194-8

[487] Seara, F. A. C., Kasai-Brunswick, T. H., Nascimento, J. H. M. & Campos-de-Carvalho, A. C. Anthracycline-induced cardiotoxicity and cell senescence: new therapeutic option? *Cell. Mol. Life Sci.* **79**, 568 (2022). 36287277 10.1007/s00018-022-04605-7 PMC11803035

